# Deciphering the Developmental Dynamics of the Mouse Liver Transcriptome

**DOI:** 10.1371/journal.pone.0141220

**Published:** 2015-10-23

**Authors:** Sumedha S. Gunewardena, Byunggil Yoo, Lai Peng, Hong Lu, Xiaobo Zhong, Curtis D. Klaassen, Julia Yue Cui

**Affiliations:** 1 Department of Molecular and Integrative Physiology, Department of Biostatistics, University of Kansas Medical Center, Kansas City, Kansas, 66160, United States of America; 2 Children's Mercy Hospital, Kansas City, Missouri, 64108, United States of America; 3 Department of Pharmaceutical Sciences, School of Pharmacy, University of Connecticut, Storrs, Connecticut, 06269, United States of America; 4 Department of Pharmacology, State University of New York Upstate Medical University, Syracuse, New York, 13210, United States of America; 5 Department of Environmental and Occupational Health Sciences, University of Washington, Seattle, Washington, 98195, United States of America; International Centre for Genetic Engineering and Biotechnology, ITALY

## Abstract

During development, liver undergoes a rapid transition from a hematopoietic organ to a major organ for drug metabolism and nutrient homeostasis. However, little is known on a transcriptome level of the genes and RNA-splicing variants that are differentially regulated with age, and which up-stream regulators orchestrate age-specific biological functions in liver. We used RNA-Seq to interrogate the developmental dynamics of the liver transcriptome in mice at 12 ages from late embryonic stage (2-days before birth) to maturity (60-days after birth). Among 21,889 unique NCBI RefSeq-annotated genes, 9,641 were significantly expressed in at least one age, 7,289 were differently regulated with age, and 859 had multiple (> = 2) RNA splicing-variants. Factor analysis showed that the dynamics of hepatic genes fall into six distinct groups based on their temporal expression. The average expression of cytokines, ion channels, kinases, phosphatases, transcription regulators and translation regulators decreased with age, whereas the average expression of peptidases, enzymes and transmembrane receptors increased with age. The average expression of growth factors peak between Day-3 and Day-10, and decrease thereafter. We identified critical biological functions, upstream regulators, and putative transcription modules that seem to govern age-specific gene expression. We also observed differential ontogenic expression of known splicing variants of certain genes, and 1,455 novel splicing isoform candidates. In conclusion, the hepatic ontogeny of the transcriptome ontogeny has unveiled critical networks and up-stream regulators that orchestrate age-specific biological functions in liver, and suggest that age contributes to the complexity of the alternative splicing landscape of the hepatic transcriptome.

## Introduction

Liver is an essential organ for drug metabolism and nutrient homeostasis. For example, the superfamily of cytochrome P450s, many of which are highly expressed in liver, are responsible for metabolizing more than 80% of the prescribed drugs in humans [1]. Genetic polymorphisms and inborn errors of various drug-metabolizing enzymes and transporters in liver have been linked to inter-individual variations in drug response and adverse drug reactions [2]. Besides its critical role in processing drugs, liver has many important physiological functions. For example, an essential role of liver is to convert cholesterol into bile acids, which are critical signaling molecules involved in regulating bile flow [3], obesity and diabetes [4–6], as well as energy expenditure [7]. In addition, liver is critically involved in carbohydrate metabolism, such as glycogenesis, glycogenolysis, and gluconeogenesis; fat metabolism, such as triglyceride oxidation and lipoprotein synthesis [8, 9]; hormonal responses [10], as well as synthesis of plasma proteins, such as albumin and clotting factors [11, 12]. Because liver has such a broad spectrum of critical physiological and pharmacological functions, malfunction of liver not only leads to hepatic injuries such as cholestasis, cirrhosis, and liver cancer [13], but also produces profound deleterious effects on a systemic level such as malnutrition [14], hepatic encephalopathy [15], growth retardation [13], and osteoporosis [16, 17].

During liver development, profound changes occur in the expression of isoforms of critical genes involved in processing dietary nutrients, drugs and other xenobiotics. This subsequently alters the absorption, distribution, metabolism, and excretion of various chemicals, and markedly affects the risks of adverse drug reactions in newborns and children [18–20]. However, although much research has been performed to determine physiological and pharmacological pathways in adult liver, much less is known about the ontogeny of genes involved in nutrient homeostasis and drug disposition during liver development. Therefore, children are often referred to as “therapeutic orphans” [21–23]. During development, marked changes occur in cell types and functions of liver. Before birth, liver is a major site for fetal hematopoiesis whereas at the end of the gestational period, both hepatocytes and bile-duct epithelial cells develop from the bipotential hepatoblasts, and only a small proportion of cells are committed to hematopoiesis [24–27].

After birth the liver becomes the major organ for drug metabolism and nutrient homeostasis. The ontogeny of some important liver genes such as those encoding drug-metabolizing enzymes has been investigated during human liver development [28–30]. Recent research using animal models have discovered a growing list of evolutionarily conserved genes and signaling pathways that program liver development [31–33]. For example, a recent microarray study has shown that there are 4 apparent developmental stages during liver development in mice, and genes with stage-specific functions are often enhanced or inhibited at their corresponding phases [32]. Therefore, transcriptional profiling of liver genes at various ages in mice provides a useful tool to facilitate further investigations of known and novel gene signaling pathways that are important for normal liver development. Interestingly, the expression profiles of cell-proliferation and apoptosis-related genes during liver development share high similarities to hepatocellular carcinoma [32] and liver regeneration following partial hepatectomy [25].

Alternative splicing is a critical event in higher eukaryotes that leads to protein diversity from the genome, tissue specificity of gene expression, and age-specific signaling pathways [34]. A deep survey of alternative splicing complexity in the human transcriptome has shown that approximately 95% of multi-exon genes undergo alternative splicing, and liver is a major tissue that carries highly abundant alternative splicing events of transcripts [35]. Aberrant transcript variants are implicated in diseases such as liver cancer [36, 37]. During liver development, it has been shown in mice that hepatocyte nuclear factor 4 alpha (*Hnf4α*), which is an essential nuclear receptor for the establishment and maintenance of the expression of numerous liver-specific genes, undergoes a sequential expression from the isoform *Hnf4α7/a8* to *Hnf4α1/a2*, and this parallels the hepatic phenotype transition from fetal to adult [38]. However, the potential age-specific transcript variants of many essential liver genes are not characterized in vivo. Conventional mRNA profiling tools, such as Northern blot, real-time PCR (RT-PCR), and microarray, require gene-specific primers and cannot capture the profiles of alternative splicing events on a large scale. RNA-Seq (a next-generation sequencing technology associated with massively parallel sequencing of cDNA) provides an unbiased detection of transcript variants, and allows the discovery of novel transcripts on a transcriptome-wide scale [39, 40]. This research group has characterized the ontogeny of various drug metabolizing enzymes, transporters and epigenetic modifiers in mouse liver using RNA-Seq [41–43]. The present study will determine the dynamic changes in the abundance and isoforms of the entire transcriptome during liver development, with the goal to decipher critical signaling networks that are implicated in the postnatal development of the liver, and provide global insights into further understanding the physiology of normal liver development in mice.

## Results and Discussion

### Whole transcriptome shotgun sequencing

The present study uses RNA-Seq to interrogate the developmental dynamics of the mouse liver transcriptome sampled at 12 ages, Day -2 (GD17.5), Day 0 (right after birth and before the start of suckling), Day 1, 3, 5, 10, 15, 20, 25, 30, 45 and 60. Sequencing was performed in biological triplicates for each age, giving 36 samples in total. Each sample generated around 165 to 195 million 2x100bp paired end reads, with a median read count of 174 million per sample. On average, 83% of these reads were mapped to the mouse reference genome (NCBI37/mm9). The correlation of gene expression between replicate samples was very high, with a Pearson's correlation coefficient ranging between 0.94 and 0.99 with a median of 0.98. The consistency of the RNA-Seq data was evaluated by correlating RNA-Seq expression values of 7 randomly selected representative genes to their RT-PCR expression values obtained at the same 12 developmental ages. These validation genes included genes that are expressed during early stages of development (*Igf2*, *Gadd45a*, *Afp*), during middle stages of development (*Ccnd1*, *Nfkbie*) and during late stages of development (*Slco1b2*, *Bsep*). The correlation coefficients of these genes varied between 0.73 and 0.96 with a median correlation coefficient of 0.81 **(S1 Table)**.

This study complements the previously described microarray work by Li and colleagues [32] on mouse liver development with the advantages of RNA-Seq over the high-density oligonucleotide microarrays used in the previous study. RNA-Seq has inherent advantages compared to traditional microarray based studies, and is the best technology to date for obtaining an unbiased global perspective of transcriptomic changes during development [44–46]. Because RNA-Seq is not dependent on a reference (e.g. probes or primers) for transcript abundance estimation, it is able to detect previously uncharacterized transcriptional structures of genes such as novel exons, splice junctions, promoters and 3' untranslated regions [35, 47, 48]. Single-base level resolution of RNA-Seq enables the precise determination of exon boundaries [46, 49, 50]. RNA-Seq estimates a fragment count that is directly proportional to the amount of mRNA present in the sequenced sample providing an unlimited dynamic range for transcript abundance estimation, and an ability to detect very highly and very lowly expressed transcripts [45, 46]. It is also advantageous that the data generated by RNA-Seq is highly reproducible [45, 50]. While there is a high congruence in the results described in the two studies, the current study expands substantially the available knowledge on the developmental dynamics of the mouse liver transcriptome, and provides an invaluable resource to the research community.

The consistency of the RNA-Seq data was further validated by comparing gene expression across the two studies. The 4024 genes that were differentially expressed over time in both studies were used for the comparison. Because the two studies did not match exactly on the selected ages, only those ages that were relatively close to each other were considered. RNA-Seq expression at the 7 ages, Day -2, 0, 3, 5, 15, 20, and 60 from the current study was correlated with microarray expression at 7 corresponding ages, GD17, Day 0, 3, 7, 14, 21 and Adult from the Li et al. study. Although the matched ages were not exactly the same, the gene expression patterns of the two studies showed a median correlation coefficient of 0.72 demonstrating a relatively high congruence between the two studies. The distribution of the correlation coefficients of the 4024 genes is shown in **S1 Fig**.

### Gene expression analysis

**Fig 1:**
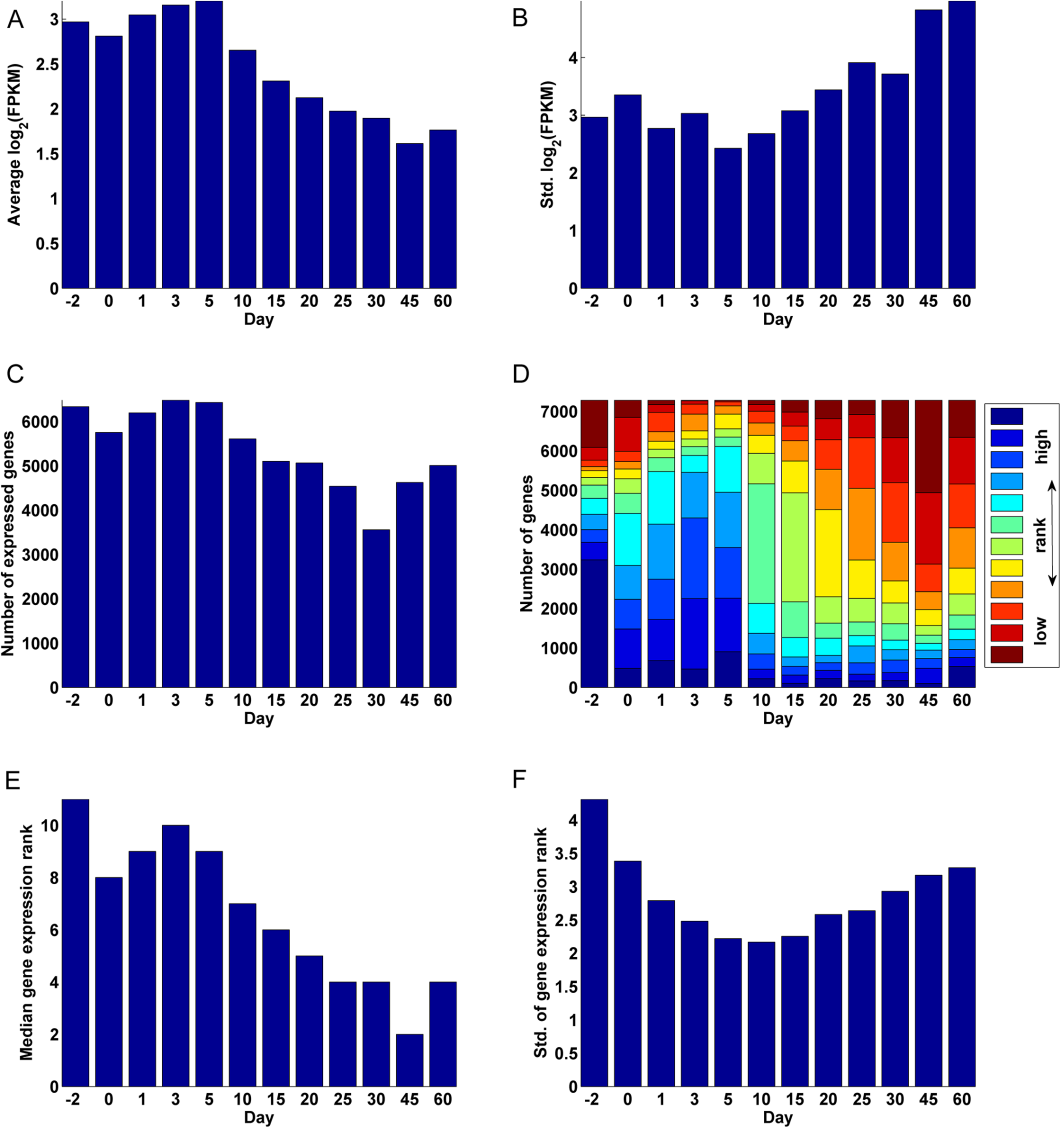
Gene expression statistics. (A) Average log_2_ FPKM values over all genes at the 12 ages. (B) Standard deviation of the log_2_ FPKM values at the 12 ages. (C) Total number of significantly expressed genes among the 7,289 significantly differentially expressed genes at the 12 ages. (D) Distribution of the gene expression rank (GER) of the 7,289 significantly differently expressed genes, describing the relative ranking of gene expression at the 12 ages. (E) The median gene expression rank (1–12) for each age. (F) The standard deviation of the gene expression rank (1–12) for each age.

The cumulative FPKM (Fragments Per Kilo base of transcript per Million) values of all genes were relatively similar across samples, ranging between 374k and 435k at the 12 selected ages **(S2 Fig)**. For this analysis, genes were first filtered on their absolute expression and then on their differential expression over time. Out of 21,889 unique NCBI RefSeq annotated genes, 7,289 genes were significantly expressed **(**see **S1 Methods** for details on how significant gene expression was determined) in at least one of the 12 analyzed ages and also significantly differently expressed **(**see **S1 Methods** for details on how significant differential gene expression was determined) over the 12 analyzed ages. These genes formed the basis for further analysis and are henceforth referred to as the *analysis-genes* **(S2 Table).** The average gene expression was highest during the early periods of development and decreased gradually after Day 5 **(Fig 1A)**. However the standard deviation in gene expression showed a gradual increase after Day 5 **(Fig 1B)**. These observations can be explained by the fact that a majority of genes expressed significantly during the early days of development, with the highest number of genes expressed on Day 3 (90% of 7289) and the lowest number of genes expressed on Day 30 (49% of 7289) (**Fig 1C)**. Although Day 3 had the highest number of expressed genes (**Fig 1C)**, Day -2 had the most number of genes at their highest level of expression, i.e. with the highest gene expression rank (GER: the rank, between 1 and 12, of a gene's relative expression over the 12 ages; see Methods), over the 12 ages (44% of the genes; **Fig 1D**). Day -2 also had the highest standard deviation in gene expression rank (**Fig 1F**). This signals a distinction in the genes expressed during the perinatal period of development especially before birth. The high standard deviation of the GER observed at Day -2 and to some extent at Day 0 is due to a specific set of genes being highly expressed while the other genes being lowly expressed on these days. In contrast, the standard deviation of the GER on Days 1–3 is relatively low, although their median GER is only below that of Day -2 (**Fig 1E**). A majority of the *analysis-genes* were at their lowest levels of expression on Day 45 (32%). The plot of the standard deviation of the GER over time assumed a parabolic shape, decreasing gradually form Day -2 to Day 10 and increasing gradually from there on to Day 60 (**Fig 1F**). The relatively low standard deviation in the GER around Days 5–15 can be attributed to a period of transition between genes that are expressed early in development and genes that are expressed late in development.

**Fig 2:**
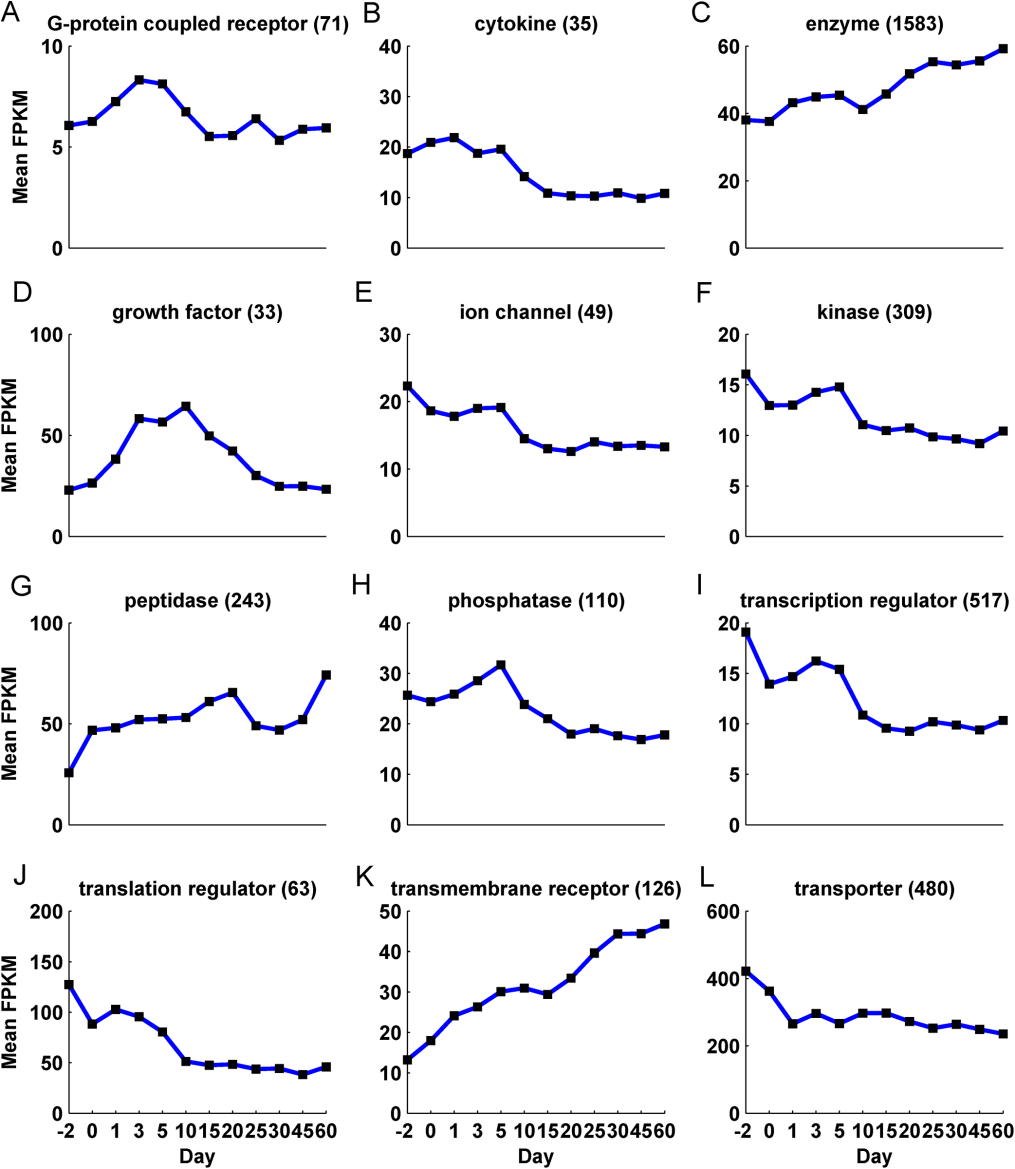
The average expression pattern of the different protein types over the 12 ages.

Enzymes were the most abundant type of protein making up 14% of all genes and 22% of the *analysis-genes* **(S3 Table)**. Other types of proteins such as transcription regulators (8%), transporters (7%) and kinases (5%) also showed a significant presence among the *analysis-genes* although they did not differ significantly from their initial proportions (7%, 5% and 4% respectively). Interestingly, although the number of significantly differentially expressed genes varied with age, ranging between a maximum of 6,484 genes on Day 3 and a minimum of 3,558 genes on Day 30, the proportion of the different molecule types associated with these genes did not change significantly (Chi-square test of independence of age and protein type *p-value* = 0.12; **S4 Table**). However, the cumulative expression associated with the different protein types changed significantly over time **(Fig 2)**. For example, the mean expression of the 480 transporter genes dropped from around 420 FPKM per gene on Day -2 to around 260 FPKM per gene on Day 1 and remained relatively constant through Day 60 (**Fig 2L, S5 Table**). Other protein types such as cytokines, ion channels, kinases, phosphatases, transcription regulators and translation regulators also showed a relatively high mean expression during early ages of development, which dropped after Day 5 **(Fig 2B, 2E, 2F, 2H, 2I & 2J**). In contrast, protein types such as peptidases, enzymes and transmembrane receptors increased in their mean expression over time **(Fig 2G, 2C & 2L)**. The mean expression of growth factors increased to peak between Day 3 and 10 and decreased thereafter **(Fig 2D)**. A majority of the *analysis-genes* were localized in the cytoplasm (38%) followed by the nucleus (23%), plasma membrane (13%), and extracellular space (7%) **(S3 Fig)**. Directed by these observations, a clustering based on factor analysis **(see Methods)** was performed on the *analysis-genes* to ascertain the fundamental patterns of gene expression associated with liver development.

### Differential expression of known alternative splicing transcripts

**Fig 3:**
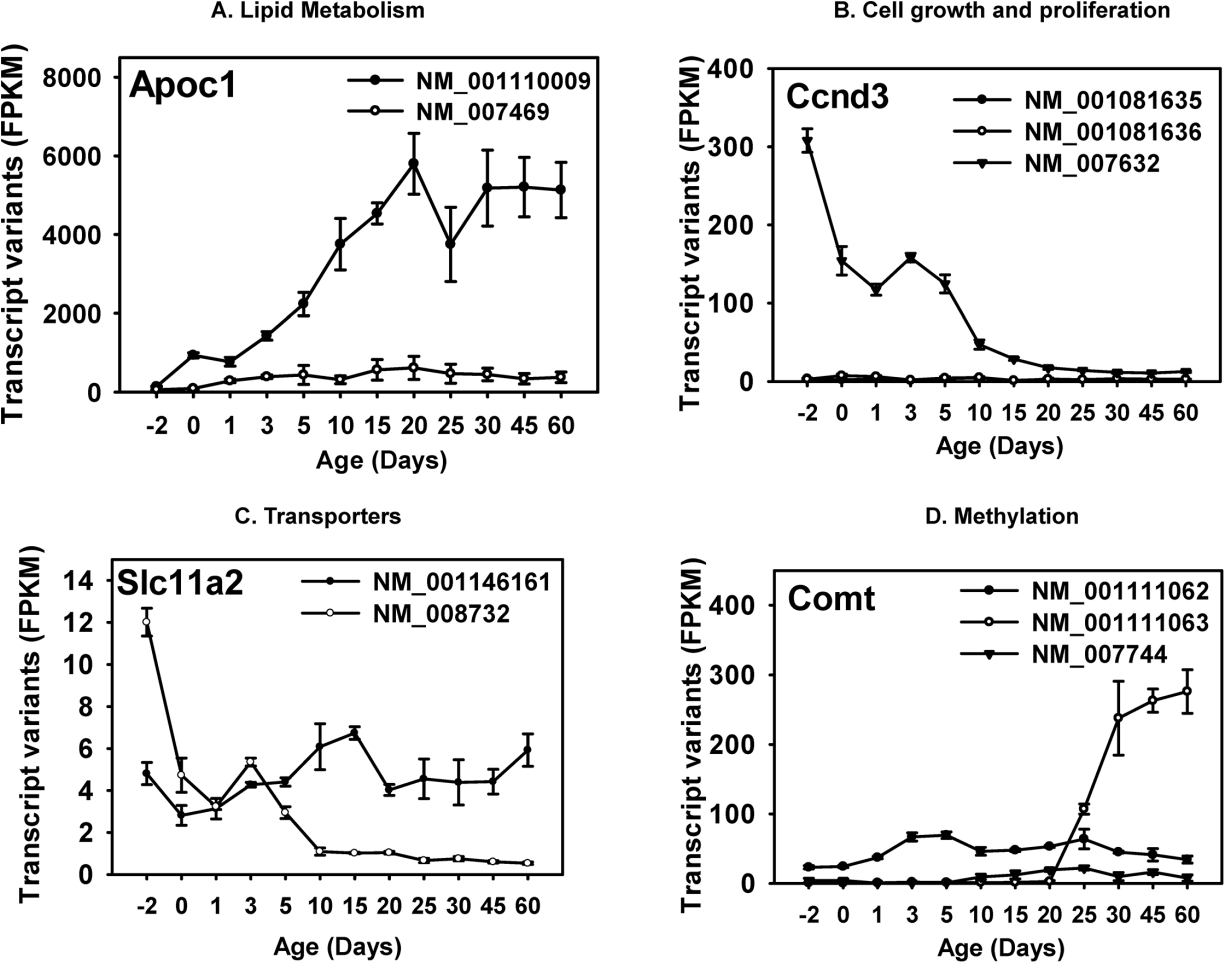
Selected examples of differential expression of known alternative splicing transcripts.

The 21,889 genes considered in this study represented 27,641 transcripts of which, 9,641 genes representing 10,696 transcripts were significantly expressed at the transcript level in at least one of the 12 analyzed ages **(S6 Table)**. Of these genes, 859 had multiple (>1) known splice variants that were significantly expressed at some point in development **(S7 Table)**. The relative expression levels of the splice variants of 90 of them were significantly different in at least one of the ages **(S4 Fig)**. Alternate transcripts of the same gene may differ in the 5’- or 3’- untranslated regions (UTRs), or in the coding exon structures. It is important to identify these differentially expressed transcripts, because 1) the regulation of protein translation requires cis-regulatory elements located mostly in the 5’- and 3’-UTRs, and trans-regulatory factors (such as RNA-binding proteins and microRNAs) which recognize specific RNA structures, thus modulating translation efficiency [51], and 2) differences in the coding exons may directly impact the amino acid sequences and modulate the protein localization or functions. In this study, we selected 4 genes as examples with known critical functions in the liver, and characterized their multiple differentially expressed transcripts during liver development. As shown in **(Fig 3)**, these genes fall into four critical cell-signaling pathways in the liver, namely lipid metabolism, cell growth and proliferation, transporters, and methylation.

In the lipid metabolism pathway, Apolipoprotein C1 (*Apoc1*) is a critical protein component of the *Apoc* family, which is primarly produced in the liver and is important for cholesterol metabolism [52]. As shown in **(Fig 3A)**, *Apoc1* transcript variant 1 (*NM_007469*), which is the longer transcript, was lowly expressed throughout liver development; conversely, *Apoc1* transcript variant 2 (*NM_001110009*), which has a shorter 5’-UTR, was the predominant transcript throughout liver development. It gradually increased in expression to adult levels from 2-days before birth to 20-days after birth and remained relatively stable thereafter. This suggests that the Apoc1 protein is likely produced mainly from variant 2. The lack of transcription of variant 1 may be due to low hepatic expression of the transcription factor that recognizes the alternative promoter of the longer transcript, and/or less permissive chromatin epigenetic environment at the alternative promoter region.

Regarding genes that are important for cell growth and/or proliferation **(Fig 3B)**, cyclin D3 (*Ccnd3*) is a D-type cyclin that governs the cell cycle machinery and progression through G(1) phase in response to extracellular signals. However, compared to the well-characterized Cyclin D1, *Ccnd3* is less frequently associated with cell malignancy [53]. All three known transcript variants (with variations in 5’-UTR) of *Ccnd3* were significantly expressed in liver in at least 1 of the 12 developmental ages. However, only variant 1 (*NM_007632*) was the major transcript throughout liver development, and it displayed a perinatal-enriched pattern. The longest transcript (variant 3, *NM_001081636*) and variant 2 (*NM_00181635*) were only minimally expressed in liver at all ages.

The proton-coupled divalent metal ion transporter *Slc11a2* (also called *Dmt1*) has 2 known transcript variants with both expressed during liver development **(Fig 3C)**. Variant 1 (*NM_001146161*) is the longer transcript encoding protein isoform 1. It contains of a stem-loop iron response element in the 3’-UTR. Varian 2 differs in both the 3’-coding region and 3’-UTR, leading to protein isoform 2 with a longer and distinct C-terminus without a stem-loop iron response element in the 3’-UTR. Before birth, variant 1 (*NM_001146161*) was the predominant transcript, however, in neonatal ages, both of the two variants were expressed at comparable levels. After 5-days of age, variant 1 remained the highly expressed transcript, whereas variant 2 (*NM_008732*) markedly decreased in liver.

The catechol-O-methyltransferase (*Comt*) is involved in the methylation of many chemicals and is a drug target for Parkinson’s disease [54, 55]. Despite its critical role in the central nervous system, *Comt* is predominantly produced from the liver (BioGPS) [56]. All three known transcript variants with alternate 5’-UTRs were expressed in liver; however, they displayed different ontogenic expression patterns **(Fig 3D)**. Variant 1 (*NM_001111062*, longest transcript) and 2 (*NM_007744*) were relatively stable throughout liver development, of which variant 1 was moderately higher than variant 2; in contrast, variant 3 (*NM_001111063*) was minimally expressed before 20-days of age, but was markedly increased and became the major transcript in adults.

### Fundamental gene expression patterns in mouse liver development

**Fig 4:**
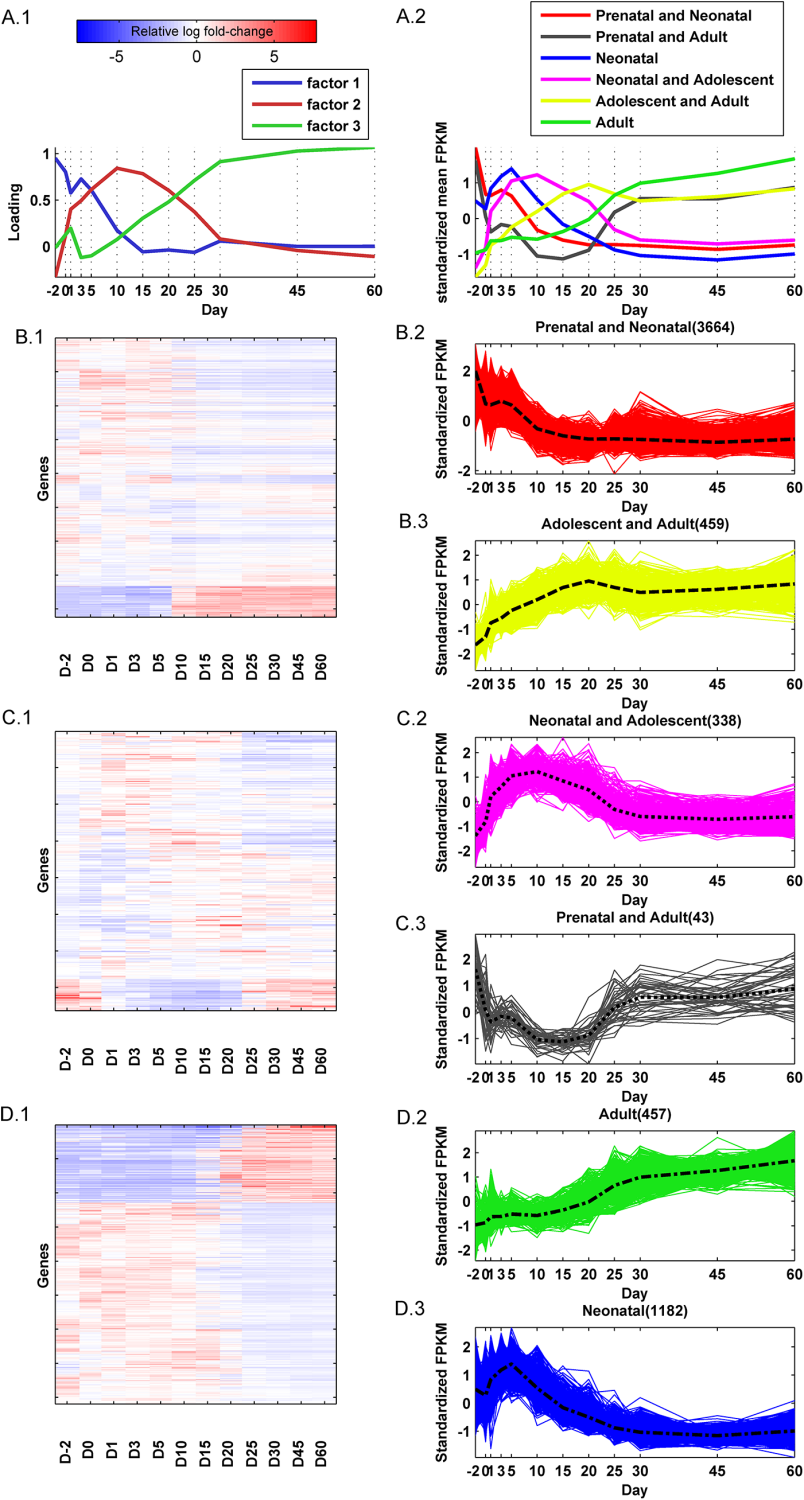
Fundamental gene expression patterns in mouse liver development. (A.1) Plot of the factor loadings from the exploratory factor analysis of the significantly differentially expressed genes at the 12 ages. Higher loadings indicate days with gene expression that is highly correlated with the factor. The patterns of the factor loading graphs describe the relative contribution of the factors to the observed gene expression over time. (A.2) The average temporal expression patterns of genes in the six clusters. (B.1) Expression heatmap of hierarchically clustered genes correlating with factor 1. (B.2) Standardized (zero mean and unit variance) gene expression pattern of genes positively correlated with factor 1. (B.3) Standardized gene expression pattern of the genes negatively correlated with factor 1. (C.1) Expression heatmap of hierarchically clustered genes correlating with factor 2. (C.2) Standardized (zero mean and unit variance) gene expression pattern of genes positively correlated with factor 2. (C.3) Standardized gene expression pattern of the genes negatively correlated with factor 2. (D.1) Expression heatmap of hierarchically clustered genes correlating with factor 3. (D.2) Standardized (zero mean and unit variance) gene expression pattern of genes positively correlated with factor 3. (D.3) Standardized gene expression pattern of the genes negatively correlated with factor 3. The perforated lines plot average expression. The color-bar represents the relative log fold-change of genes relative to the average expression over the 12 ages.

Factor analysis of the 7,289 *analysis-genes* attributed the observed variations of a majority of them to three latent factors, with 6,143 *analysis-genes* correlating, either positively or negatively, with at least one of the factors **(Fig 4A.1)**. These latent or unseen factors influencing global gene expression patterns could have different sources, including the effects of common upstream regulators. The loadings of these 3 factors plotted over time traced the underlying global gene expression patterns associated with these genes. The factor loading is a measure of the percentage of variation in global gene expression explained by the factor at a given age. These 6,143 *analysis-genes* were categorized into one of six distinct temporal expression patterns, based on their correlation with the factor loadings. The standardized mean expression patterns (gene expression was standardized across ages to have zero mean and unit standard deviation) of these six clusters are shown in **Fig 4A.2**.The clusters were labeled as, 'Prenatal and Neonatal', 'Prenatal and Adult', 'Neonatal', 'Neonatal and Adolescent', 'Adolescent and Adult', and 'Adult', reflecting the time period along the development in which activation of their genes were predominant.

Of the 6,143 genes, 4,123 were predominantly correlated with factor 1. Their hierarchical clustering is shown in **Fig 4B.1**. Of these 4,123 genes, 3,664 were positively correlated with the factor and 459 were negatively correlated with the factor **(Fig 4B.2 & 4B.3)**. Genes positively correlated with the first factor were highly expressed on Day -2, moderately expressed between Day 0 and 5 and decreased thereafter **(Fig 4B.2 red lines)**. This cluster of genes was labeled 'Prenatal and Neonatal'. Genes negatively correlated with factor 1 were highly suppressed during early development with a gradual increase in expression to be highly expressed from day 15 to adulthood **(Fig 4B.3 yellow lines)**. This cluster of genes was labeled 'Adolescent and Adult'.

There were 381 genes that predominantly correlated with factor 2. Their hierarchical clustering is shown in **Fig 4C.1**. Of these genes, 338 were positively correlated with the factor and 43 were negatively correlated with the factor. Genes positively correlated with factor 2 were more highly expressed between Day 1 and 20 **(Fig 4C.2 pink lines)**, and this cluster of genes was labeled 'Neonatal and Adolescent'. Genes negatively correlated with factor 2 were expressed during early (Day -2 to Day 1) and latter (Day 25 to 60) development and suppressed in-between **(Fig 4C.3 black lines)**, and therefore this cluster of genes was labeled 'Prenatal and Adult'.

There were 1,639 genes that predominantly correlated with factor 3. Their hierarchical clustering is shown in **Fig 4D.1**. Of these 1639 genes, 457 were positively correlated and 1182 were negatively correlated with the factor. Genes positively correlated with factor 3 increases after Day 20 **(Fig 4D.2, green lines)**. This cluster of genes was labeled 'Adult'. Genes negatively correlated with factor 3 are predominantly expressed between Day 1 and 10 **(Fig 4D.3, blue lines)**, and therefore this cluster of genes was labeled 'Neonatal'.

A majority of genes in all 6 groups was comprised of enzymes (~11.6% - 23.9%) followed by transporters (~6.2% - 9.3%) and transcription regulators (~ 5.6% - 8.5%) **(S8 Table)**. Interestingly, the proportion of the different protein types within each group did not differ significantly between groups (Chi-square test of independence of development period and protein type *p-value* = 0.98). A majority of genes in all 6 groups were localized in the cytoplasm (~35.7% - 40.8%), followed by the nucleus (~13.9% - 25.0%) and plasma membrane (~10.6% - 12.4%) **(S9 Table)**.

### Functional analysis of gene clusters

**Fig 5:**
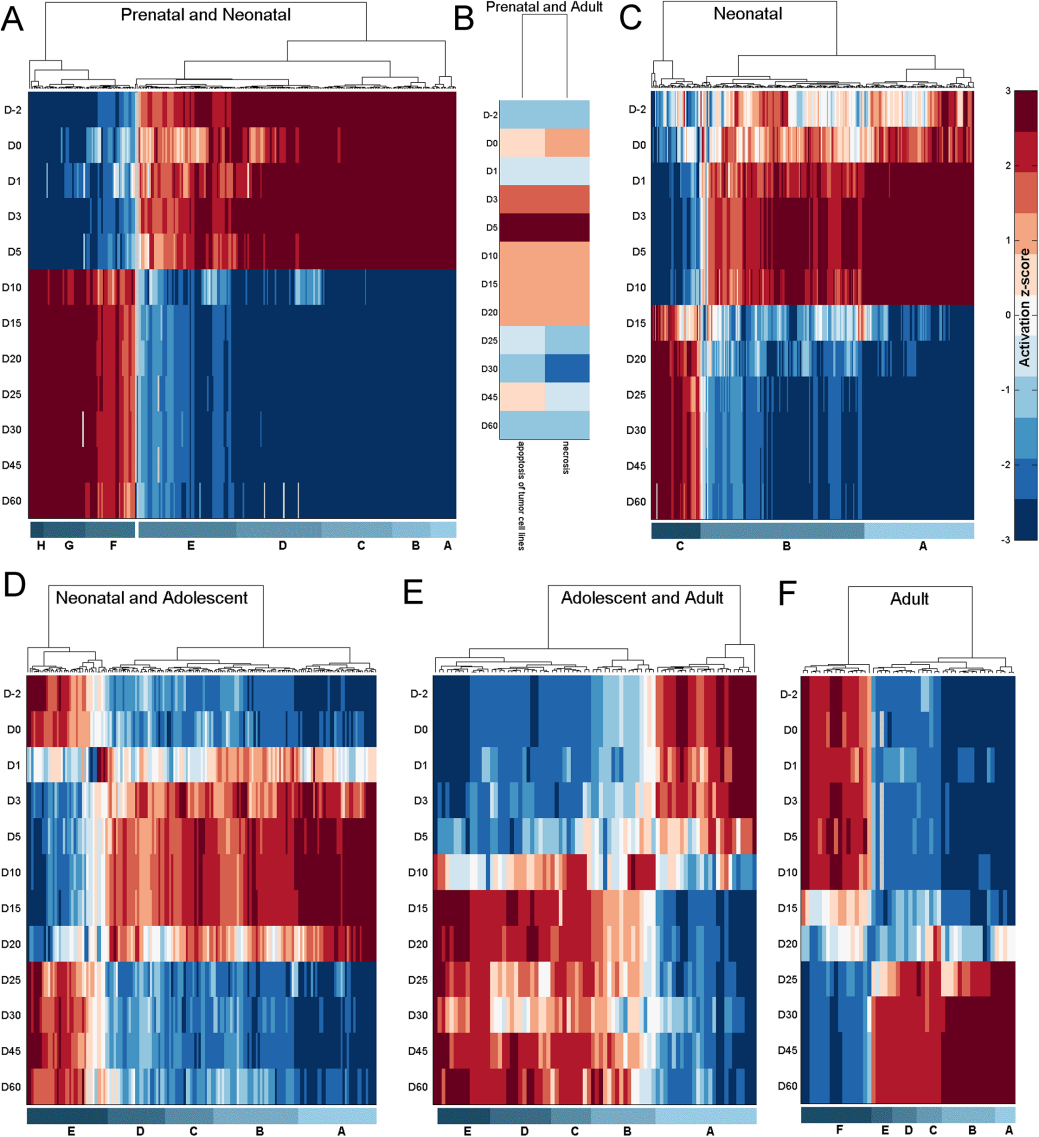
Functional analysis of gene clusters. The activation or suppression status of different biological functions associated with genes in (A) Perinatal and Neonatal, (B) Prenatal and Adult, (C) Neonatal, (D) Neonatal and Adolescent, (E) Adolescent and Adult, and (F) Adult groups respectively. Red represents activation and blue represents repression of a biological function. The individual biological functions represented in the labeled sub-clusters are listed in S7 to S11 **Tables**.

The efficacy of biological functions associated with genes in the six clusters mentioned above was quantified at each age by the activation z-score (Ingenuity Systems, www.ingenuity.com) [57], a measure of the level of activation or inhibition of a biological function associated with a set of differentially expressed genes. A differential expression was calculated for each gene at each age by dividing its expression by the average expression of the gene over the 12 ages. Biological functions with a significant activation z-score (absolute value greater than or equal to 2) in at least one age were hierarchically clustered (distance measure: Euclidean; linkage function: Ward) **(Fig 5)**.

Genes in the 'Prenatal and Neonatal' group **(Fig 5A)** are associated with several biological functions (see **S10 Table** for the complete list) that are mainly activated (sub-clusters A—E), but also suppressed (sub-clusters F—H) during this period. Functions of sub-cluster A are associated with cell death and survival. Functions of sub-cluster B are associated with cellular movement in the development of the hematological system, such as movement of neutrophils, homing of neutrophils, chemotaxis of neutrophils, and movement of granulocytes. Genes in this cluster are also associated with cellular maintenance functions such as engulfment of cells, endocytosis, and phagocytosis. They also play a part in the assembly and organization of the cell such as organization of cytoplasm, organization of cytoskeleton, and microtubule dynamics. Genes in sub-cluster C are associated with functions relating to the development and functioning of the hematological system, including phagocytosis of myeloid cells, blood cells, leukocytes, and phagocytes. Other biological functions associated with this sub-cluster of genes include growth of connective tissue and organism, metabolism of protein, free radical scavenging, and cell cycle related functions. Genes in sub-cluster D are associated with cellular growth and proliferation related functions, such as proliferation of blood cells, fibroblasts, immune cells, mononuclear leukocytes, and lymphocytes. These genes are also associated with biological functions related to cell cycle, cell morphology, cell-to-cell signaling and interaction, cellular assembly and organization, and molecular transport. Among the functions related to genes in sub-cluster E include cell cycle, cell death and survival, cell morphology, cellular assembly and organization, cellular growth and proliferation, molecular transport, and protein synthesis. Genes in sub-clusters F, G and H are associated with biological functions suppressed during early development and include several hematological disease related functions such as hemolysis, hemolytic anemia, blood protein disorder, cytopenia, spherocytosis, hematological neoplasia, malignant hematopoietic neoplasm, lymphomagenesis, anisocytosis, incidence of lymphoma, and anemia.

The 43 genes in the 'Prenatal and Adult' cluster were significantly associated with two cell death and survival related functions, necrosis and apoptosis of tumor cell lines **(Fig 5B)**. These biological functions were suppressed during the early (day -2 to day 1) and later (day 25 to day 60) period of development and expressed during the intermediary period (day 3 to day 20). This is in contrast to the expression pattern of these genes which are expressed during the early and later period of development and suppressed in-between.

Biological functions associated with genes in the 'Neonatal' group **(Fig 5C)** are mainly activated (sub-clusters A-B) but also suppressed (sub-cluster C) during day 1 to day 10 of development **(S11 Table)**. Genes in sub-cluster A are associated with biological functions relating to cell movement, cell death and survival, cell-to-cell signaling and interaction, cellular assembly and organization, cellular development, cellular function and maintenance, and cellular growth and proliferation. Genes in sub-cluster B are also associated with many of these functions along with other biological functions relating to cell cycle, cell death and survival, cell morphology, cell-mediated immune response, organ development, and tissue development. Biological functions in sub-cluster C are suppressed earlier in development. They include functions relating to disease states such as cancer (lymphohematopoietic cancer, hyperplasia, and malignant hematopoietic neoplasm), growth failure, abnormal morphology, fibrosis, and bacterial infection.

Biological functions associated with the 'Neonatal and Adolescent' cluster of genes **(Fig 5D)** are mainly activated from around day 1 to day 20 of development (sub-clusters A-D), but are also suppressed in some cases (sub-cluster E) during this period **(S12 Table)**. Genes in sub cluster A are associated with biological functions relating to cellular movement (e.g. expansion, proliferation, homing, chemotaxis, migration), lipid metabolism (e.g. transport of lipid, fatty acid metabolism, synthesis of lipid, cleavage of lipid, hydrolysis of lipid, hydrolysis of phospholipid, metabolism of terpenoid, steroid metabolism, oxidation of lipid), and carbohydrate metabolism (e.g. hydrolysis, cleavage). Genes in sub-clusters B-D relate to biological functions such as carbohydrate metabolism (e.g. metabolism of carbohydrate, synthesis of carbohydrate, metabolism of hexose, metabolism of D-glucose), tissue development (e.g. adhesion of vascular endothelial cells, proliferation of smooth muscle cells, fibrogenesis, formation of filaments, proliferation of hepatocytes, proliferation of connective tissue cells, growth of connective tissue, adhesion of endothelial cells, proliferation of epithelial cells), lipid metabolism (e.g. oxidation of fatty acid, oxidation of long chain fatty acids, uptake of lipid, clearance of lipid, removal of lipid, efflux of cholesterol, uptake of fatty acid, esterification of lipid, metabolism of membrane lipid derivative, release of phospholipid, accumulation of lipid, conversion of lipid, metabolism of triacylglycerol), metabolism of proteins, vitamins and minerals, and molecular transport (e.g. transport of carboxylic acid, release of metal). Biological functions associated with sub-cluster E are active during the early (day -2 to day 0) and latter (day 25 to day 60) period of development and relatively suppressed in the in-between period. Biological functions in this sub-cluster are associated with several disease functions, such as disorder of lipid metabolism, disorder of glucose metabolism, hepatic steatosis, insulin resistance, and thrombosis.

A majority of biological functions associated with the 'Adolescent and Adult' group of genes **(Fig 5E)** tend to be activated from day 10 to day 60 (sub-clusters B-E). However, a sub-cluster of biological functions (sub-cluster A) is active during the early part of development (day -2 to day 5) and suppressed thereafter **(S13 Table)**. There are several metabolism related functions associated with sub-clusters B-E, including the metabolism of terpenoid, nucleotide, eicosanoid, amino acids, vitamins, and retinoids. Other biological functions associated with these groups include, concentration of levothyroxine, synthesis of terpenoid, transport of cholesterol, secretion of lipid, release of prostaglandin, transport of steroid, and efflux of lipid and cholesterol. Biological functions associated with sub-cluster A include quantity of enzymes, quantity of ammonia in blood, quantity of aspartate transaminase in blood, quantity of glutamic-pyruvate transaminase (*GPT*) in blood, oxidative stress, hepatic steatosis, inflammation of liver, necrosis of liver, and weight loss.

There are two main sub-clusters of biological functions associated with the 'Adult' cluster of genes **(Fig 5F, S14 Table)**. One is highly active during the latter period of development (day 25 to day 60, sub-clusters A-E) and another is primarily active during the early period of development (day -2 to day 15, sub-cluster F). Biological functions associated with sub-cluster B relate to lipid metabolism (e.g. uptake of lipids, transport of lipids, synthesis of lipids, fatty acid metabolism, hydroxylation of lipids, modification of retinaldehyde, metabolism of terpenoids, metabolism of retinoids), vitamin and mineral metabolism, drug metabolism, glucuronidation of hormones, and transport of carboxylic acids. Biological functions in sub-cluster C include carbohydrate metabolism, transport of carbohydrates, and nucleic acid metabolism. The biological functions in sub-cluster D are synthesis of tretinoin, conjugation of 12-hydroxyeicosatetraenoic acid, conversion of hormones, conversion of lipids, conjugation of lipids, removal of lipids, glucuronidation of lipids, glucuronidation of estrogen, transport of steroids, and synthesis of terpenoids. Biological functions in sub-cluster E include conversion of hormones, conversion of lipids, removal of lipids, transport of steroids, and synthesis of terpenoids. Sub-cluster F which is expressed in early but suppressed in latter periods of development include biological functions relating to cell death and survival, protein synthesis, quantity of ketone bodies, and disease functions, such as hepatocellular carcinoma, necrosis of liver, inflammation of liver, dysfunction of mitochondria, and fibrosis. Sub-cluster A represents a set of biological functions associated with infectious diseases that is highly active in the adult. Like the disease functions associated with sub-cluster A in the 'Prenatal and Neonatal' group of genes, these functions relate to HIV infection. It also includes a disease function relating to the infection of tumor cell lines.

In summary, we could see that some bio-functions are associated with a particular age group while others are associated with multiple age groups. For example, functions such as RNA damage and repair and RNA trafficking are only associated with the 'Prenatal and Neonatal' group while functions such as hematopoiesis, humoral immune response, cell morphology and protein trafficking, are associated with both the 'Prenatal and Neonatal' and the 'Neonatal' groups of genes. Bio functions relating to drug metabolism are associated with genes in the 'Adolescent and Adult' and 'Adult' groups. Bio functions relating to the hepatic system development and function, lipid metabolism, and, vitamin and mineral metabolism, are associated with genes in the 'Neonatal and Adolescent', 'Adolescent and Adult', and 'Adult' age groups. Activity of bio functions relating to cell signaling, cell-mediated immune response, and developmental disorder, is seen in the 'Neonatal' and 'Neonatal and Adolescent' age groups. Bio functions associated with organismal survival are active from the 'Neonatal' ages through to the 'Adolescent and Adult' ages while bio functions associated with organ development, and organismal injury and abnormalities, are active from the 'Neonatal' ages through to 'Adult'. Bio functions relating to cell cycle, cellular assembly and organization, connective tissue development and function, organ morphology, and, tissue morphology, are active during the early period of development including the age gropes from 'Prenatal and Neonatal' through to 'Neonatal and Adolescent'. Bio functions relating to cell death and survival, inflammatory response, molecular transport, protein synthesis, and, small molecule biochemistry, are associated with all age groups.

### Upstream regulator analysis

**Fig 6:**
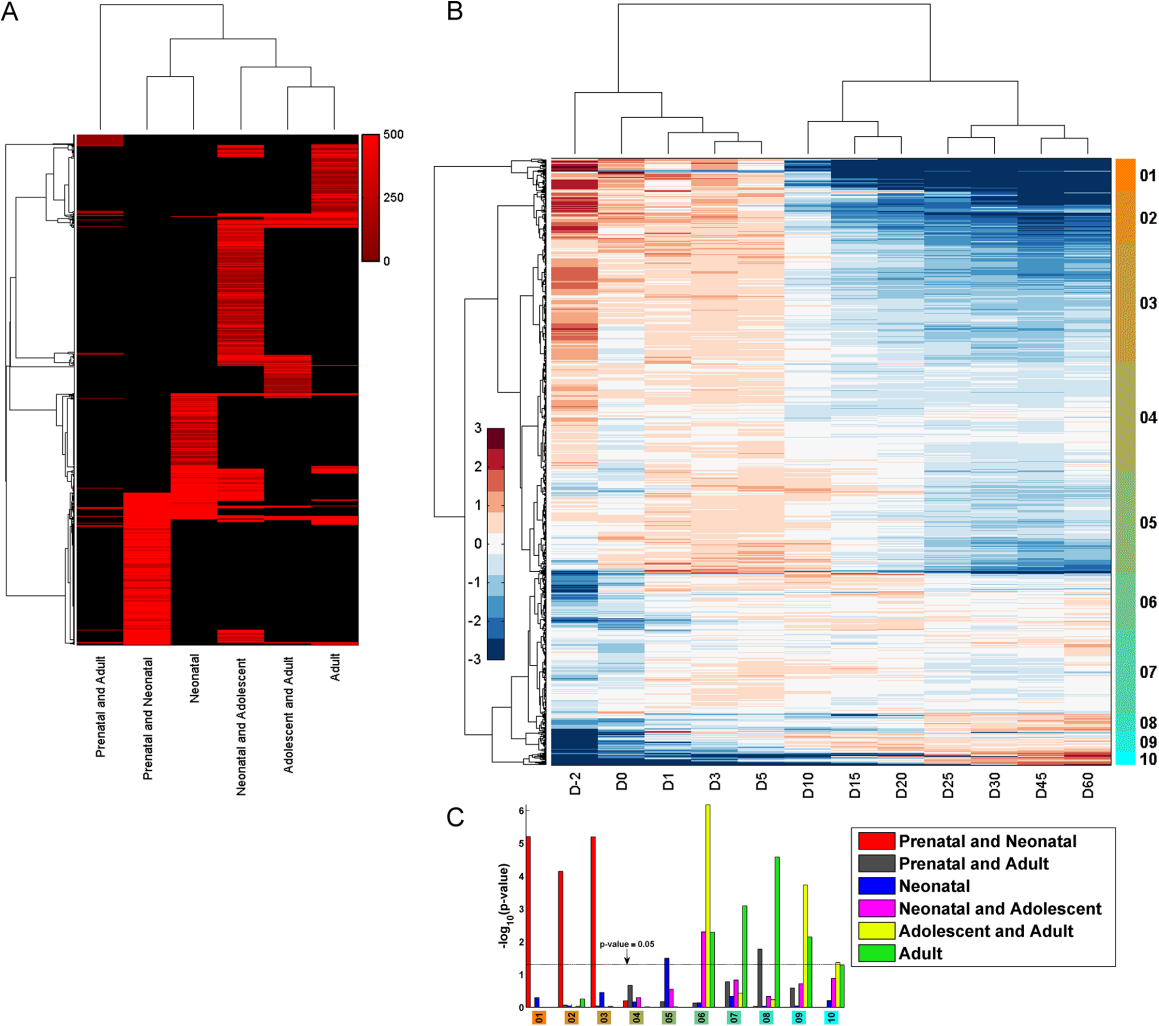
Upstream regulator analysis. (A) Heatmap representing the number of genes targeted by each of the upstream regulators in the six temporal groups. Upstream regulators (rows) are ordered according to the hierarchical clustering (distance measure: correlation, linkage function: average) of the hit count matrix of the number of target genes. The red intensity is proportional to the number of targets. (B) Heatmap showing the temporal expression patterns of the hierarchically clustered upstream regulators (distance measure: Euclidean, linkage function: Ward). (C) Bar graph showing the hypergeometric *p-value* of the significance of association of upstream regulators in each sub-cluster in B with genes in each of the six temporal groups.

Identifying key upstream regulators responsible for the observed changes in gene expression in development is a vital part in understanding liver development. In this regard, 1,031 upstream regulators with significant expression in at least one age and significantly associated (right tailed Fisher’s exact test *p-value* less than or equal to 0.05 calculated on the overlap between genes in the group and genes targeted by the upstream regulator) with genes responsible for the observed expression patterns were identified (using IPA; *www*.*ingenuity*.*com*, **Fig 6A & 6B; S15 Table**; IPA provides a curated database of upstream regulators and their target genes). Of these upstream regulators, 307, 52, 255, 441, 133, and 216 were associated with the Prenatal and Neonatal, Prenatal and Adult, Neonatal, Neonatal and Adolescent, Adolescent and Adult, and Adult groups, respectively. Upstream regulators associated with the Adolescent and Adult, and Adult groups showed a very significant overlap (hypergeometric *p-value* 6.1e-05). Upstream regulators associated with the Prenatal and Adult, and Adult groups also showed a significant overlap (hypergeometric *p-value* 0.03) **(Fig 6A; Table 1)**. Apart from that, there wasn’t a statistically significant overlap in the upstream regulators targeting genes in the various temporal groups **(Table 1)**. Fig 6A outlines the hits (genes) **(S15 Table)** for each upstream regulator in the six temporal groups. Table 1 and Fig 6A suggests that a majority of the upstream regulators target genes in a specific temporal group rather than across multiple groups. As expected, the hierarchical clustering of the groups in Fig 6A suggests a tighter correlation between upstream regulators in adjacent temporal groups as opposed to distant ones.

**Table 1:** Overlap of upstream regulators between developmental periods.

		*Number of upstream regulators*				
*Period-1*	*Period-2*	*Total*	*Period-1 ∩ Period-2*	*Period-1*	*Period-2*	*Hypergeometric test p-value*
*Adolescent and Adult*	*Adult*	*1031*	*46*	*133*	*216*	*6*.*18E-05*
*Prenatal and Adult*	*Adult*	*1031*	*17*	*52*	*216*	*0*.*029172*
*Prenatal and Adult*	*Adolescent and Adult*	*1031*	*10*	*52*	*133*	*0*.*12046*
*Neonatal and Adolescent*	*Adolescent and Adult*	*1031*	*63*	*441*	*133*	*0*.*14609*
*Prenatal and Neonatal*	*Prenatal and Adult*	*1031*	*14*	*307*	*52*	*0*.*72738*
*Prenatal and Adult*	*Neonatal and Adolescent*	*1031*	*18*	*52*	*441*	*0*.*91488*
*Prenatal and Adult*	*Neonatal*	*1031*	*8*	*52*	*255*	*0*.*96701*
*Neonatal and Adolescent*	*Adult*	*1031*	*79*	*441*	*216*	*0*.*98457*
*Neonatal*	*Adolescent and Adult*	*1031*	*22*	*255*	*133*	*0*.*99443*
*Prenatal and Neonatal*	*Neonatal*	*1031*	*53*	*307*	*255*	*0*.*99992*
*Neonatal*	*Neonatal and Adolescent*	*1031*	*84*	*255*	*441*	*0*.*99992*
*Neonatal*	*Adult*	*1031*	*32*	*255*	*216*	*0*.*99997*
*Prenatal and Neonatal*	*Neonatal and Adolescent*	*1031*	*60*	*307*	*441*	*1*
*Prenatal and Neonatal*	*Adolescent and Adult*	*1031*	*17*	*307*	*133*	*1*
*Prenatal and Neonatal*	*Adult*	*1031*	*28*	*307*	*216*	*1*

The expression pattern of the 1,031 upstream regulators is shown in Fig 6B. As expected, the temporal ordering is preserved in the hierarchical clustering of the expression distribution of the upstream regulators (clustering of columns) over the 12 ages with two main clusters separating the early period of development (Day -2 to Day 5) and the latter period of development (Day 10 to Day 60). The hierarchical clustering of the temporal expression patterns of the upstream regulators (clustering of rows) distinguishes two main clusters. The first cluster, consisting of sub-clusters 01 to 05, comprises upstream regulators whose relative expression is high during the early period of development. The second cluster, consisting of sub-clusters 06 to 10, comprises upstream regulators whose relative expression is high during the mid to latter part of development. **Fig 6C (S16 Table)** shows the hypergeometric *p-value* of the significance of association of upstream regulators in each sub-cluster in Fig 6B with genes in each of the six temporal groups. Upstream regulators in sub-clusters 01–03 are highly expressed in the early period of development, in particular Day -2, and are significantly associated with genes in the Prenatal and Neonatal group (hypergeometric *p-value* 7.2E-05 ~ 6.2E-06). Upstream regulators in sub-cluster 05, which are expressed between Day 0 and Day 10, are significantly associated with genes in the Neonatal group (hypergeometric *p-value* 3.2E-02). Upstream regulators in sub-cluster 06 are associated very significantly with genes in the Adolescent and Adult group (hypergeometric *p-value* 6.6E-07), but are also significantly associated with genes in the Neonatal and Adolescent group (*p-value* 5.0E-03) and the Adult group (*p-value* 5.1E-03). A majority of upstream regulators in sub-cluster 07 are expressed between Day 1 and Day 20 but are significantly associated with genes in the Adult group (*p-value* 8.0E-04). Upstream regulators in sub-cluster 08 are expressed during the latter part of development (Day 25 –Day 60) and are significantly associated with genes in the Adult, and Prenatal and Adult groups of genes. The relative expression of upstream regulators in sub-cluster 09 are up from Day 10 to Day 60, and are significantly associated with genes in the Adolescent and Adult group (*p-value* 1.9E-04) and also the Adult group (*p-value* 7.1E-03). Upstream regulators in sub-cluster 10 are highly expressed during the latter period of development (Day 25 –Day 60) and are significantly associated with genes in the Adolescent and Adult group (*p-value* 4.3E-02).

**Fig 7:**
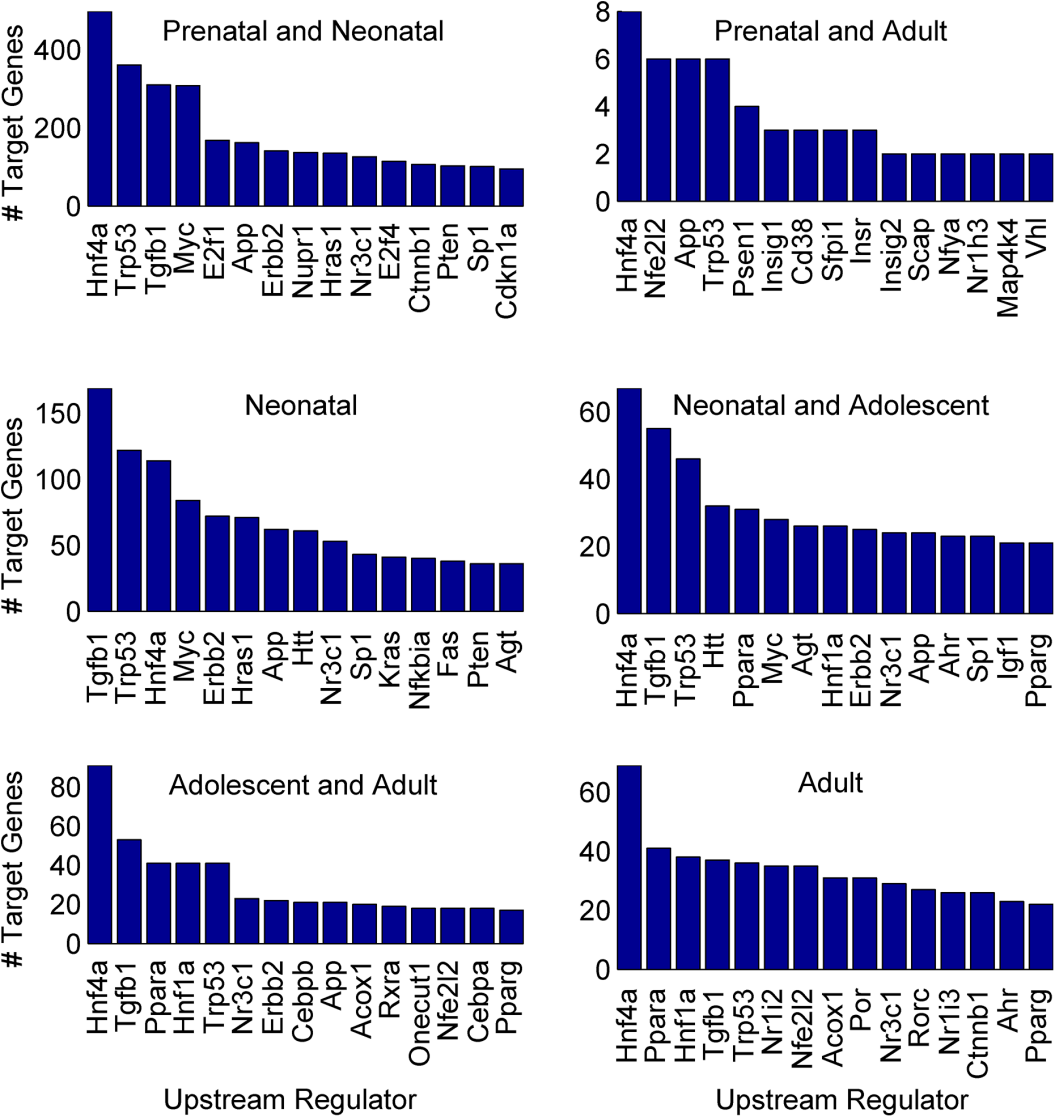
Top 15 upstream regulators with the highest number of target genes. The top 15 upstream regulators with the highest number of target genes in the Prenatal and Neonatal, Prenatal and Adult, Neonatal, Neonatal and Adolescent, Adolescent and Adult, and Adult groups respectively.

A majority of the 1,031 upstream regulators were transcription regulators (~ 19% - 33% in each group) followed by enzymes (~ 16% - 32% in each group) and kinases (~ 5% - 13% in each group) **(S17 Table)**. They were directly associated with 3,440 *analysis-genes* of which 2,022, 24, 638, 229, 266, and 261 were in the Prenatal and Neonatal, Prenatal and Adult, Neonatal, Neonatal and Adolescent, Adolescent and Adult, and Adult groups, respectively **(S15 Table)**. The top 15 upstream regulators with the highest number of target genes in each group are shown in **Fig 7**. The two transcription regulators, *Hnf4α* and *Trp53* were associated with a substantial number of genes in all six groups **(Fig 7)**. The upstream regulators, *Tgfb1*, *Erbb2*, *Jun*, *Nr3c1*, *Apoe*, *and Smarcb1* were associated with multiple target genes in all groups except the Prenatal and Adult group **(S15 Table)**. *Hnf4α* was the most prodigious upstream regulator in the number of associated target genes in all the groups except for the Neonatal group, in which it was third. *Hnf4α* was associated with 498, 8, 114, 67, 91 and 69 genes in the Prenatal and Neonatal, Prenatal and Adult, Neonatal, Neonatal and Adolescent, Adolescent and Adult, and Adult groups respectively. Several genes were also associated with multiple upstream regulators **(S5 Fig)**. For example, in the Prenatal and Neonatal group, *Myc* had the highest number of associated upstream regulators (121) followed by *Trp53* (94) and *Cdkn1b* (76). For the Prenatal and Adult group, *Fth1* had 14 associated upstream regulators followed by *Fdps* (13) and *Gpam* (12). In the Neonatal group, *Icam1* had 68 upstream regulators associated with it, followed by *Fn1* (66) and *Acta2* (65). In the Neonatal and Adolescent group, *Ccnd1* had 182 upstream regulators associated with it followed by *Pparg* (102) and *Mmp2* (96). In the Adolescent and Adult group, *Cyp7a1* had 31 upstream regulators associated with it followed by *Apoe* (26) and *Gpt* (21). In the Adult group, *Scd1* had 39 upstream regulators associated with it followed by *Igf1* (38) and *Egfr* (35).

### Identifying upstream regulatory modules

Many upstream regulators function in modules co-regulating clusters of genes [58, 59]. Identifying these regulatory modules and their target genes provides invaluable insights into the molecular dynamics of liver development. An iterative clustering algorithm **(see Methods)** was used to identify several putative upstream regulatory modules that potentially co-regulate sets of downstream genes in the six temporal groups. The algorithm modularizes upstream regulators with correlated (negative or positive) expression patterns, whose individual regulators target a cluster of downstream genes. A list of putative regulatory modules and their target genes are given in **S18 to S23 Tables.** The identified regulatory modules vary between 2 and 7 in the number of upstream regulators, which co-regulate gene clusters varying in size between 3 and 106 genes. For example, the transcription regulator *Trp53*, and the growth factor *Tgfb1*, together regulate 105, 46, 23, 10 and 8 downstream targets in the Prenatal and Neonatal, Neonatal, Neonatal and Adolescent, Adolescent and Adult, and Adult groups respectively (**S18, S20 to S23 Tables)**. These genes were primarily associated with biological functions relating to cell death and survival, cellular growth and proliferation, and cell cycle (Ingenuity pathway analysis). Some regulatory modules comprise of regulators that have anti-correlated expression patterns. For example, in the Prenatal and Neonatal group, the upstream regulators, *Trp53* and *Igf1* co-regulate 43 genes (**S18 Table)** and have opposing expression patterns, while the relative expression of *Trp53* decreases the relative expression of *Igf1* increases over time. Several such modules with anti-correlated regulators were identified, including 178 in the Prenatal and Neonatal group, 63 in the Neonatal group, 56 in the Neonatal and Adolescent group, 7 in the Adolescent and Adult group, and 14 in the Adult group (**S18, S20 to S23 Tables; modules with negative ICC)**. Some other examples of the identified regulatory modules include the regulator module comprising of two genes, *Nfe2l2 and Sfpi1* in the Prenatal and Adult group that regulates a cluster of three genes responsible for acid phosphatase activity (**S19 Table)**. The regulatory module comprising the three genes *Foxa2*, *Ppara*, *and Pparg* in the Neonatal and Adolescence group regulates a cluster of 8 genes associated with the positive regulation of cholesterol esterification (**S21 Table)**. The module comprising of the four genes *Hnf4α*, *Ppara*, *Pparg*, *and Rxra* in the Adolescence and Adult group regulate a cluster of 5 genes associated with lipid transporter activity and cholesterol transporter activity (**S22 Table)**. The module comprising of the three genes *Hnf4α*, *Nr1i2 (PXR)*, *and Nr1i3 (CAR)* regulates a cluster of 8 genes in the Adult group that are responsible for bile acid-exporting ATPase activity, acyl-CoA oxidase activity, caffeine oxidase activity, and aromatase activity among other things (**S23 Table)**.

### Novel Isoform Analysis

Alternative splicing is one of the fundamental mechanisms by which genes achieve diversity in their protein products. It has being determined that over 60% of human and mouse genes are alternatively spliced [60, 61], however many of these splice variants are still to be identified. In the present study, 65,267 candidate novel isoforms (Cufflinks class code j) [62] were detected, out of which 2,383 were significantly expressed in at least one of the sampled ages. These novel isoforms were further filtered down to 1,455 **(S24 Table)** by leveraging expression information distributed over time to reflect the correlated nature of time series expression data. In essence, it is expected in this selection criterion for truly expressed novel isoforms to have a coordinated expression pattern over time, as opposed to random fluctuation **(see Methods)**. Several genes with novel isoforms were detected in the six temporal groups including 646 (17.7%), 13 (30.2%), 128 (10.8%), 103 (30.5%), 172 (37.4%) and 77 (16.8%) in the Prenatal and Neonatal, Prenatal and Adult, Neonatal, Neonatal and Adolescent, Adolescent and Adult, and Adult groups, respectively **(S24 Table)**. All common types of alternative splicing events, such as exon skipping, intron retention, alternative exons, cassette exons, alternative transcript start, and alternative transcript termination were detected **(S25 Table)**.

**Fig 8:**
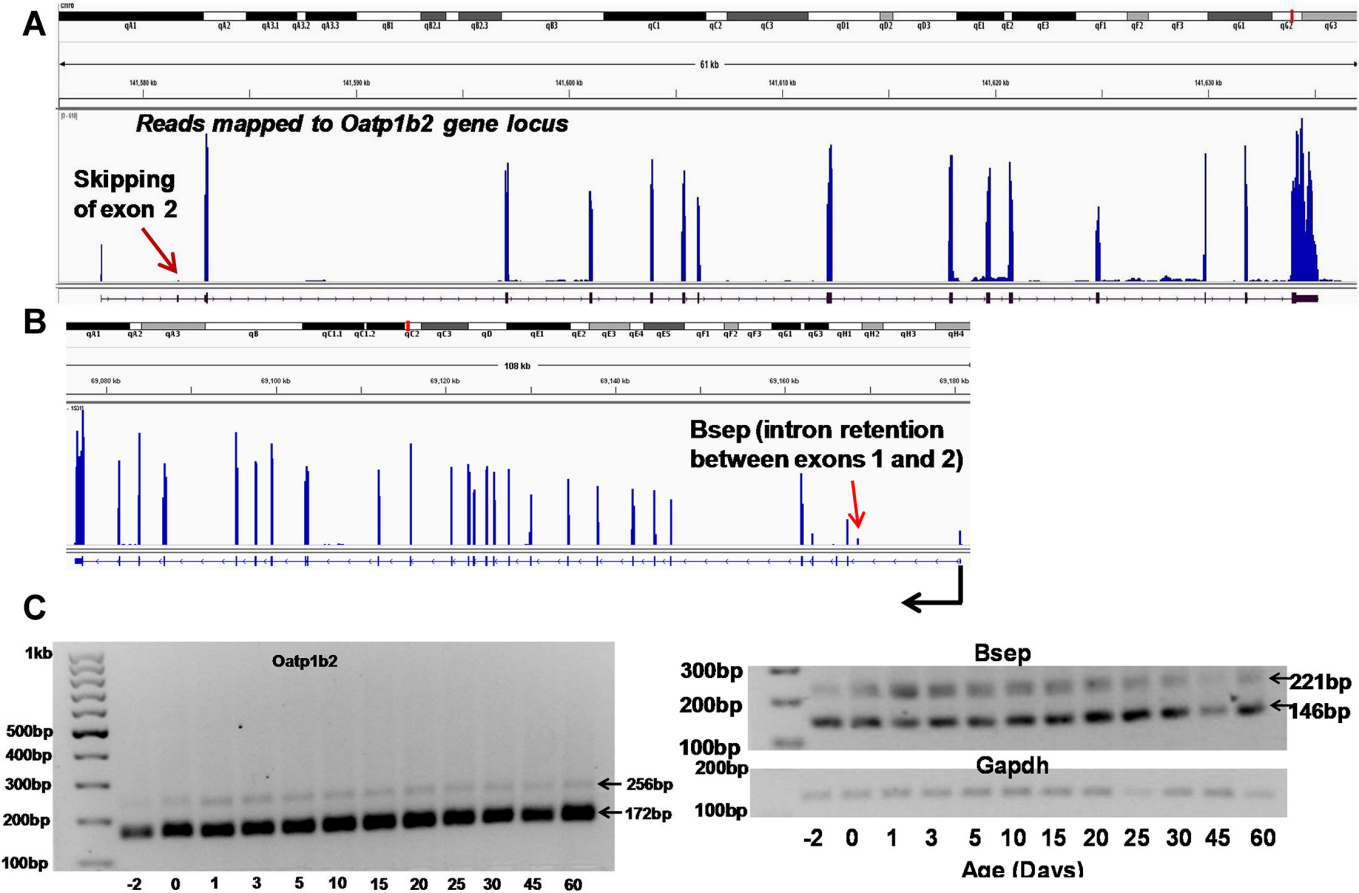
Validation of novel transcript variants. (A) Skipping exon 2 in *Oatp1b2* (B) Intron retention between exons 1 and 2 in *Bsep*. Data are visualized by the IGV (Broad Institute). Reads mapped to the gene from a day.2 liver are shown in the examples. (C) A semiquantitative PCR gel electrophoresis of the two Oatp1b2 isoforms and two Bsep isoforms along with Gapdh.

We validated the novel transcript variants of *Slco1b2* (*Oatp1b2*) and *Abcb11* (*Bsep*). The organic anion transporting polypeptide *(Oatp) 1b2* is a liver-specific basolateral uptake transporter for various xenobiotics and unconjugated bile acids [63]; the bile salt export pump (*Bsep*) is the rate-limiting bile acid canalicular efflux transporter in liver [64]. RNA-Seq identified a novel exon 2 skipping pattern in *Oatp1b2* 5’-UTR, as well as a novel intron 1 retention pattern in *Bsep* 5’-UTR **(Fig 8A & 8B)**. Interestingly, end-point PCR gel electrophoresis demonstrated that the novel transcript variant of *Oatp1b2* without exon 2 (which is 84bp shorter) was in fact the major transcript variant throughout liver development, whereas the known transcript was only expressed at minimal levels **(Fig 8C)**. The intron retention of *Bsep* was also confirmed by end-point PCR gel electrophoresis, however, its expression is lower compared to the known transcript that does not have partial intron 1.

**Fig 9:**
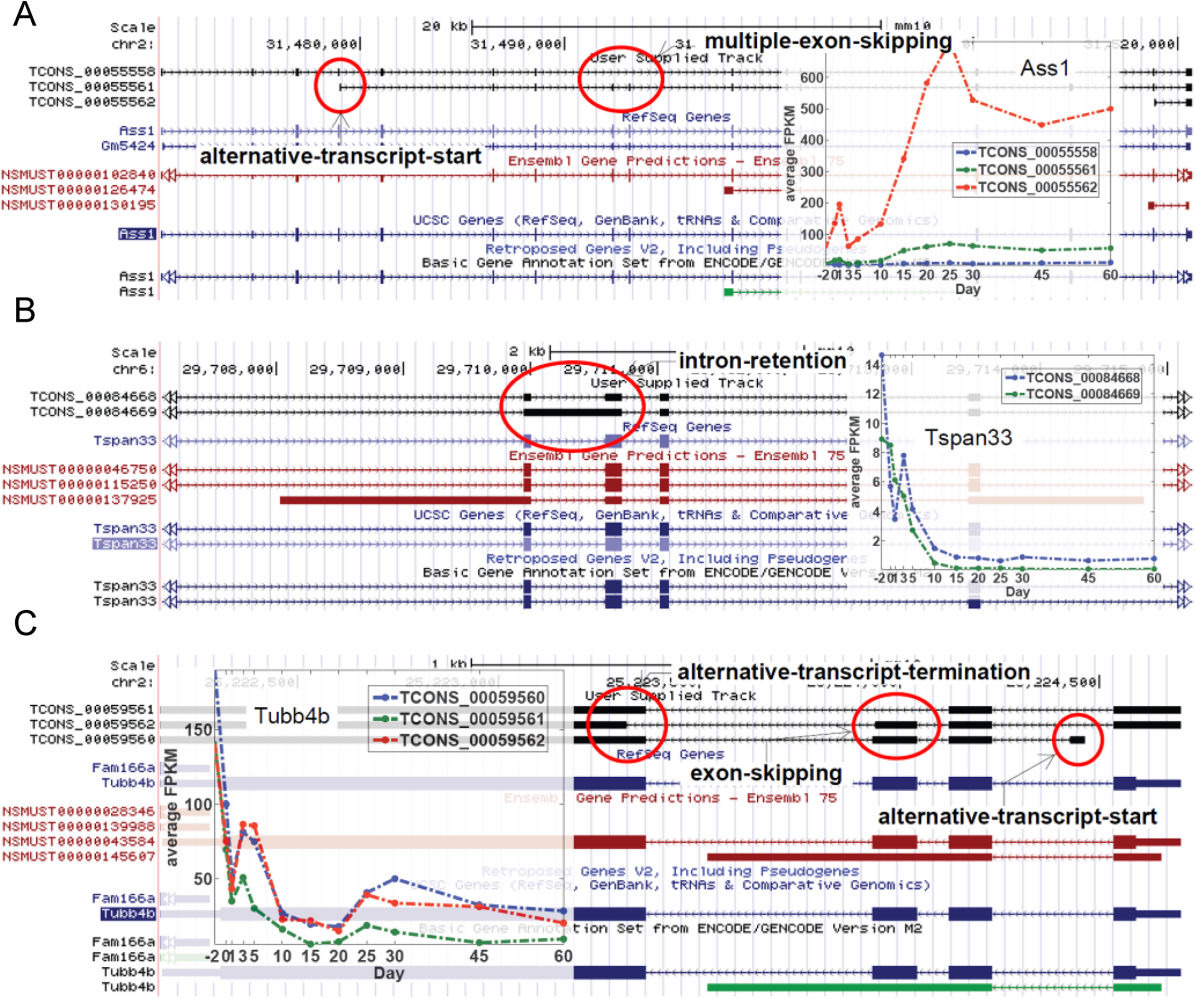
Examples of the different novel isoform variants. (A-C) Examples of novel isoforms from the selected genes. The novel isoform track is the first track on top in black. This is followed by the RefSeq Genes track (light blue), the Ensembl Genes track (red) and UCSC Genes track (dark blue). The expression pattern of the novel isoforms over the 12 ages is shown in the embedded graphs. The different features constituting the novel isoforms are highlighted by red circles and labeled as appropriate. Exons are shown as boxes. Arrows indicate the direction of transcription.

A few other examples of the different novel isoform variants are shown in **Fig 9**. The gene *Ass1* (argininosuccinate synthase) has novel isoforms with multiple skipped exons **(Fig 9A)**. The gene *Tspan33* (tetraspanin 33) has a novel isoform with an intron-retention between exons 2 and 3 (**Fig 9B**, *TCONS_00084668*). However the expression levels of both the known and novel isoforms stay similar with time starting at high levels and rapidly reducing until Day 10 and continuing at a reduced level thereafter. The gene *Tubb4b* (tubulin, beta 4B class IVb) is a gene whose expression pattern does not fall into any one of the defined age groups. This gene has three novel isoforms with significant expression mainly during early development **(Fig 9C)**. One of its isoforms has an alternative start site significantly downstream from the canonical start site (*TCONS_00059560*), another has a shorter alternative termination site (*TCONS_00059562*) and the third novel isoform has a skipped third exon (*TCONS_00059561*).

## Conclusion

Taken together, the present study was among the first to use RNA-Seq to quantitatively determine the mRNA abundance and transcript variations throughout mouse liver development. Results from our study have unveiled that 1) critical signaling networks and up-stream regulators are co-expressed in an age-specific manner to orchestrate the age-specific biological functions in liver; and 2) age contributes critically to the complexity of the alternative splicing landscape of the hepatic transcriptome. Future studies will aim to cross-reference the ontogeny of the mouse transcriptome with that in developing human livers, and validate the known and novel alternative protein isoforms during liver development.

## Materials and Methods

### Ethics Statement

The animal housing facility at the University of Kansas Medical Center is accredited by the Association for Assessment and Accreditation of Laboratory Animal Care. All procedures were approved by the University of Kansas Medical Center's Institutional Animal Care and Use Committee.

### Animals

Eight-week old C57BL/6 breeding pairs of mice were purchased from The Jackson Laboratory (Bar Harbor, ME). They were housed on corn-cob bedding according to the American Animal Association Laboratory Animal Care guidelines, and were bred under standard conditions at the University of Kansas Medical Center. All animals were given ad libitum access water and standard rodent chow (Harlan Teklad 8604, Halan Teklad, Madison, WI). These breeders were bred overnight and separated the next morning. Pups of the breeders were weaned 21-days of age. Livers from offspring were collected at the following 12 ages: day -2 (GD17.5 embryos from the pregnant mothers were removed for tissue collection), day 0 (right after birth and before the start of suckling), day 1, 3, 5, 10, 15, 20, 25, 30, 45, and 60. Due to potential variations caused by the estrous cycle in maturing adult female mice, only male livers were used for this study (n = 3 per age, randomly selected from multiple litters). Livers were frozen immediately in liquid nitrogen, and stored at -80°C.

### Total RNA Extraction and Sequencing Library Construction

RNA extraction and sequencing library construction were performed according to previously described procedures [41].

### RNA-Seq data

Sequencing was performed in an Illumina HiSeq 2000 sequencing machine (*Illumina*, *San Diego*, *CA*) at a 2 × 100 bp paired-end resolution. Sequence reads were mapped to the mouse reference genome (NCBI37/mm9) using TopHat [65] with default parameters. Transcript abundance estimates were generated using Cufflinks [66] with default parameters. The genome was annotated using RefSeq annotations.

### Gene Expression Rank

Each age sampled in the current study was assigned a rank for each gene considered, called the Gene Expression Rank. This is a number between 1 and 12, describing the relative placement of a gene’s expression level over the 12 ages considered in the study. The age in which the gene is expressed the most was assigned the highest rank of 12. The age in which the gene is expressed the least was assigned the lowest rank of 1. Ties are resolved by assigning the average rank of the ties to the tied ages.

### Factor analysis based clustering

The data matrix of significant differentially expressed genes (7,289) was factor analyzed with three common factors to identify the latent features responsible for the observed expression patterns. The number of factors was determined by a scree plot examination (a graphical display of the eigenvalues associated with a factor in descending order versus the number of factor) and a Horn’s parallel analysis [67] (see S1 Methods). The factor solution was rotated using the 'varimax' procedure for better interpretation. The factor loading matrix was used to generate gene clusters by correlating the gene expression with the factor loading graph. The three factors describing the factor loading graph was identified as described in the text. Genes were associated with the factor with which had the highest absolute correlation, if this correlation was equal to or greater than 0.7, resulting in the three gene clusters. The directionality of correlation determined the sub-classification of expression (positive) or suppression (negative) within the cluster.

### Identifying upstream regulators

Upstream regulators associated with genes in each group were identified using Ingenuity Systems IPA software (*www*.*ingenuity*.*com*). IPA consists of a comprehensive knowledge base of known molecular interactions, including information on the directionality of expression resulting from the interactions. Using this information, IPA computes an activation z-score for upstream regulators indicating whether a regulator is activated of inhibited, based on the directionality of expression of the genes in the gene set that is associated with the regulator. If the up- and down-regulation patterns of these genes are in concordance with information on the expected directionality of expression of these genes in the presence of the regulator, a positive activation z-score is recorded. If it is opposite to what is expected, a negative activation z-score is recorded. For each group, all upstream regulators that had an absolute activation z-score greater than or equal to 2 in at least one of the days were selected for analysis.

### Functional analysis

Biological functional and pathway analysis of genes was performed using Ingenuity Systems IPA software (*www*.*ingenuity*.*com*). The enriched functions and pathways for a set of genes was inferred from the *p-value* of the measure of likelihood of the overlap between genes in the gene set, and genes in the biological function or pathway calculated using the right tailed Fisher’s exact test. Pathways and functions with a *p-value* cutoff less than or equal to 0.05 were considered significant. IPA also calculated an activation z-score for gene sets’ association with biological functions similar to the one described for upstream regulators. A positive activation z-score signifies an increase in the biological function and a negative score signifies a decrease in the function. As with upstream regulators, this calculation encompasses information on both the number and directionality of expression of genes in the dataset. Biological functions with an absolute activation z-score greater than or equal to 2 were considered significant.

### Identifying upstream regulatory modules

The underlying procedure was developed to identify clusters of upstream regulators with similar or diametrical expression patterns that target a sub-set of genes. An iterative clustering algorithm was developed for this purpose. Upstream regulators and their target genes were obtained as described above. The similarity of the pattern of expression of a set of upstream regulators was measured by the intraclass correlation (ICC) using Winer's adjustment for anchor points approach. The algorithm starts by merging all pairs of upstream regulators to form a list of 2-tuples (upstream regulators, target genes), subjected to the constraints that the absolute value of the ICC of the upstream regulators in the merged set is greater than or equal to 0.6 (ICC_cutoff) and the new target set has at least 5 genes. Every successful merge operation adds a new entry to the list. A new cycle combines all pairs of entries and subject to the above constrains with the newly added entries of the previous cycle. This process is repeated until no successful merge operation is performed in a cycle.

### Identifying novel isoforms

Novel isoformes were scanned for using Cufflinks [62] after re-mapping reads to the latest mouse reference genome (GRCm38/mm10) using TopHat [65]. The expression of each novel isoform at each age was measured for significance using the same statistical procedure adopted for measuring the significance of gene expression described in **S1 Methods**. Novel isoforms significantly expressed in at least one age were further filtered on the significance of their Durbin-Watson statistic [68] for autocorrelation, after adjusting for multiple-hypothesis testing using the Benjamini-Hochberg procedure [69]. The second filtering step is intended to capture the time-correlated nature of transcript expression. The type of splicing event characterizing novel isoforms was identified using the program ASprofile [70]. Images along with comparative annotations from RefSeq, Ensembl, and UCSC of example novel isoforms were generated from the UCSC Genome Browser [71].

## Supporting Information

S1 Fig Distribution of the Pearson’s correlation coefficient between genes in the present study and genes in the Li et al [32] study. (PDF)

S2 Fig Distribution of the total FPKM value at each age. (PDF)

S3 Fig The distribution of cellular localization of the *analysis-genes*. (PDF)

S4 Fig Expression patterns of the different splice variants. Expression patterns of the different splice variants of the 90 genes (see text) with splice forms that are significantly different in their expression pattern along the developmental gradient.
(PDF)

S5 Fig The top 15 genes with the highest number of targeting upstream-regulators. The top 15 genes with the highest number of targeting upstream-regulators in the Prenatal and Neonatal, Prenatal and Adult, Neonatal, Neonatal and Adolescent, Adolescent and Adult, and, Adult, groups respectively.
(PDF)

S1 Methods Supplemental Methods. (PDF)

S1 Table Correlation between RNA-Seq and RT-PCR. (XLSX)

S2 Table Average gene expression. (XLSX)

S3 Table Distribution of genes according to molecular type. (XLSX)

S4 Table Distribution of molecular type of the significantly. (XLSX)

S5 Table Mean cumulative expression of molecular types. (XLSX)

S6 Table Transcript level gene expression. (XLSX)

S7 Table Distribution of the number of significantly expressed known transcript variants per gene. (XLSX)

S8 Table Distribution of genes in development period according to molecular type. (XLSX)

S9 Table Distribution of genes in development period according to cellular location. (XLSX)

S10 Table Development dynamics of significant biological functions associated with the cluster of Prenatal and Neonatal genes. (XLSX)

S11 Table Development dynamics of significant biological functions associated with the cluster of Neonatal genes. (XLSX)

S12 Table Development dynamics of significant biological functions associated with the cluster of Neonatal and Adolescent genes. (XLSX)

S13 Table Development dynamics of significant biological functions associated with the cluster of Adolescent and Adult genes. (XLSX)

S14 Table Development dynamics of significant biological functions associated with the cluster of Adult genes. (XLSX)

S15 Table Matrix of hits indicating number of genes in development period targeted by upstream regulator. (XLSX)

S16 Table Hypergeometric p-value of the significance of association of upstream regulators in each sub-cluster (Fig 6B) with genes associated with each temporal group. (XLSX)

S17 Table Upstream regulator distribution on molecular type. (XLSX)

S18 Table Upstream regulatory module (Prenatal and Neonatal). (XLSX)

S19 Table Upstream regulatory module (Prenatal and Adult). (XLSX)

S20 Table Upstream regulatory module (Neonatal). (XLSX)

S21 Table Upstream regulatory module (Neonatal and Adolescence). (XLSX)

S22 Table Upstream regulatory module (Adolescence and Adult). (XLSX)

S23 Table Upstream regulatory module (Adult). (XLSX)

S24 Table Genes with novel isoforms. (XLSX)

S25 Table Alternative splicing event classification. (XLSX)
