# Supplementary figures and images for: Deciphering the Developmental Dynamics of the Mouse Liver Transcriptome
Source: PLoS One. 2015 Oct 23;10(10):e0141220. doi: 10.1371/journal.pone.0141220 (PMC4619800; doi:10.1371/journal.pone.0141220)

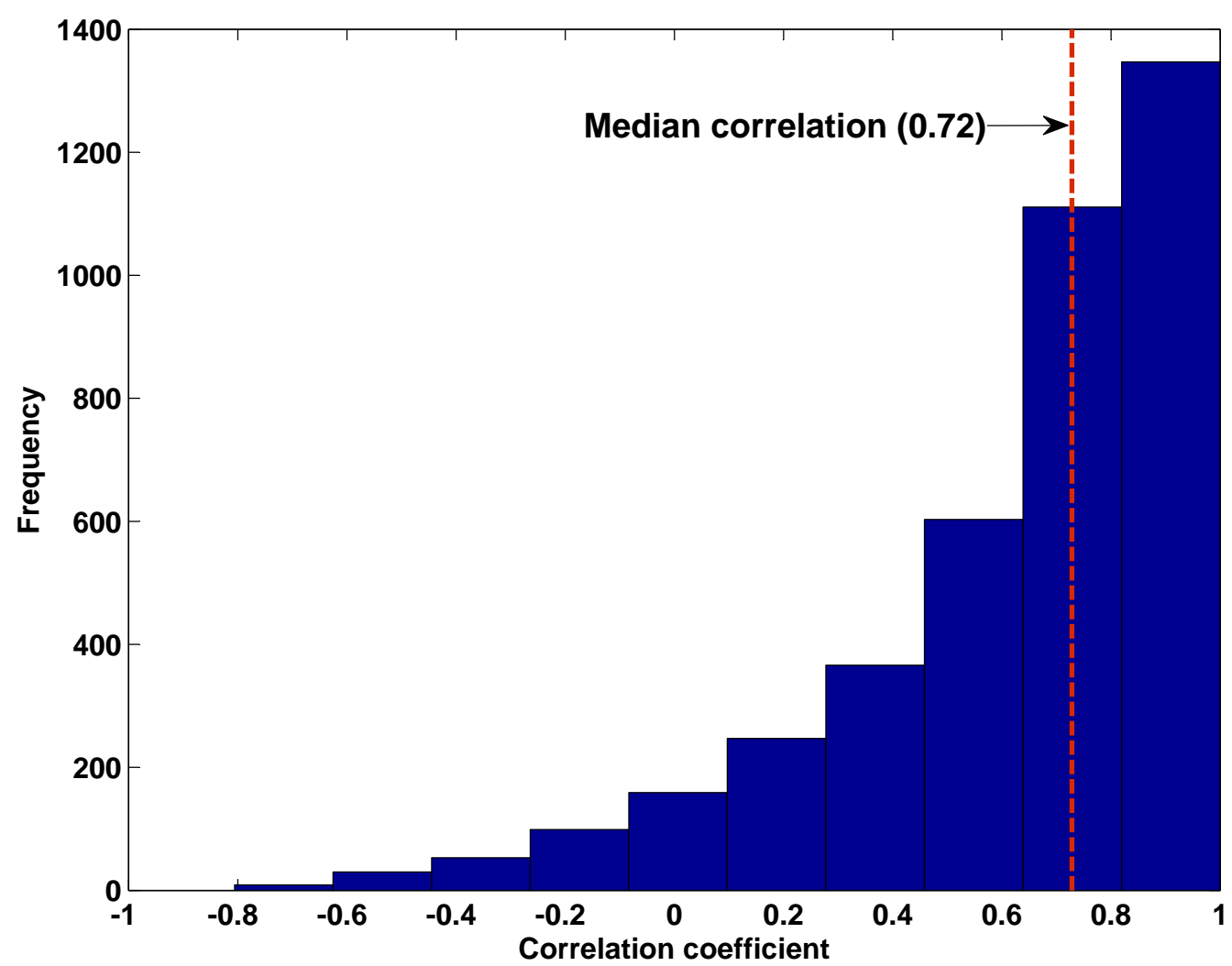

Supplement: S1 Fig — (PDF) [file pone.0141220.s001.pdf]

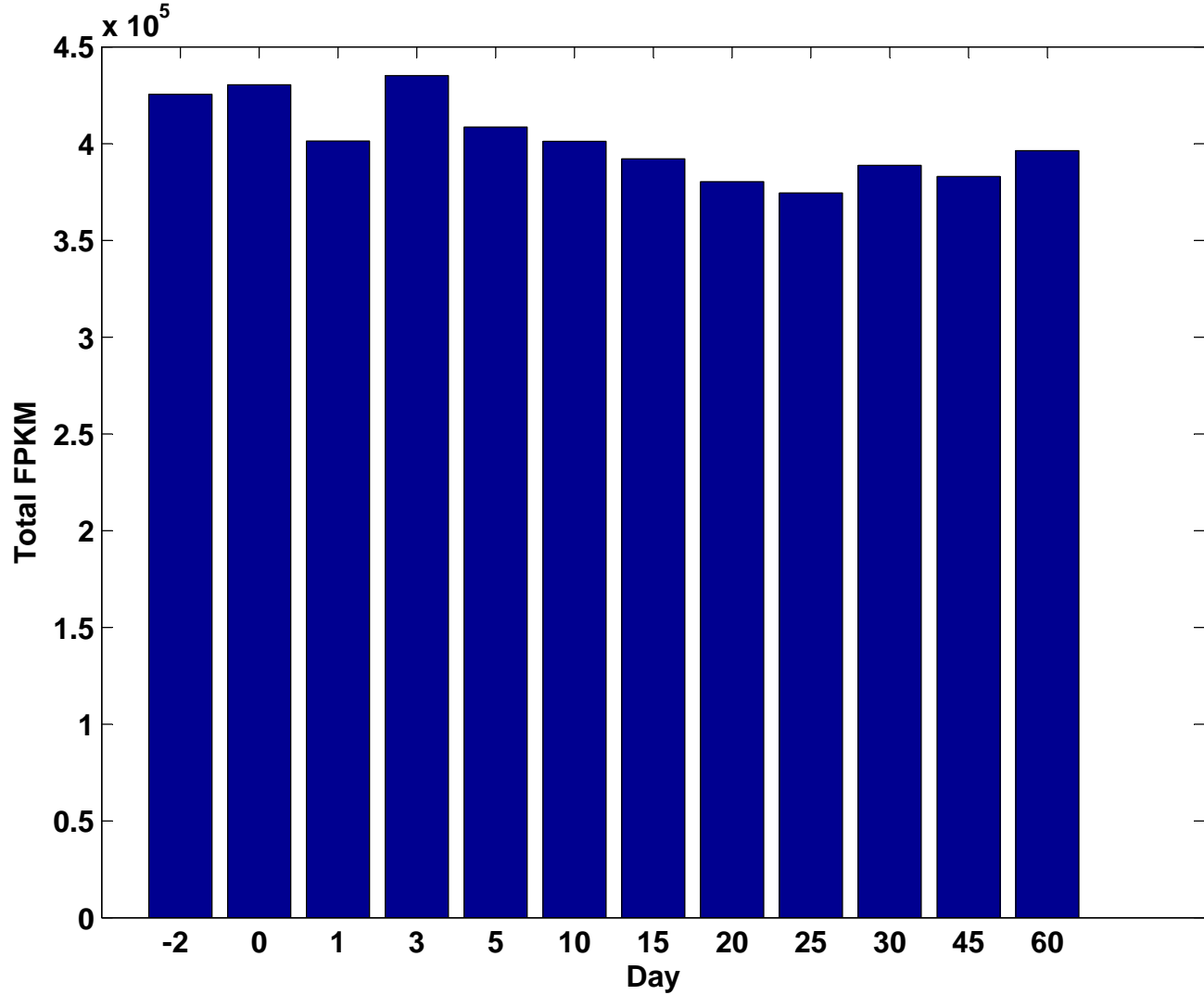

Supplement: S2 Fig — (PDF) [file pone.0141220.s002.pdf]

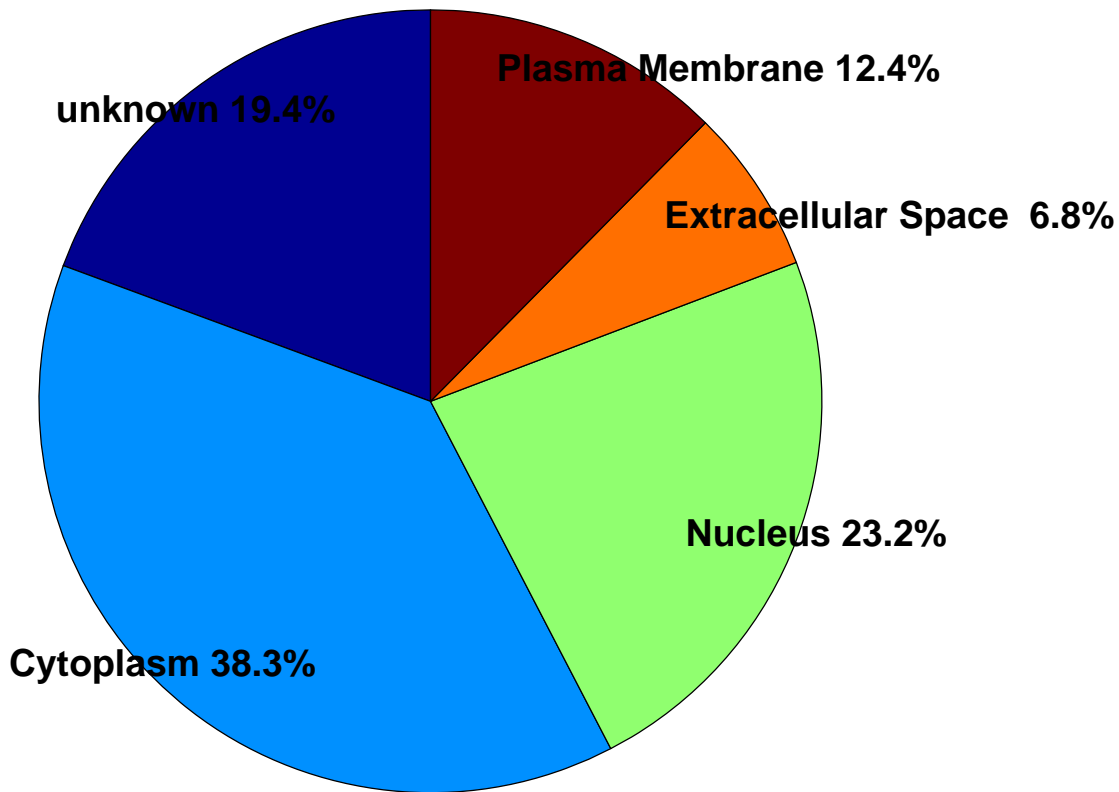

Supplement: S3 Fig — (PDF) [file pone.0141220.s003.pdf]

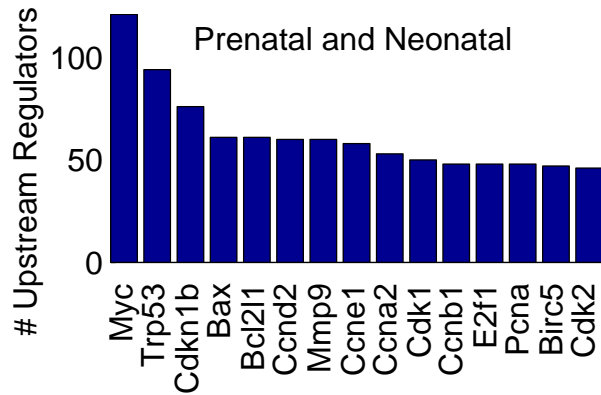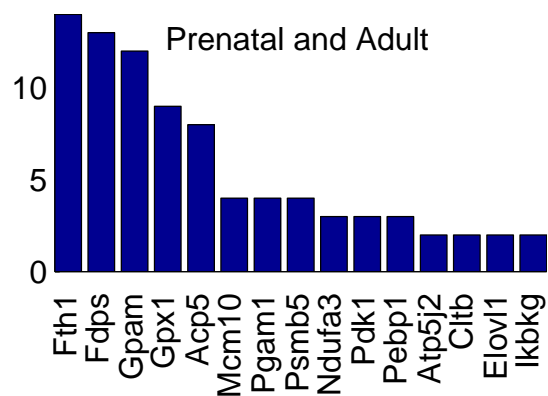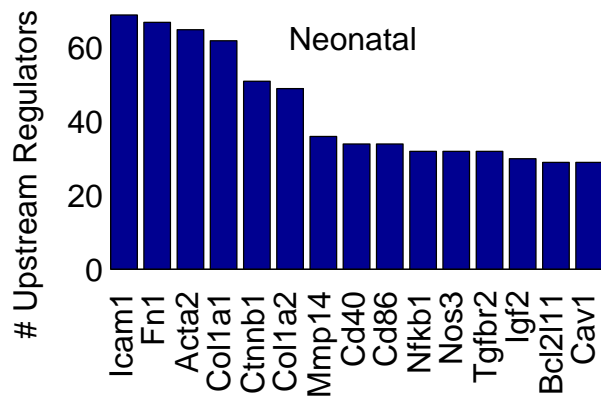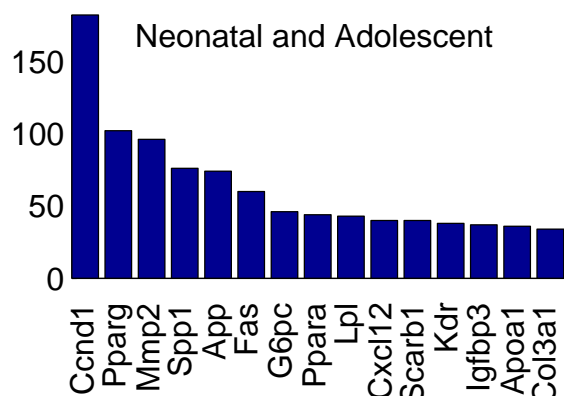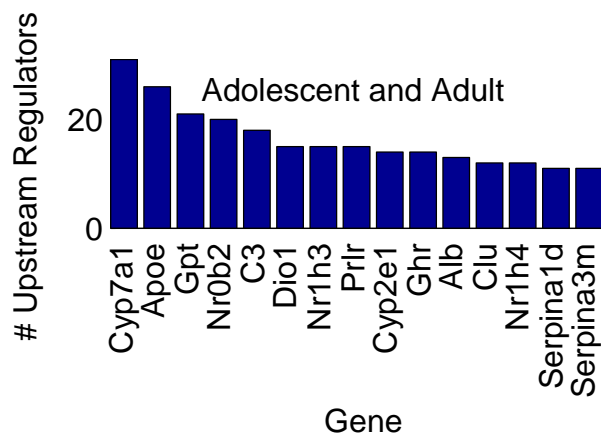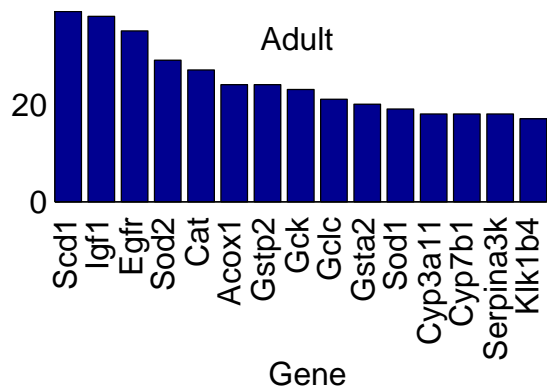

Supplement: S5 Fig — The top 15 genes with the highest number of targeting upstream-regulators in the Prenatal and Neonatal, Prenatal and Adult, Neonatal, Neonatal and Adolescent, Adolescent and Adult, and, Adult, groups respectively. (PDF) [file pone.0141220.s005.pdf]
