# Supplementary material for: Deciphering the Developmental Dynamics of the Mouse Liver Transcriptome
Source: PLoS One. 2015 Oct 23;10(10):e0141220. doi: 10.1371/journal.pone.0141220 (PMC4619800; doi:10.1371/journal.pone.0141220)
Supplement: S4 Fig — Expression patterns of the different splice variants of the 90 genes (see text) with splice forms that are significantly different in their expression pattern along the developmental gradient. (PDF) [file pone.0141220.s004.pdf]

Page	Gene	Transcripts
3	**1700019G17Rik**	NM_001145895,NM_029331
4	**2810007J24Rik**	NM_001199306,NM_175250
5	**5133401N09Rik**	NM_001048060,NM_198004
6	**Acot7**	NM_001146057,NM_133348
7	**Acp5**	NM_001102404,NM_001102405,NM_007388
8	**Add1**	NM_001024458,NM_001102444,NM_013457
9	**Anapc11**	NM_001038230,NM_025389
10	**Anapc5**	NM_001042491,NM_021505
11	**Aplp2**	NM_001102455,NM_009691
12	**Apoc1**	NM_001110009,NM_007469
13	**Arap1**	NM_001040111,NM_001040112
14	**Atp5g1**	NM_001161419,NM_007506
15	**Bscl2**	NM_001136064,NM_008144
16	**Cbs**	NM_144855,NM_178224
17	**Ccnd3**	NM_001081635,NM_001081636,NM_007632
18	**Cdipt**	NM_026638,NM_138754
19	**Comt**	NM_001111062,NM_001111063,NM_007744
20	**Cuta**	NM_026307,NM_026948
21	**Cyth1**	NM_001112699,NM_001112700
22	**Dapk3**	NM_001190473,NM_001190474
23	**Dcaf11**	NM_001199009,NM_133734,NR_037572
24	**Egfl7**	NM_001164564,NM_178444,NM_198724,NM_198725
25	**Egfr**	NM_007912,NM_207655
26	**Elovl1**	NM_001039176,NM_019422
27	**Eng**	NM_001146348,NM_007932
28	**Fis1**	NM_001163243,NM_025562
29	**Fiz1**	NM_001110328,NM_001110329
30	**Fkbp8**	NM_001111066,NM_001199631,NM_010223
31	**Gcdh**	NM_001044744,NM_008097
32	**Gorasp2**	NM_027352,NR_027343
33	**Gpr146**	NM_001038703,NM_030258
34	**Gpr56**	NM_001198894,NM_018882
35	**H2afy**	NM_001159513,NM_001159514,NM_001159515,NM_012015
36	**Hmbs**	NM_001110251,NM_013551
37	**Hras1**	NM_001130443,NM_008284
38	**Ifi27l1**	NM_026790,NM_194067,NM_194068,NM_194069
39	**Ifnar2**	NM_001110498,NM_010509
40	**Il11ra1**	NM_001163401,NM_010549
41	**Insig2**	NM_133748,NM_178082
42	**Ivns1abp**	NM_001039511,NM_054102
43	**Kng1**	NM_001102411,NM_001102412,NM_023125
44	**Ldb1**	NM_001113408,NM_010697
45	**Ldha**	NM_001136069,NM_010699
46	**Lrch4**	NM_001168652,NM_146164
47	**Lrrc8d**	NM_001122768,NM_178701
48	**Ly6e**	NM_001164036,NM_001164038,NM_001164039,NM_001164040,NM_008529
49	**Mars**	NM_001003913,NM_001171582
50	**Masp2**	NM_001003893,NM_010767
51	**Mcfd2**	NM_139295,NM_176808
52	**Mid1ip1**	NM_001166635,NM_026524

53	**Mpst**	NM_001162492,NM_001162493,NM_138670
54	**Mtus1**	NM_001005863,NM_001005865
55	**Nadk**	NM_001159637,NM_138671
56	**Ndrg2**	NM_001145959,NM_013864
57	**Ndufv3**	NM_001083891,NM_030087
58	**Nfe2l1**	NM_001130450,NM_008686
59	**Nme2**	NM_001077529,NM_008705
60	**Nr1h3**	NM_001177730,NM_013839
61	**Pacsin2**	NM_001159509,NM_011862
62	**Pcx**	NM_001162946,NM_008797
63	**Pfkm**	NM_001163487,NM_001163488
64	**Pklr**	NM_001099779,NM_013631
65	**Pnkd**	NM_019999,NM_025580
66	**Ppp2r5c**	NM_001081457,NM_001081458,NM_001135001
67	**Pqlc1**	NM_001164420,NM_001164421,NM_025861
68	**Prkd3**	NM_001171004,NM_001171005
69	**Psen2**	NM_001128605,NM_011183
70	**Ptk2b**	NM_001162365,NM_172498
71	**Ptpn6**	NM_001077705,NM_013545
72	**Puf60**	NM_001164600,NM_028364,NM_133691
73	**Rab43**	NM_001039394,NM_133717
74	**Rnf14**	NM_001164622,NM_020012
75	**Rnh1**	NM_001172100,NM_001172101,NM_145135
76	**Rtn4**	NM_024226,NM_194052
77	**Sh3bp2**	NM_001145858,NM_011893
78	**Shisa5**	NM_025858,NM_026381
79	**Slc11a2**	NM_001146161,NM_008732
80	**Slc29a1**	NM_001199113,NM_001199115,NM_001199116,NM_022880
81	**Slc38a3**	NM_001199217,NM_001199218,NM_023805
82	**Slc3a2**	NM_001161413,NM_008577
83	**Sphk2**	NM_020011,NM_203280
84	**Stat5b**	NM_001113563,NM_011489
85	**Suds3**	NM_001122666,NM_178622
86	**Tcf25**	NM_001037877,NM_001037878,NM_025804
87	**Timd2**	NM_001161355,NM_134249
88	**Tmbim6**	NM_001171034,NM_001171035,NM_001171036,NM_026669
89	**Tmem176b**	NM_001164207,NM_001164208,NM_001164209,NM_023056
90	**Tsen34**	NM_001164204,NM_024168
91	**Wars**	NM_001164314,NM_011710
92	**Xrcc6bp1**	NM_001159559,NM_026858

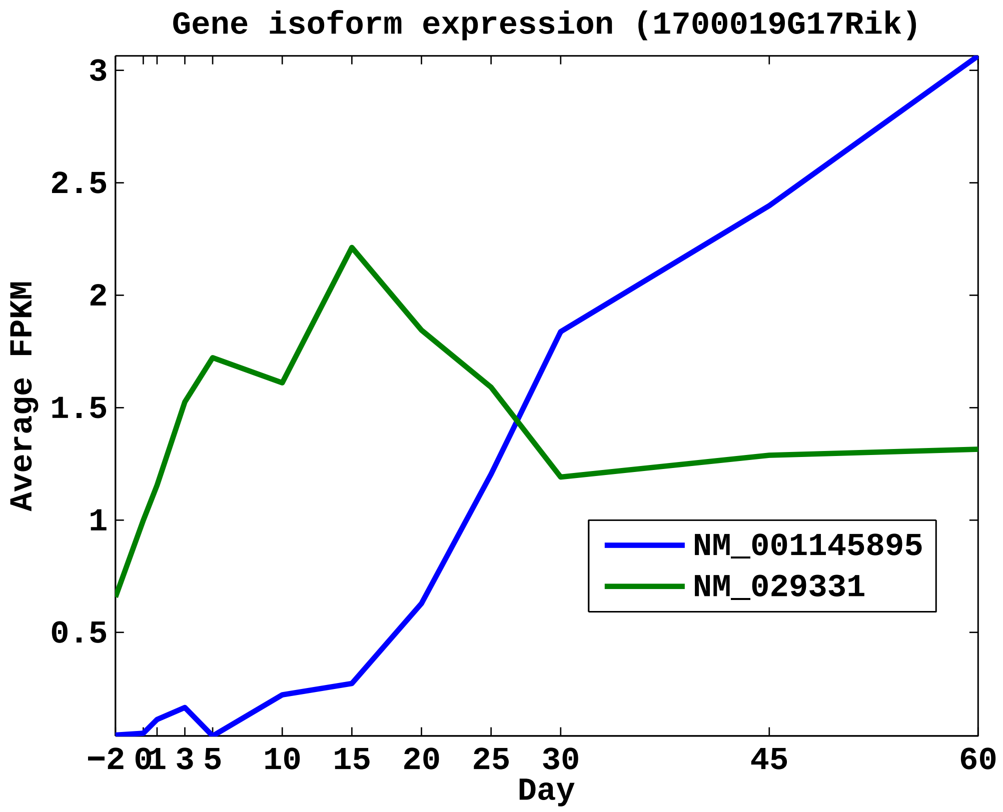

Gene isoform expression (1700019G17Rik)
Average FPKM
3
2.5
2
1.5
1
0.5
−2 0 1 3 5 10 15 20 25 30 45 60
Day
NM_001145895
NM_029331

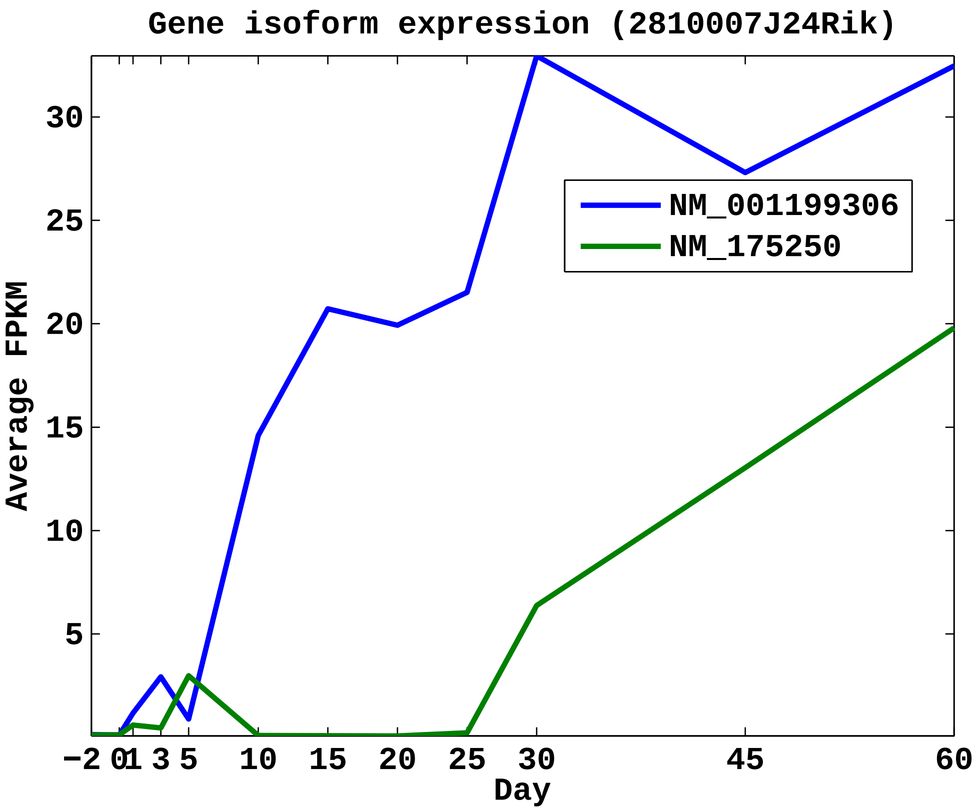

Gene isoform expression (2810007J24Rik)
30
25
20
15
10
5
Average FPKM
−2
0
1
3
5
10
15
20
25
30
45
60
Day
NM_001199306
NM_175250

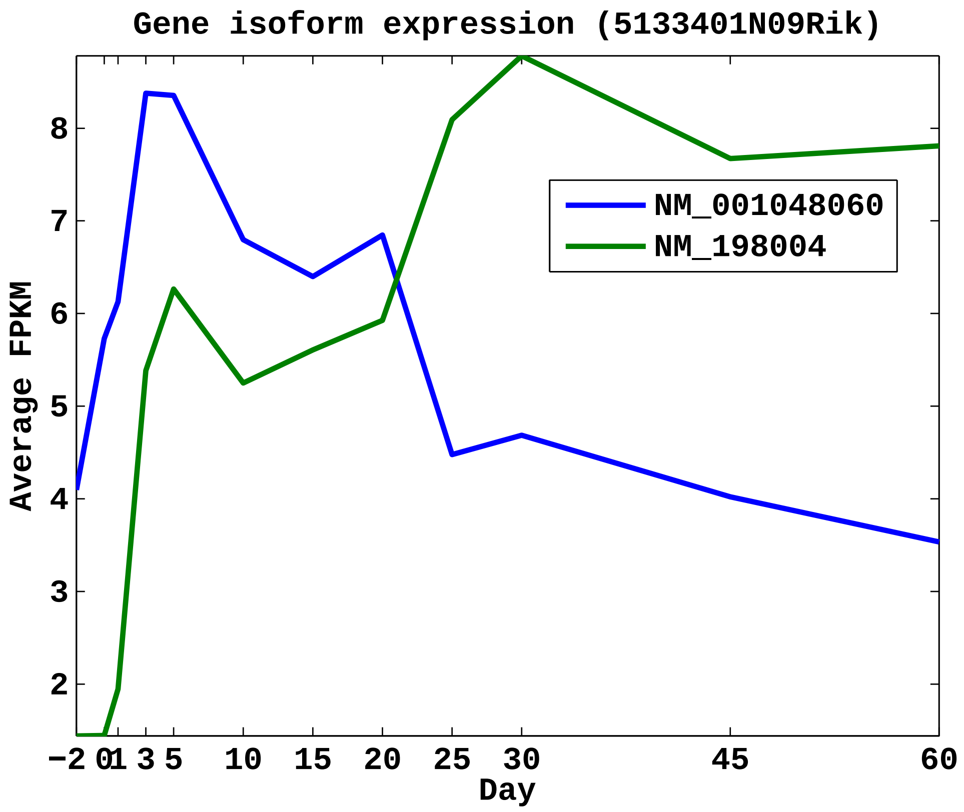

Gene isoform expression (5133401N09Rik)
NM_001048060
NM_198004
Average FPKM
Day
2
3
4
5
6
7
8
−2
0
1
3
5
10
15
20
25
30
45
60

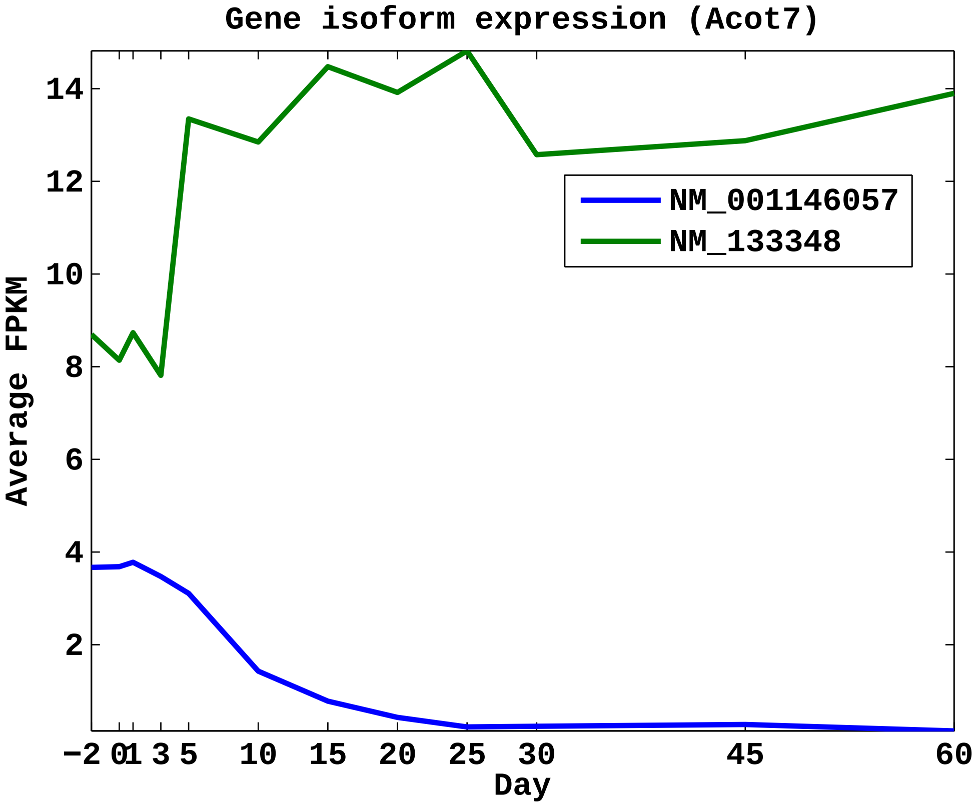
Gene isoform expression (Acot7)
NM_001146057
NM_133348
Average FPKM
14
12
10
8
6
4
2
−2 0 1 3 5 10 15 20 25 30 45 60
Day

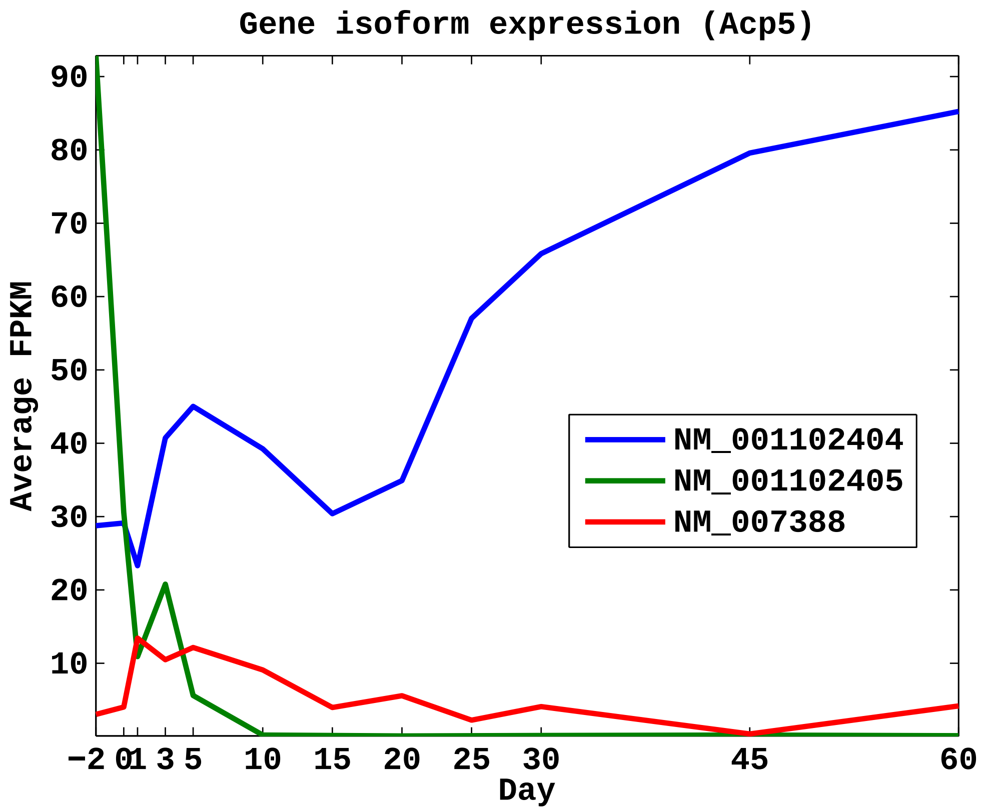
Gene isoform expression (Acp5)
Average FPKM
90
80
70
60
50
40
30
20
10
−2
0
1
3
5
10
15
20
25
30
45
60
Day
NM_001102404
NM_001102405
NM_007388

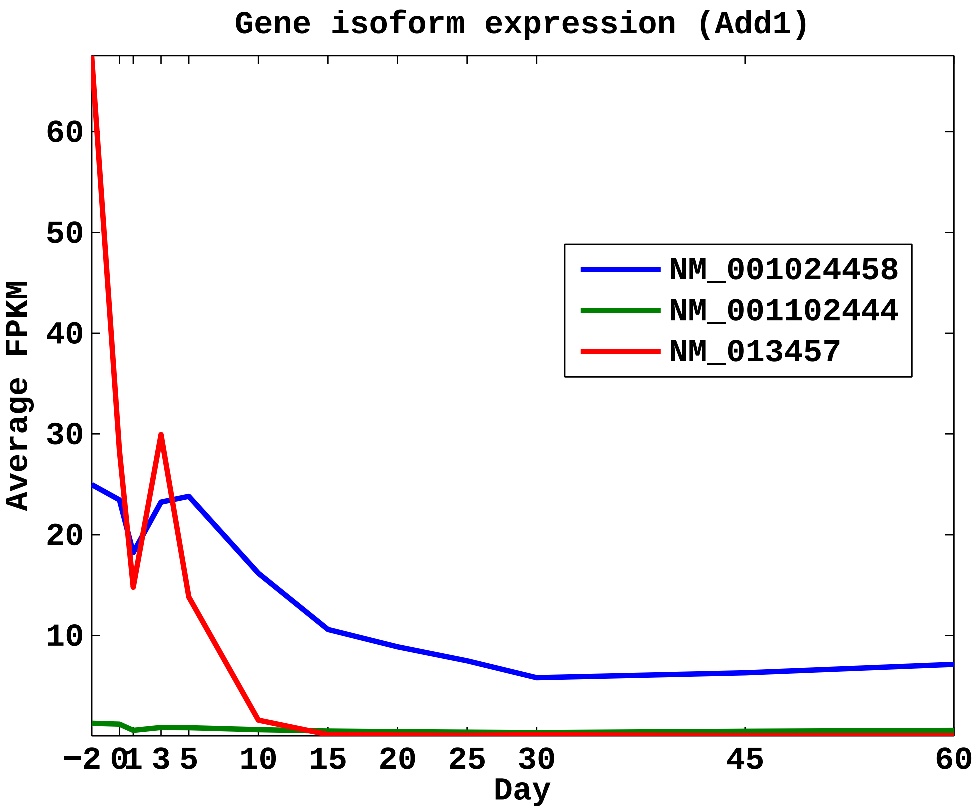

Gene isoform expression (Add1)
Average FPKM
60
50
40
30
20
10
NM_001024458
NM_001102444
NM_013457
−2
0
1
3
5
10
15
20
25
30
45
60
Day

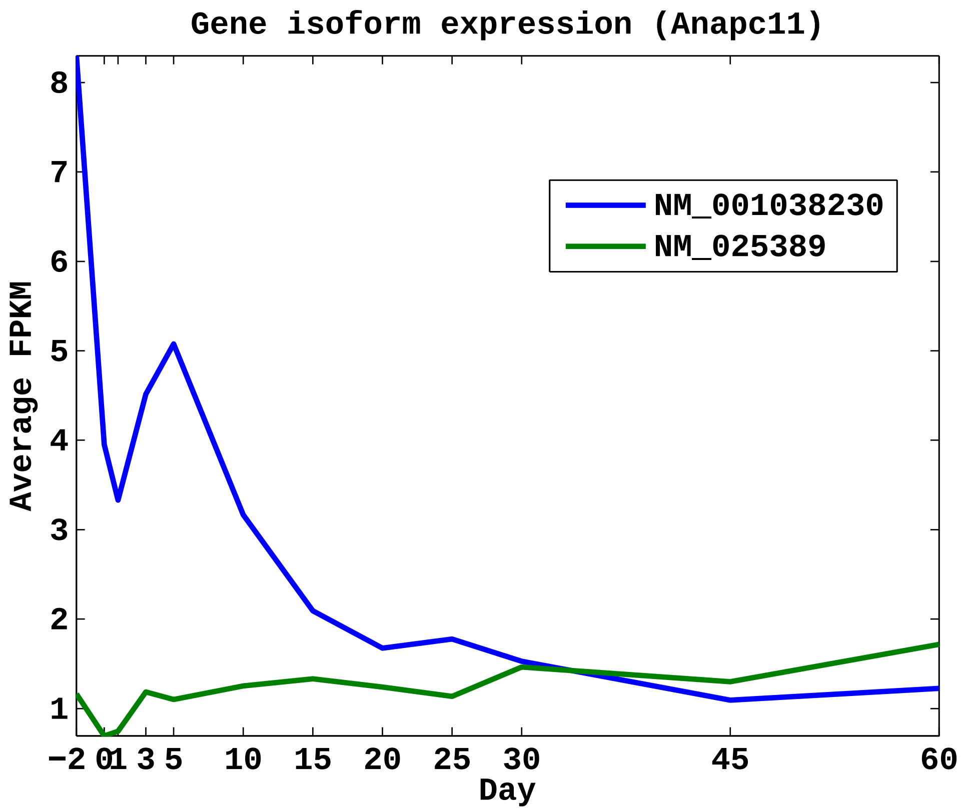

Gene isoform expression (Anapc11)
Average FPKM
1
2
3
4
5
6
7
8
NM_001038230
NM_025389
−2
0
1
3
5
10
15
20
25
30
45
60
Day

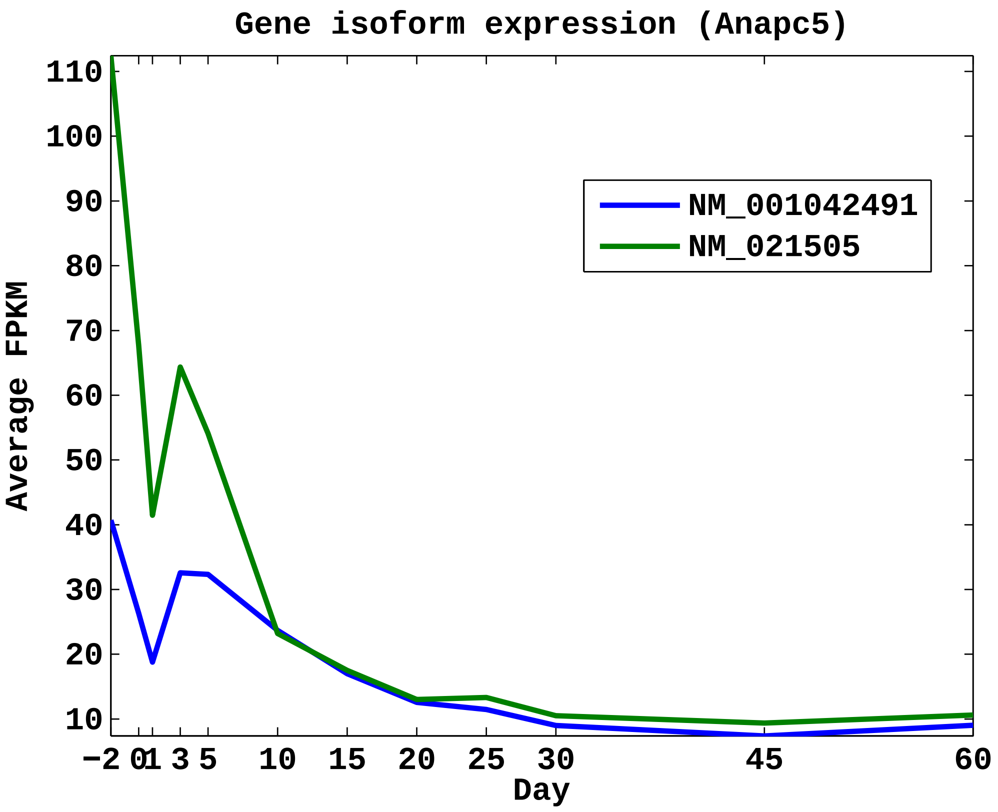

Gene isoform expression (Anapc5)
Average FPKM
Day
NM_001042491
NM_021505
110
100
90
80
70
60
50
40
30
20
10
−2 0 1 3 5 10 15 20 25 30 45 60

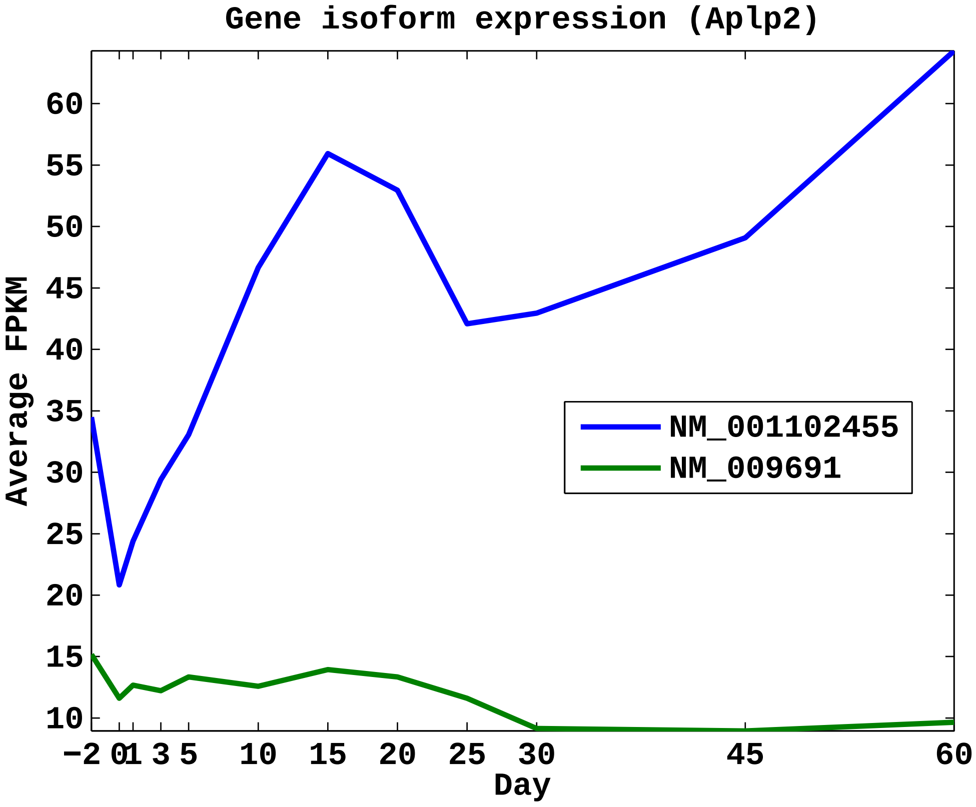

Gene isoform expression (Aplp2)
Average FPKM
60
55
50
45
40
35
30
25
20
15
10
−2
0
1
3
5
10
15
20
25
30
45
60
Day
NM_001102455
NM_009691

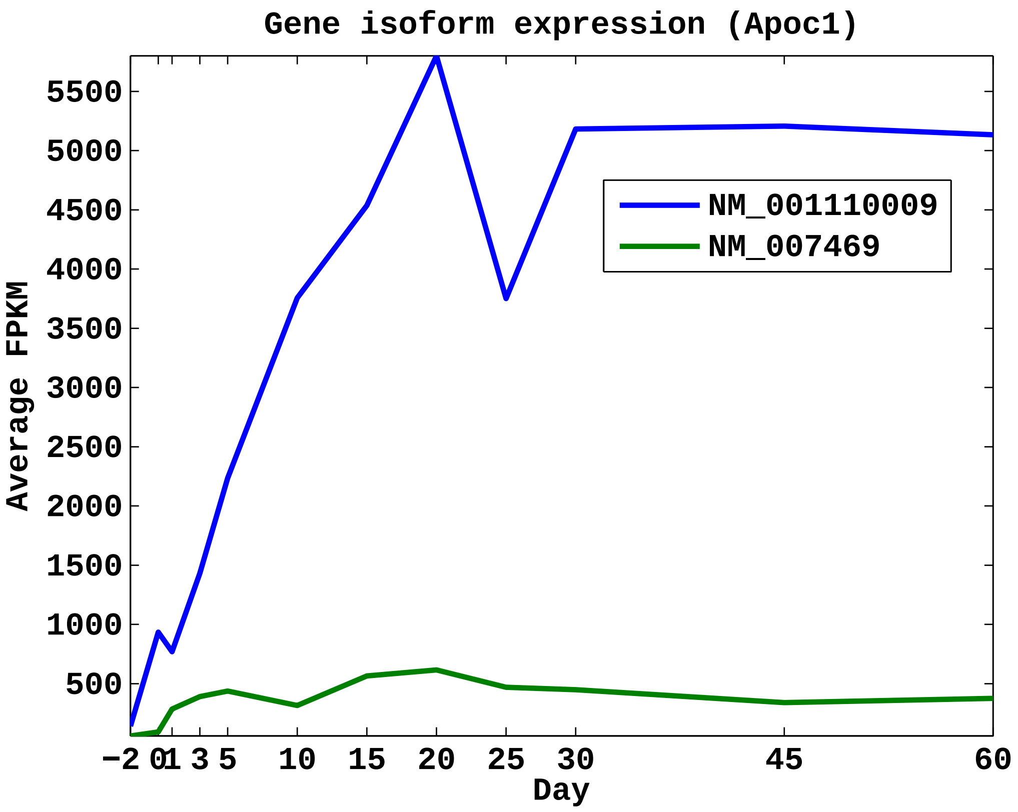

Gene isoform expression (Apoc1)
Average FPKM
5500
5000
4500
4000
3500
3000
2500
2000
1500
1000
500
NM_001110009
NM_007469
−2
0
1
3
5
10
15
20
25
30
45
60
Day

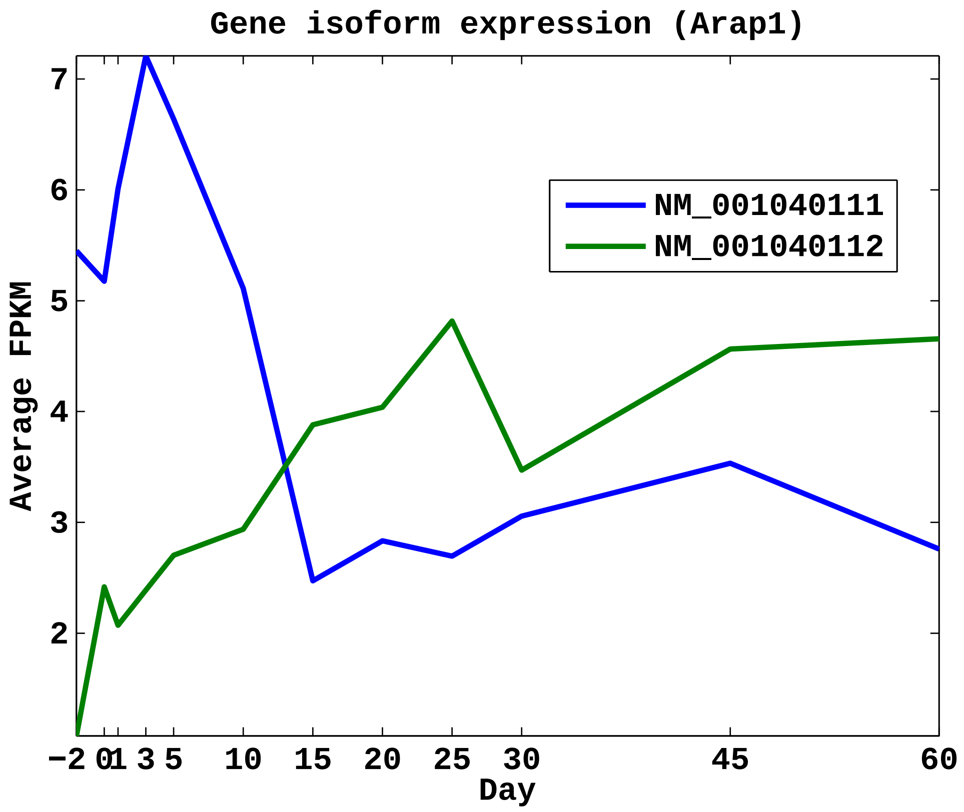

Gene isoform expression (Arap1)
Average FPKM
Day
NM_001040111
NM_001040112
7
6
5
4
3
2
−2
0
1
3
5
10
15
20
25
30
45
60

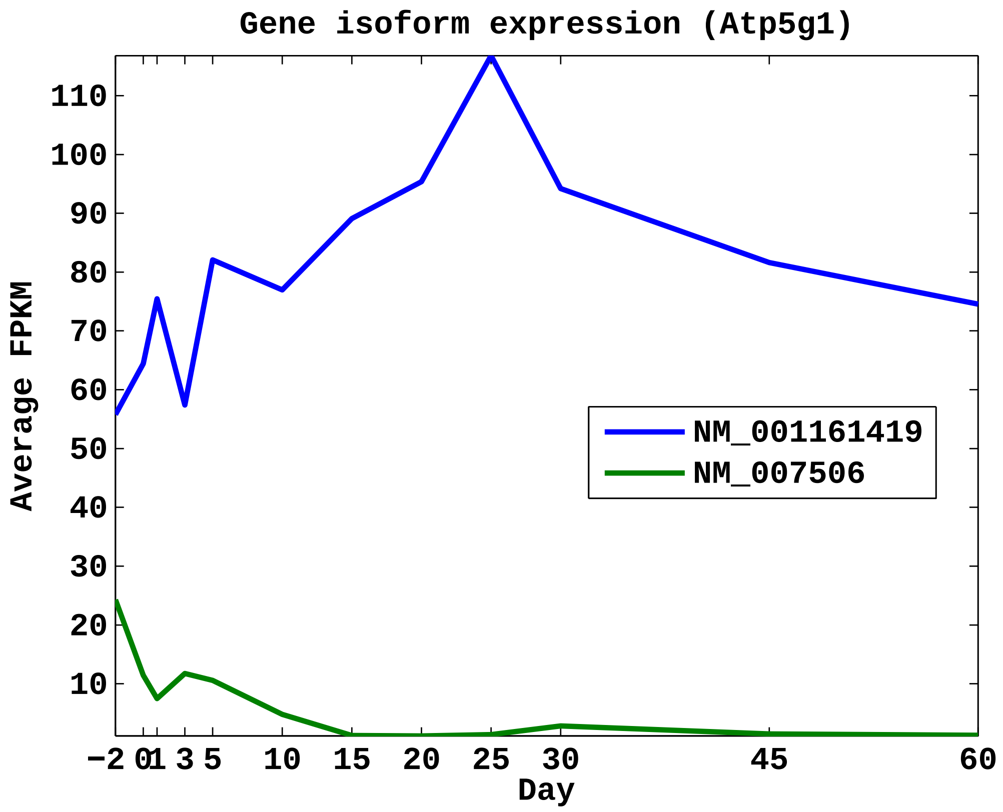
Gene isoform expression (Atp5g1)
Average FPKM
Day
110
100
90
80
70
60
50
40
30
20
10
−2
0
1
3
5
10
15
20
25
30
45
60
NM_001161419
NM_007506

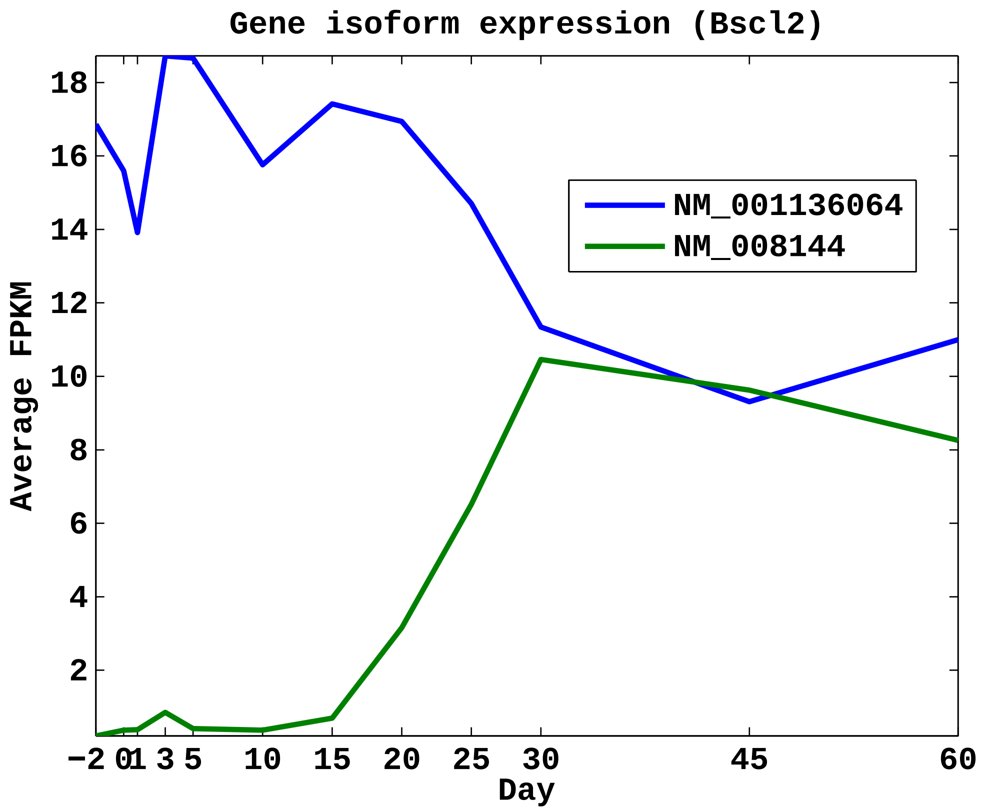

Gene isoform expression (Bscl2)
Average FPKM
18
16
14
12
10
8
6
4
2
−2 0 1 3 5 10 15 20 25 30 45 60
Day
NM_001136064
NM_008144

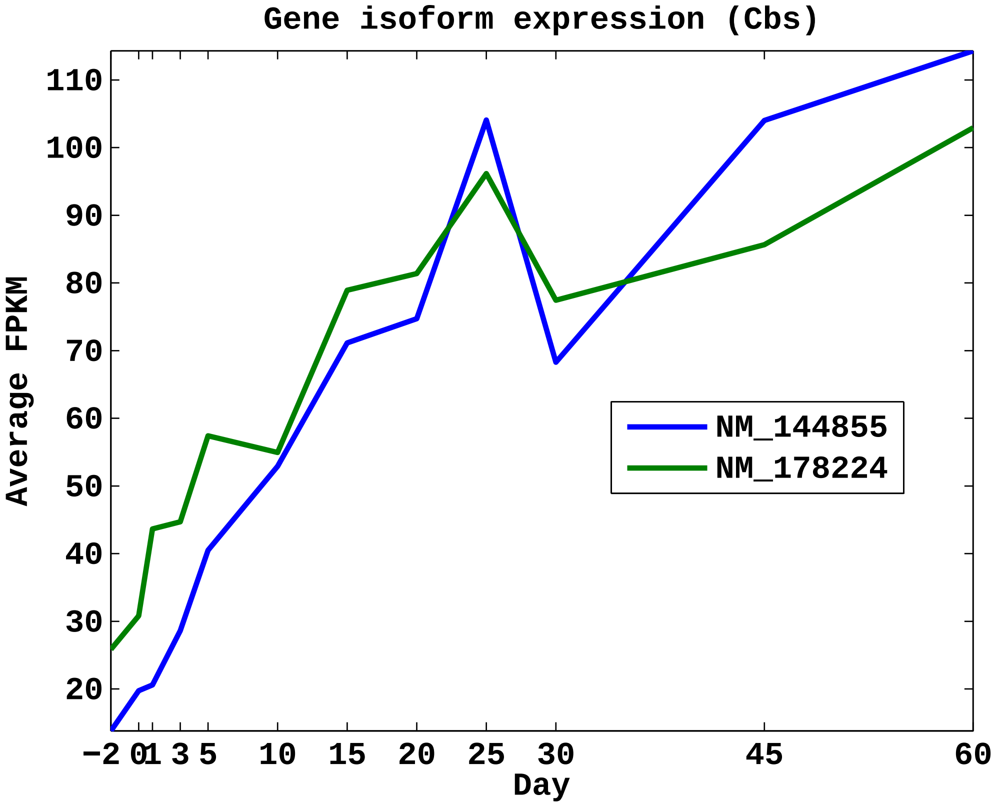
Gene isoform expression (Cbs)
Average FPKM
110
100
90
80
70
60
50
40
30
20
−2 0 1 3 5 10 15 20 25 30 45 60
Day
NM_144855
NM_178224

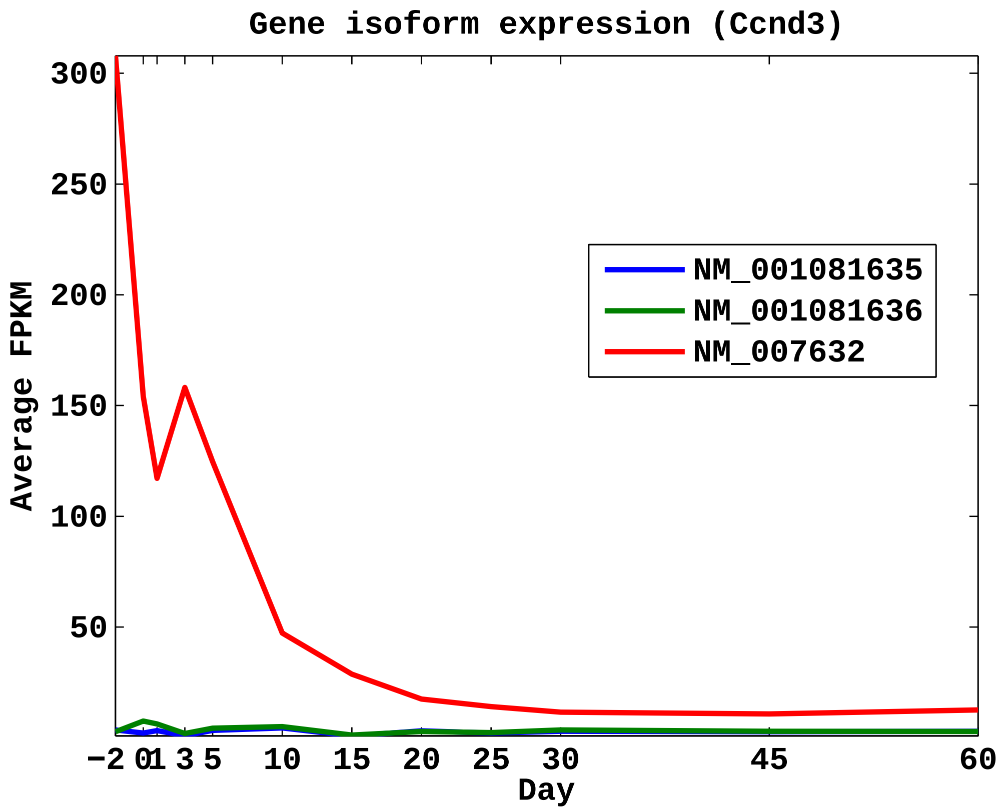

Gene isoform expression (Ccnd3)
Average FPKM
300
250
200
150
100
50
−2
0
1
3
5
10
15
20
25
30
45
60
Day
NM_001081635
NM_001081636
NM_007632

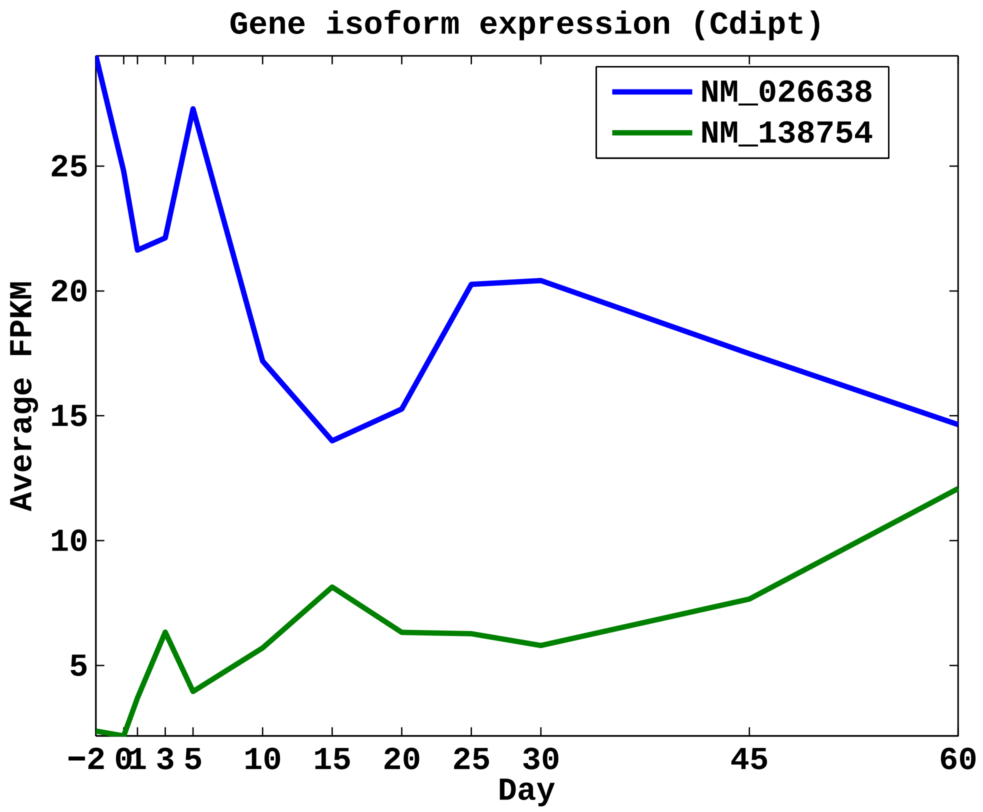

Gene isoform expression (Cdipt)
NM_026638
NM_138754
Average FPKM
25
20
15
10
5
−2
0
1
3
5
10
15
20
25
30
45
60
Day

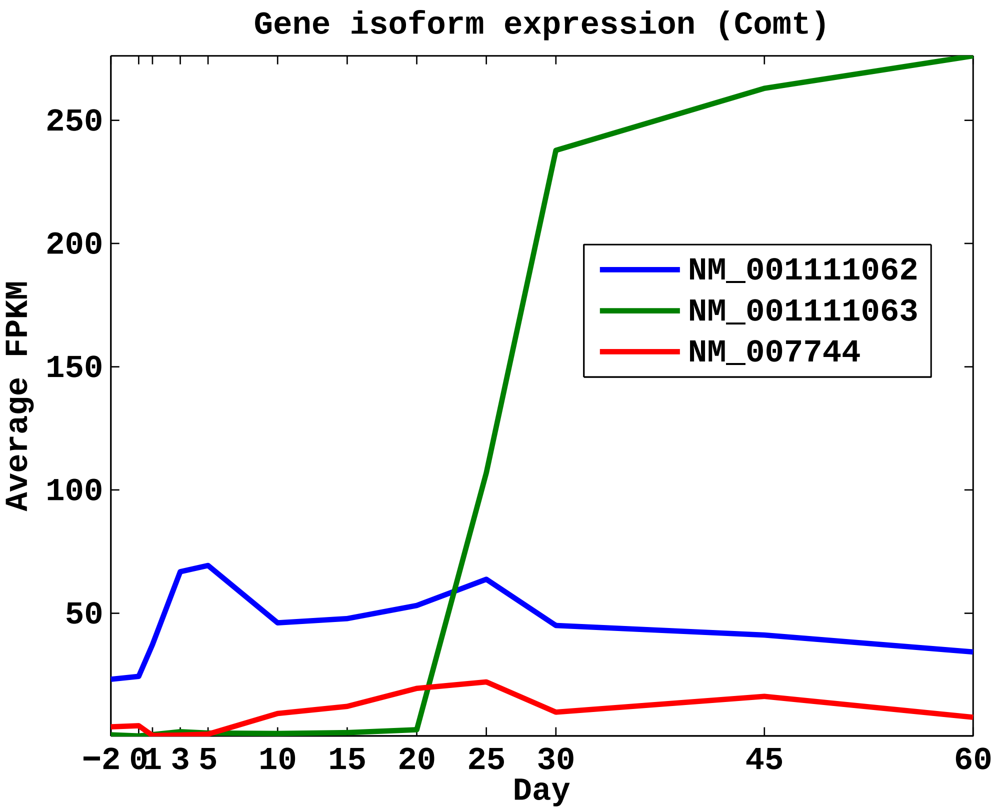
Gene isoform expression (Comt)
Average FPKM
250
200
150
100
50
−2
0
1
3
5
10
15
20
25
30
45
60
Day
NM_001111062
NM_001111063
NM_007744

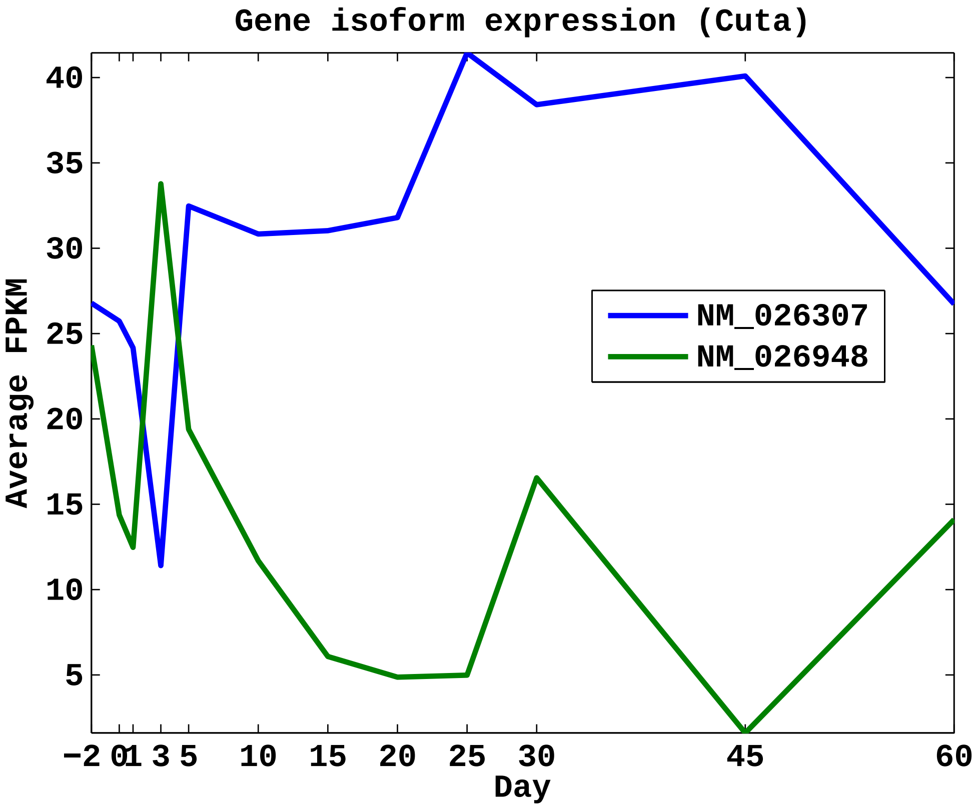

Gene isoform expression (Cuta)
Average FPKM
40
35
30
25
20
15
10
5
−2
0
1
3
5
10
15
20
25
30
45
60
Day
NM_026307
NM_026948

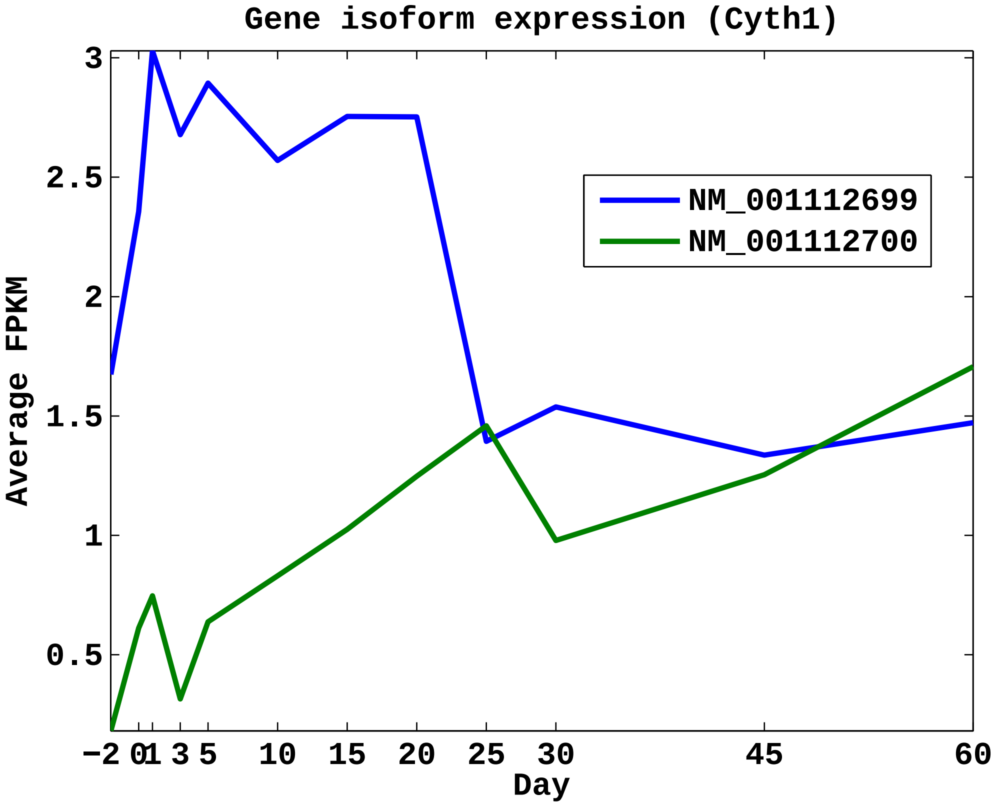

Gene isoform expression (Cyth1)
NM_001112699
NM_001112700
Average FPKM
Day
3
2.5
2
1.5
1
0.5
−2 0 1 3 5 10 15 20 25 30 45 60

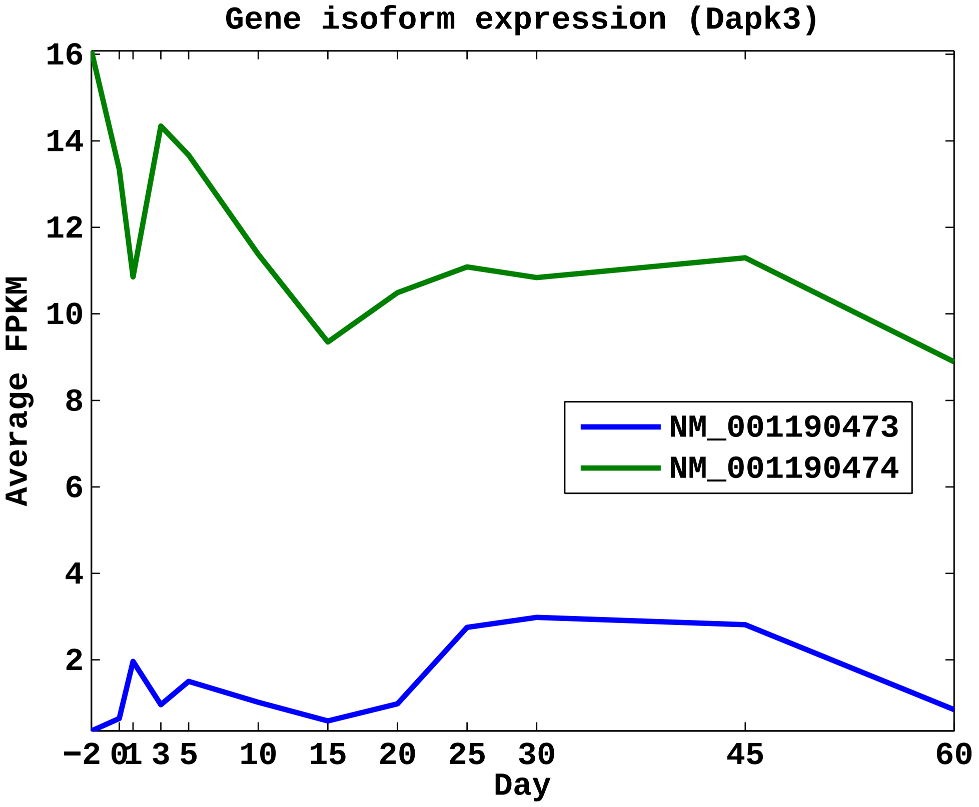

Gene isoform expression (Dapk3)
Average FPKM
16
14
12
10
8
6
4
2
−2
0
1
3
5
10
15
20
25
30
45
60
Day
NM_001190473
NM_001190474

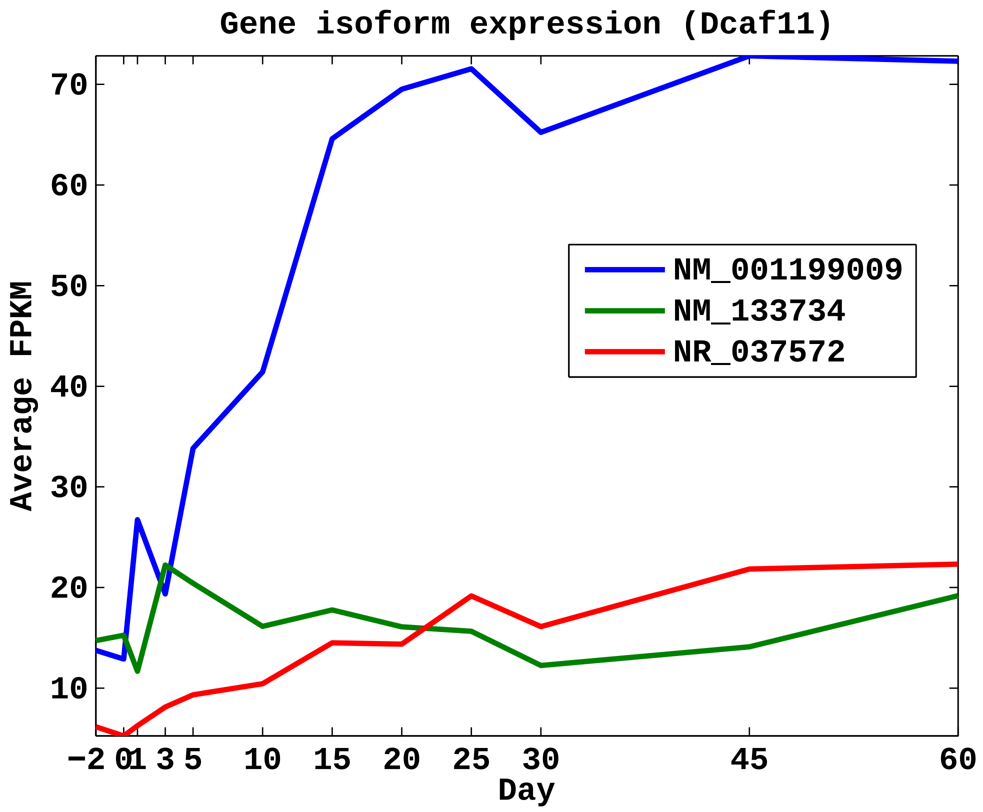

Gene isoform expression (Dcaf11)
Average FPKM
70
60
50
40
30
20
10
−2 0 1 3 5 10 15 20 25 30 45 60
Day
NM_001199009
NM_133734
NR_037572

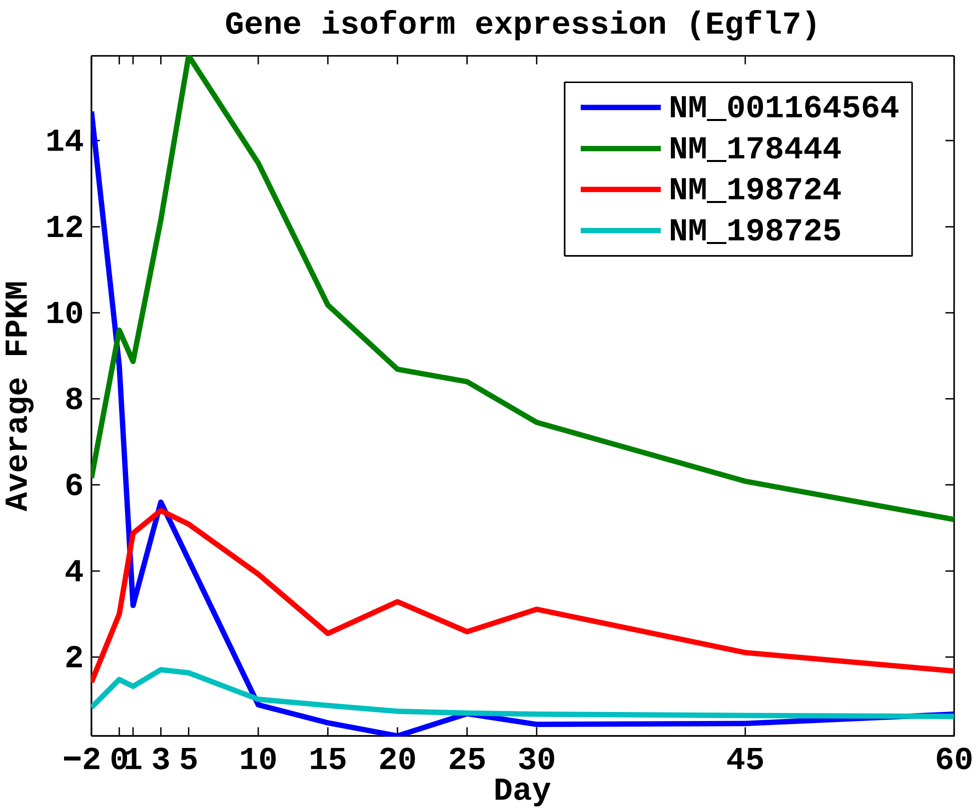

Gene isoform expression (Egfl7)
NM_001164564
NM_178444
NM_198724
NM_198725
Average FPKM
14
12
10
8
6
4
2
−2
0
1
3
5
10
15
20
25
30
45
60
Day

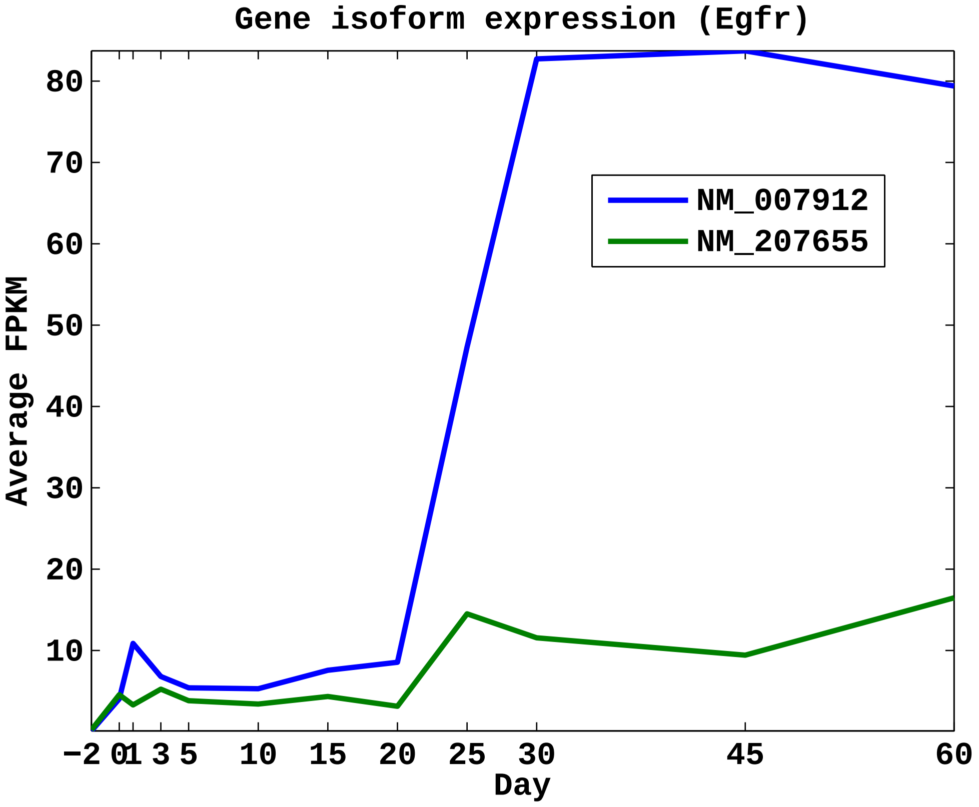

Gene isoform expression (Egfr)
Average FPKM
80
70
60
50
40
30
20
10
NM_007912
NM_207655
−2 0 1 3 5 10 15 20 25 30 45 60
Day

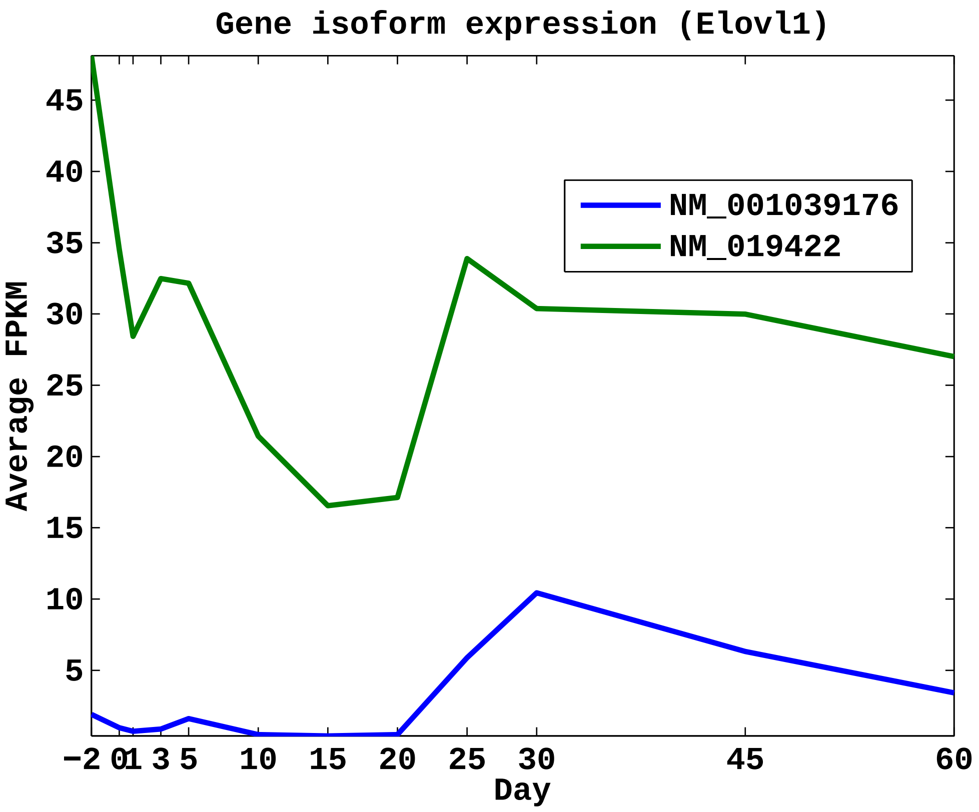

Gene isoform expression (Elovl1)
Average FPKM
Day
NM_001039176
NM_019422
45
40
35
30
25
20
15
10
5
−2 0 1 3 5 10 15 20 25 30 45 60

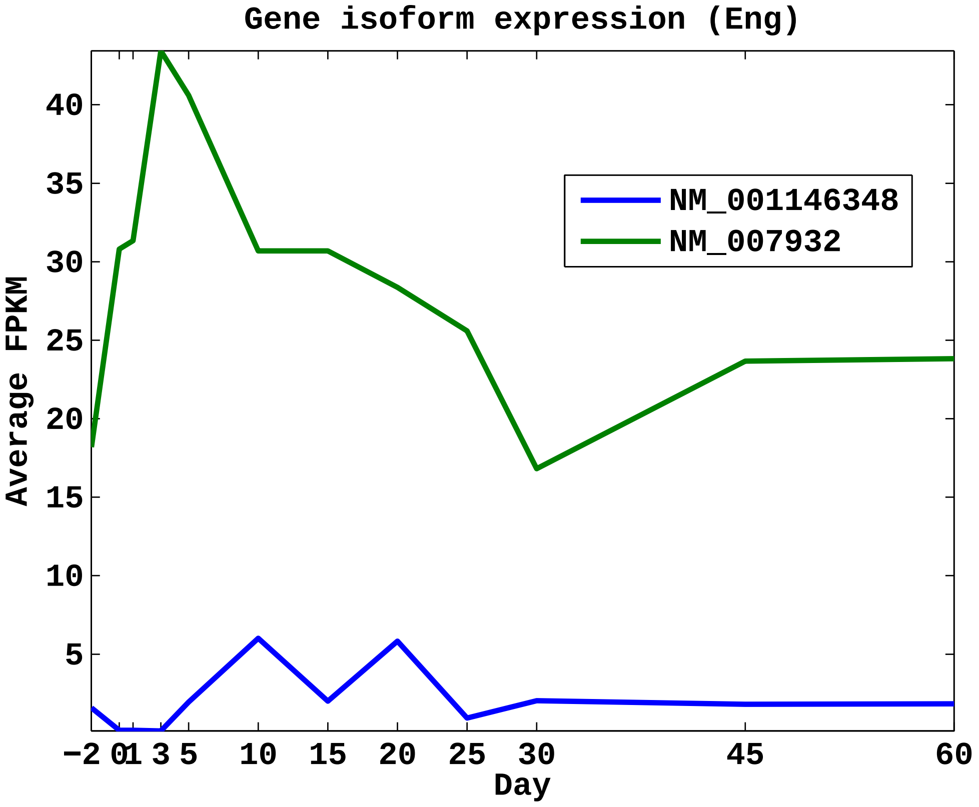

Gene isoform expression (Eng)
Average FPKM
40
35
30
25
20
15
10
5
−2 0 1 3 5 10 15 20 25 30 45 60
Day
NM_001146348
NM_007932

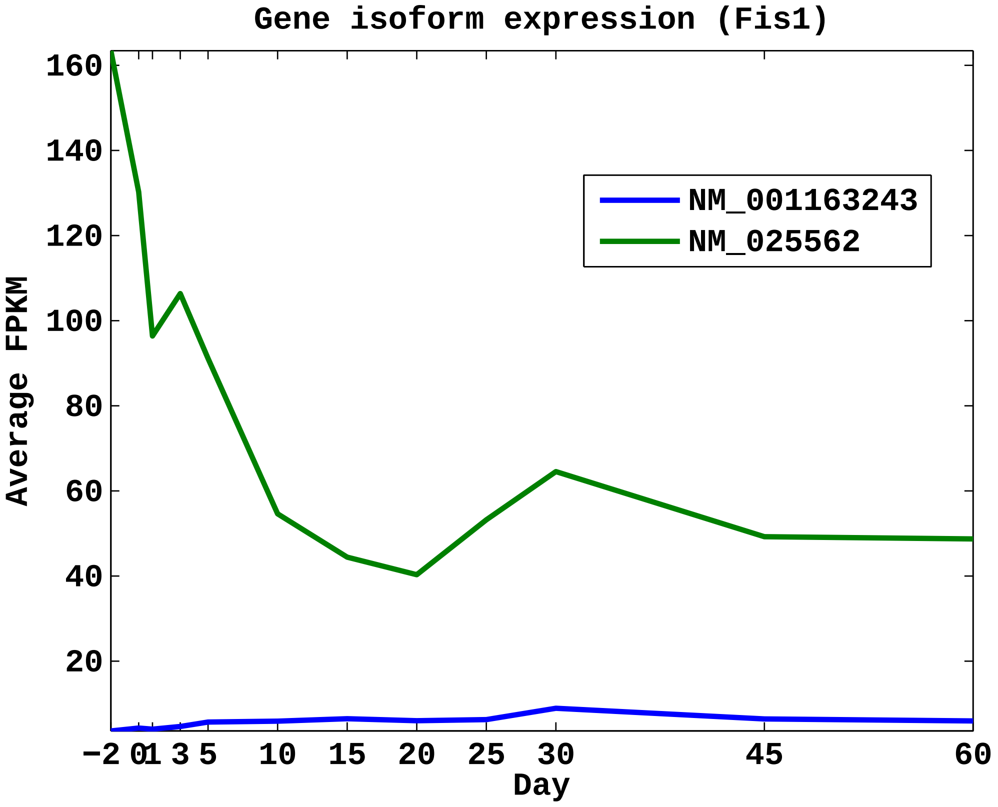
Gene isoform expression (Fis1)
160
140
120
100
80
60
40
20
Average FPKM
−2 0 1 3 5 10 15 20 25 30 45 60
Day
NM_001163243
NM_025562

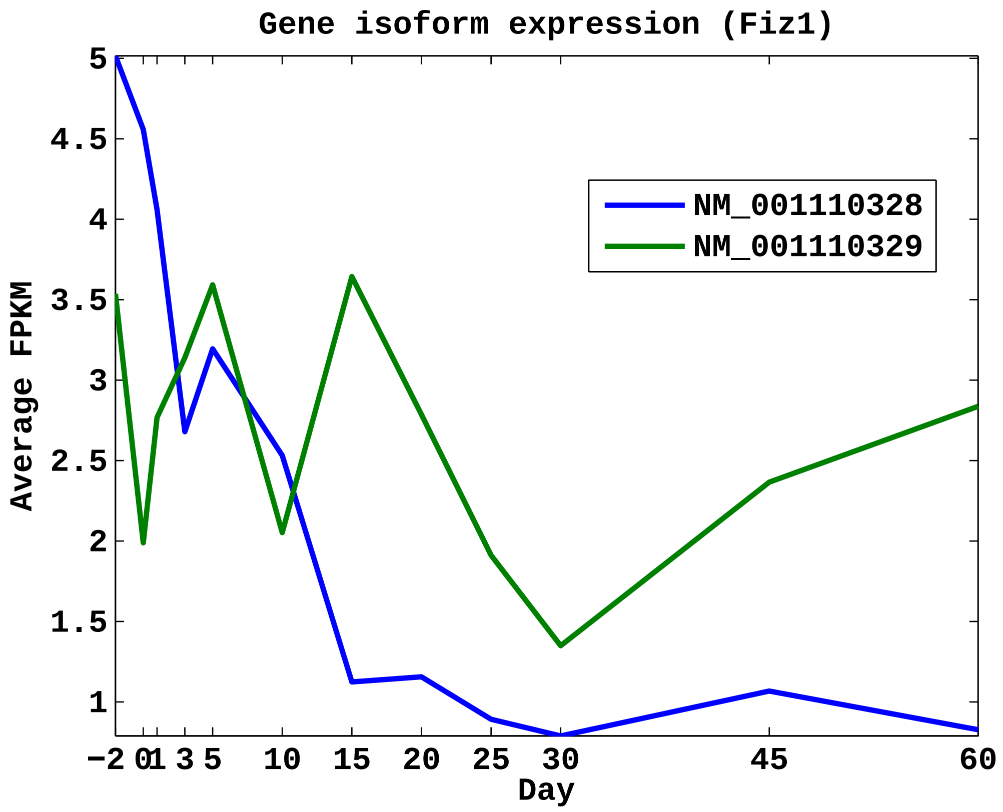
Gene isoform expression (Fiz1)
NM_001110328
NM_001110329
Average FPKM
Day
5
4.5
4
3.5
3
2.5
2
1.5
1
−2 0 1 3 5 10 15 20 25 30 45 60

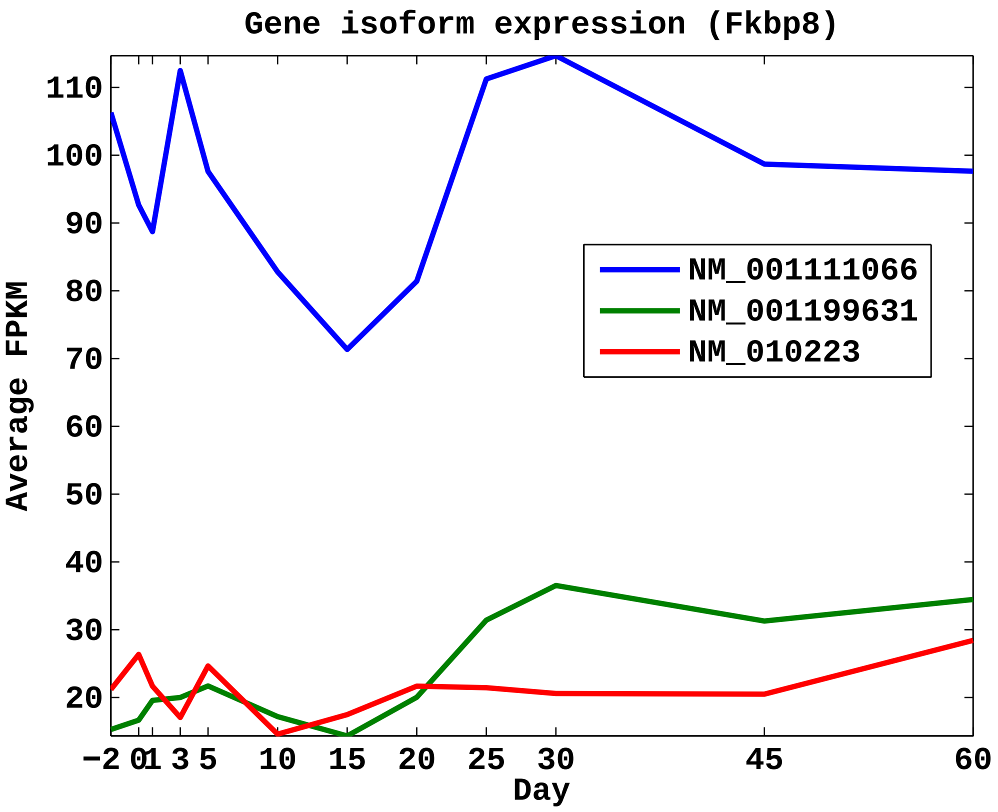

Gene isoform expression (Fkbp8)
Average FPKM
110
100
90
80
70
60
50
40
30
20
−2 0 1 3 5 10 15 20 25 30 45 60
Day
NM_001111066
NM_001199631
NM_010223

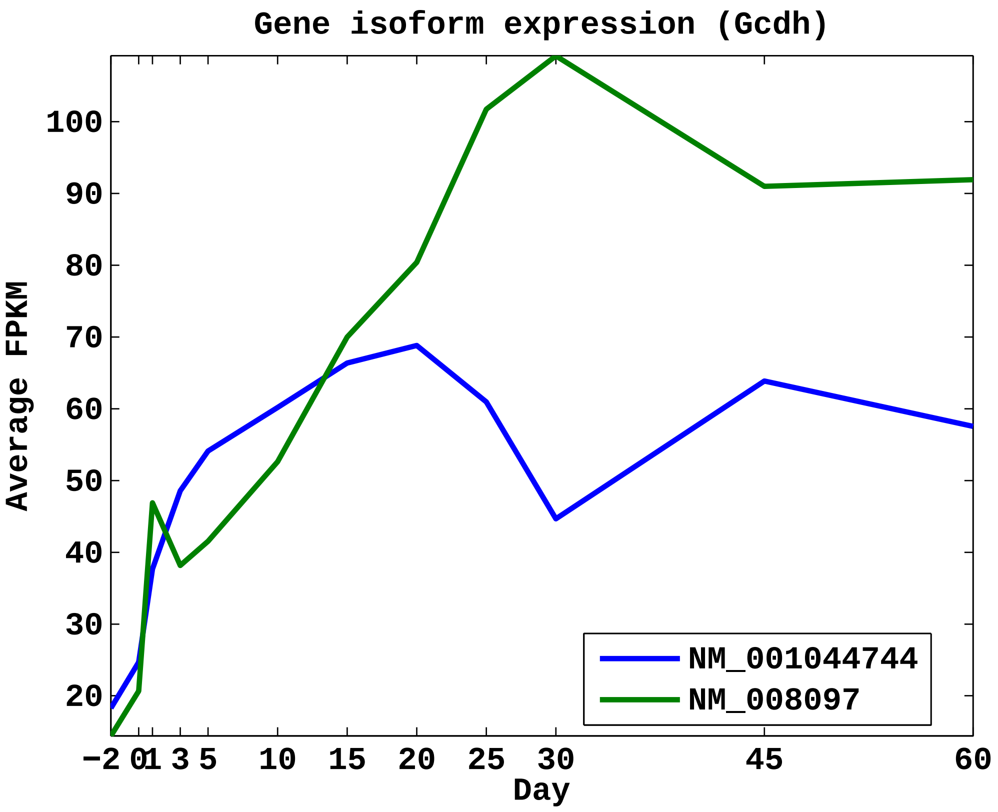

Gene isoform expression (Gcdh)
Average FPKM
Day
100
90
80
70
60
50
40
30
20
−2
0
1
3
5
10
15
20
25
30
45
60
NM_001044744
NM_008097

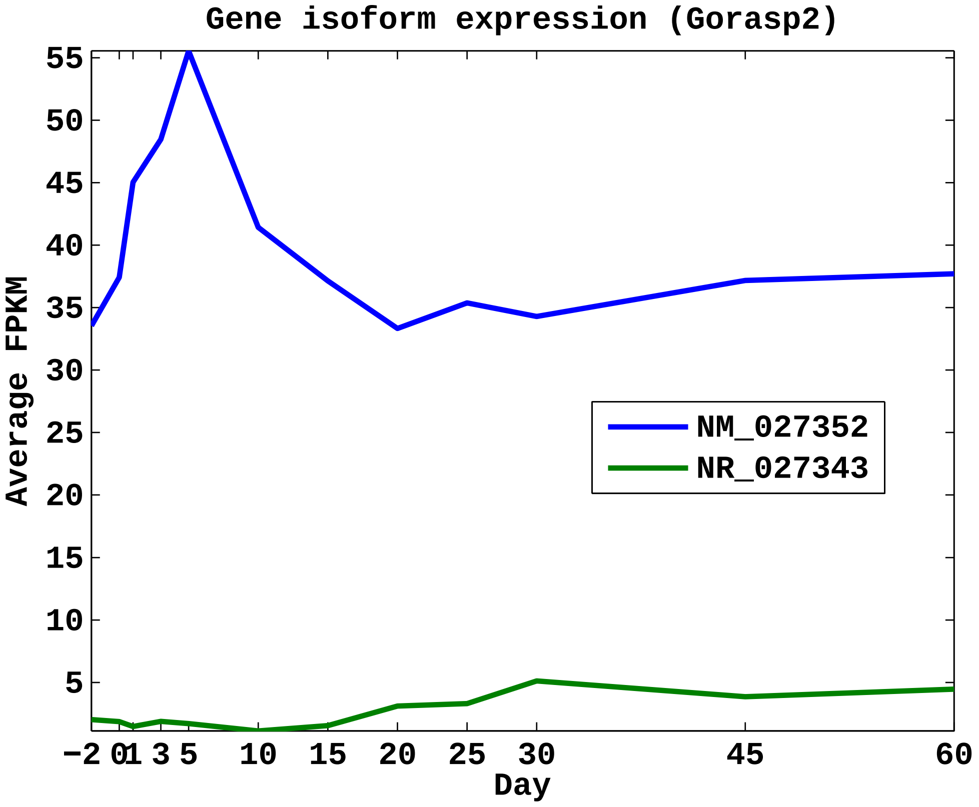
Gene isoform expression (Gorasp2)
Average FPKM
55
50
45
40
35
30
25
20
15
10
5
−2 0 1 3 5 10 15 20 25 30 45 60
Day
NM_027352
NR_027343

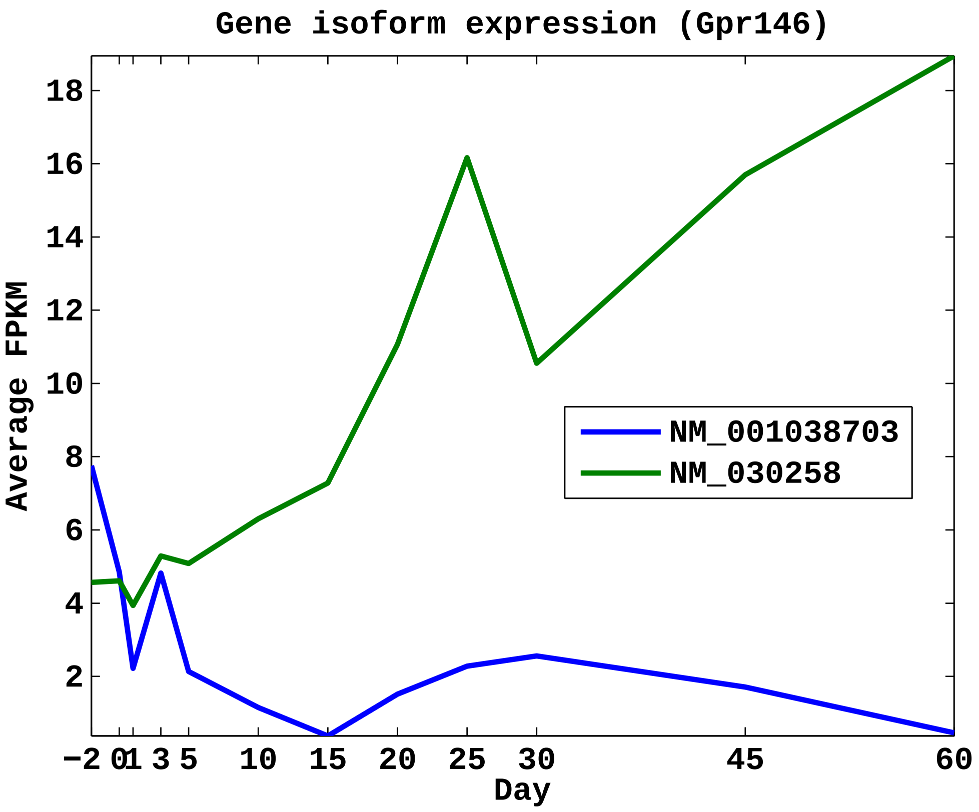
Gene isoform expression (Gpr146)
Average FPKM
18
16
14
12
10
8
6
4
2
−2
0
1
3
5
10
15
20
25
30
45
60
Day
NM_001038703
NM_030258

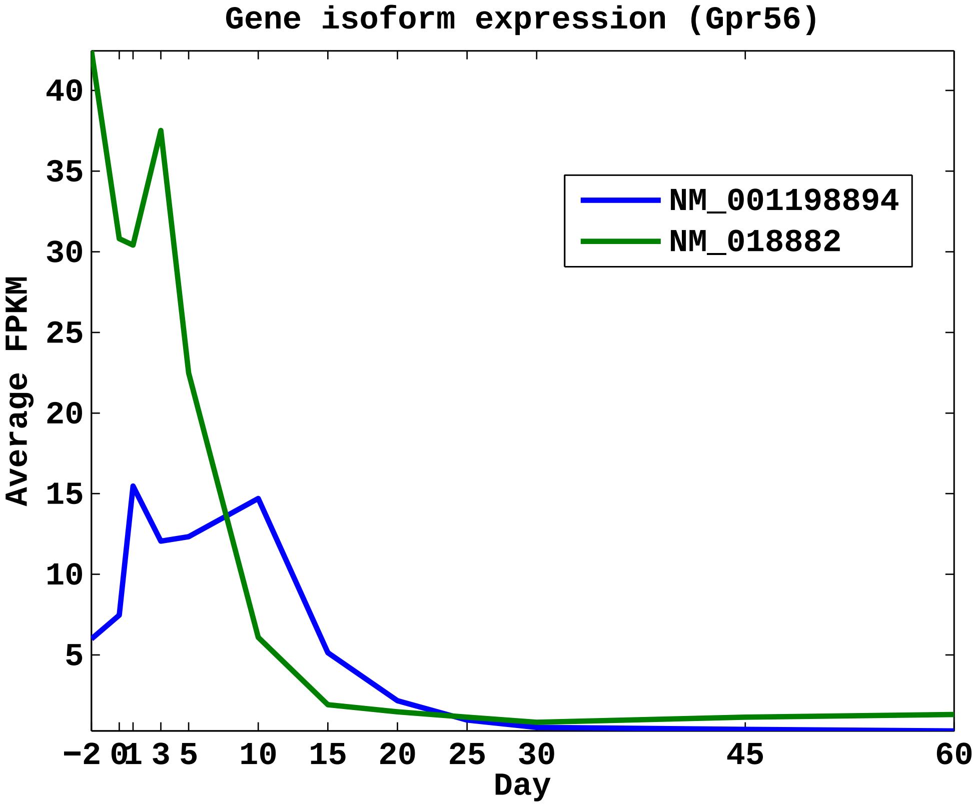
Gene isoform expression (Gpr56)
Average FPKM
40
35
30
25
20
15
10
5
NM_001198894
NM_018882
−2 0 1 3 5 10 15 20 25 30 45 60
Day

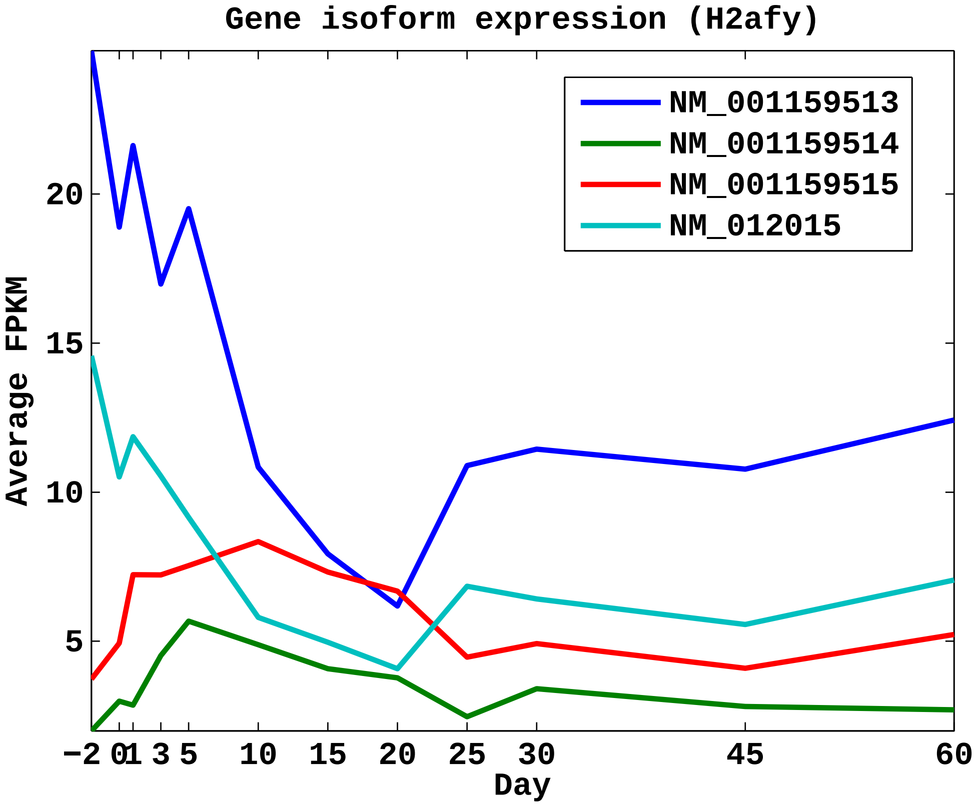
Gene isoform expression (H2afy)
NM_001159513
NM_001159514
NM_001159515
NM_012015
Average FPKM
20
15
10
5
−2
0
1
3
5
10
15
20
25
30
45
60
Day

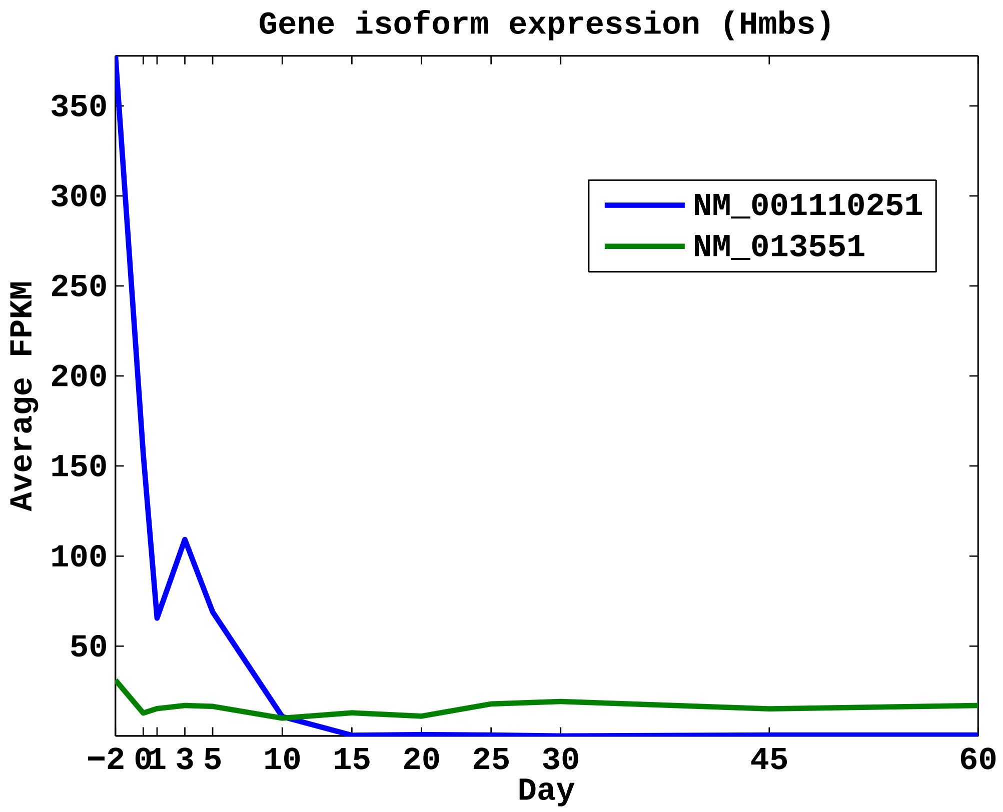
Gene isoform expression (Hmbs)
Average FPKM
350
300
250
200
150
100
50
NM_001110251
NM_013551
−2 0 1 3 5 10 15 20 25 30 45 60
Day

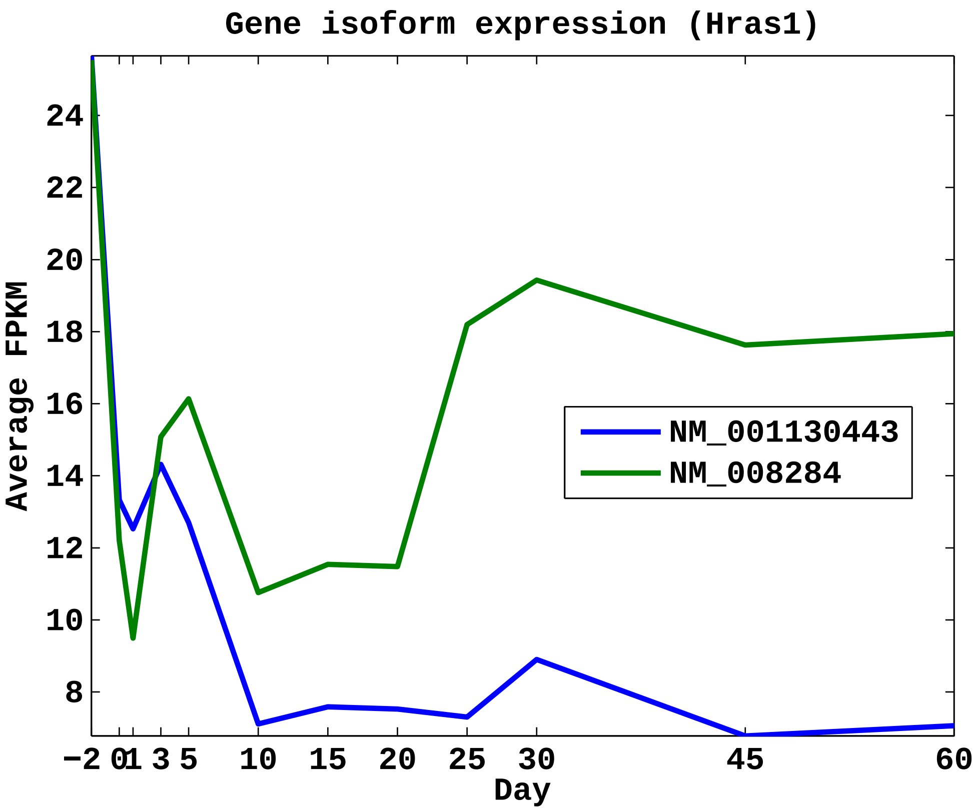
Gene isoform expression (Hras1)
Average FPKM
24
22
20
18
16
14
12
10
8
−2
0
1
3
5
10
15
20
25
30
45
60
Day
NM_001130443
NM_008284

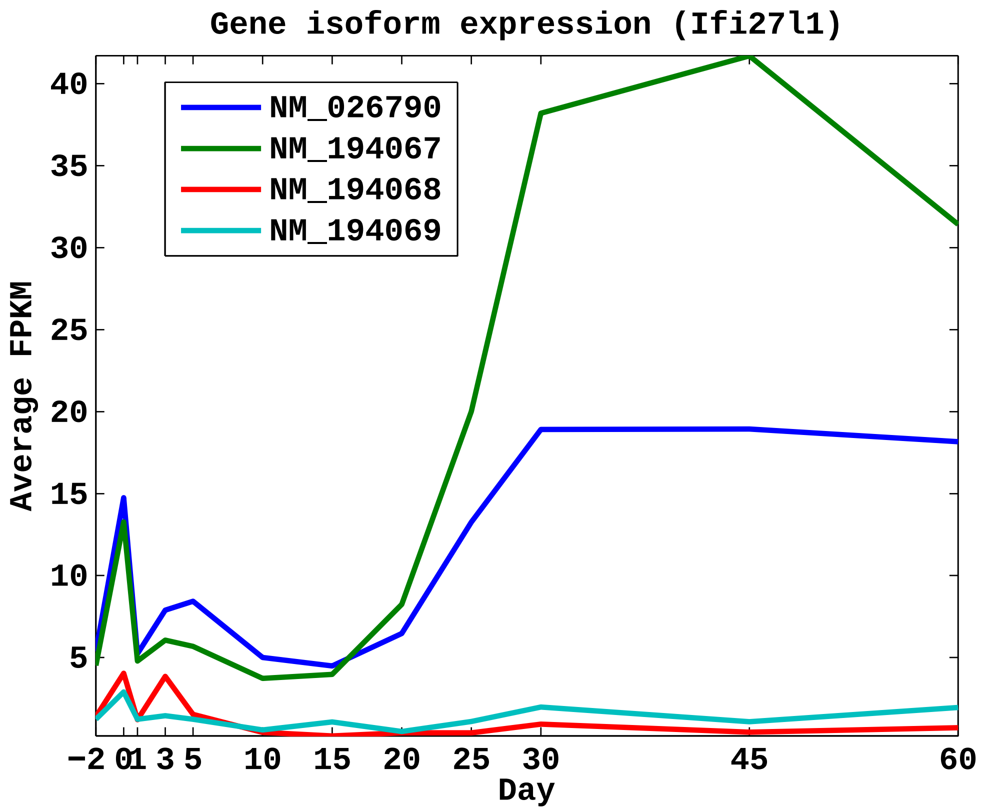

Gene isoform expression (Ifi27l1)
NM_026790
NM_194067
NM_194068
NM_194069
Average FPKM
40
35
30
25
20
15
10
5
−2
0
1
3
5
10
15
20
25
30
45
60
Day

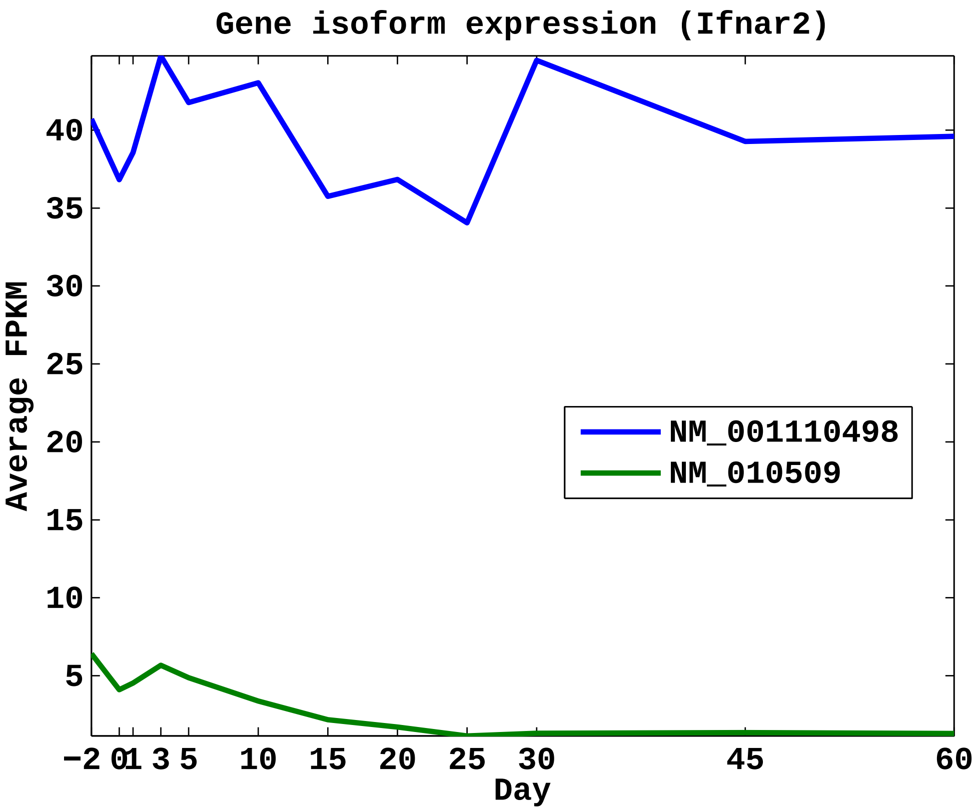

Gene isoform expression (Ifnar2)
Average FPKM
40
35
30
25
20
15
10
5
−2
0
1
3
5
10
15
20
25
30
45
60
Day
NM_001110498
NM_010509

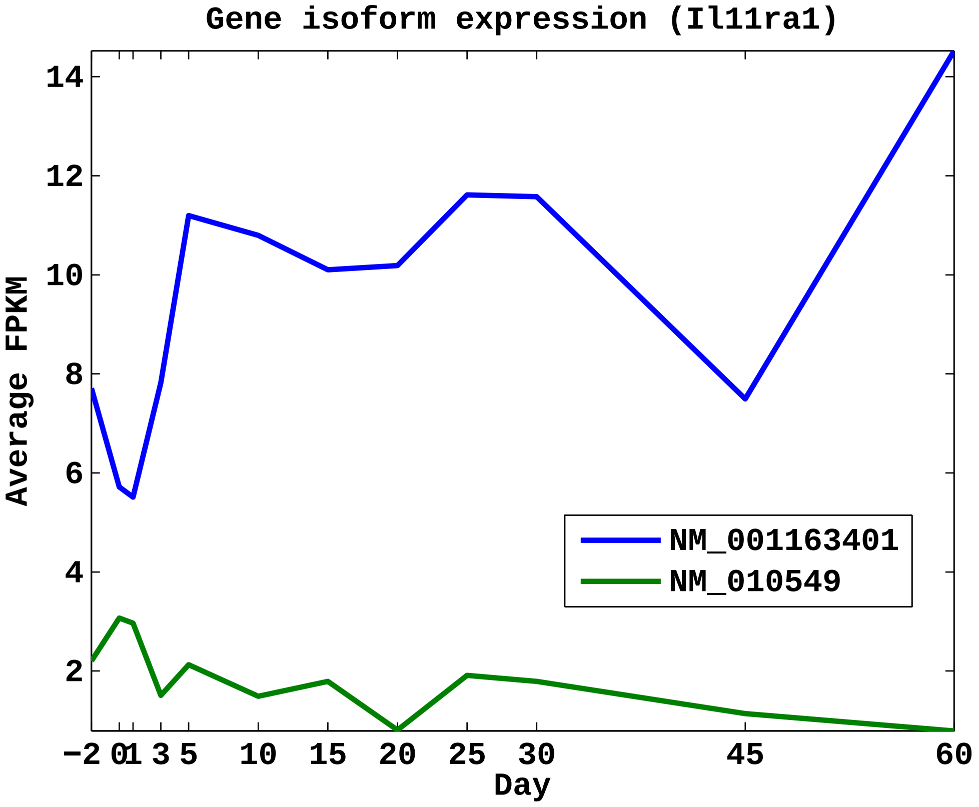

Gene isoform expression (Il11ra1)
Average FPKM
14
12
10
8
6
4
2
−2
0
1
3
5
10
15
20
25
30
45
60
Day
NM_001163401
NM_010549

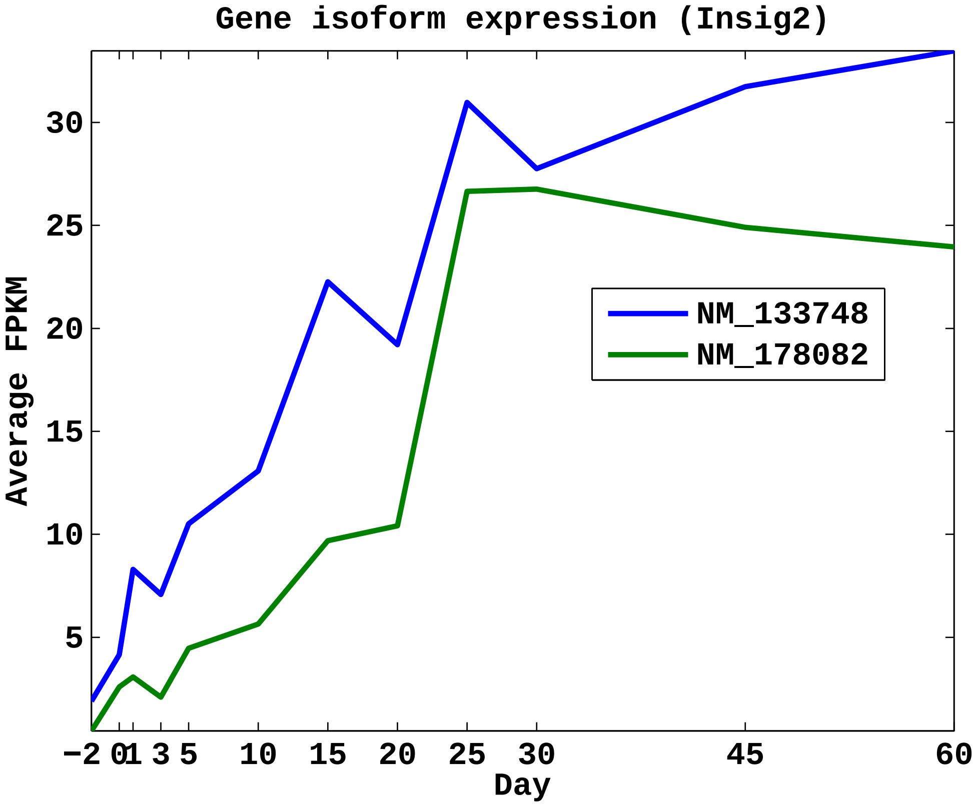
Gene isoform expression (Insig2)
Average FPKM
30
25
20
15
10
5
−2
0
1
3
5
10
15
20
25
30
45
60
Day
NM_133748
NM_178082

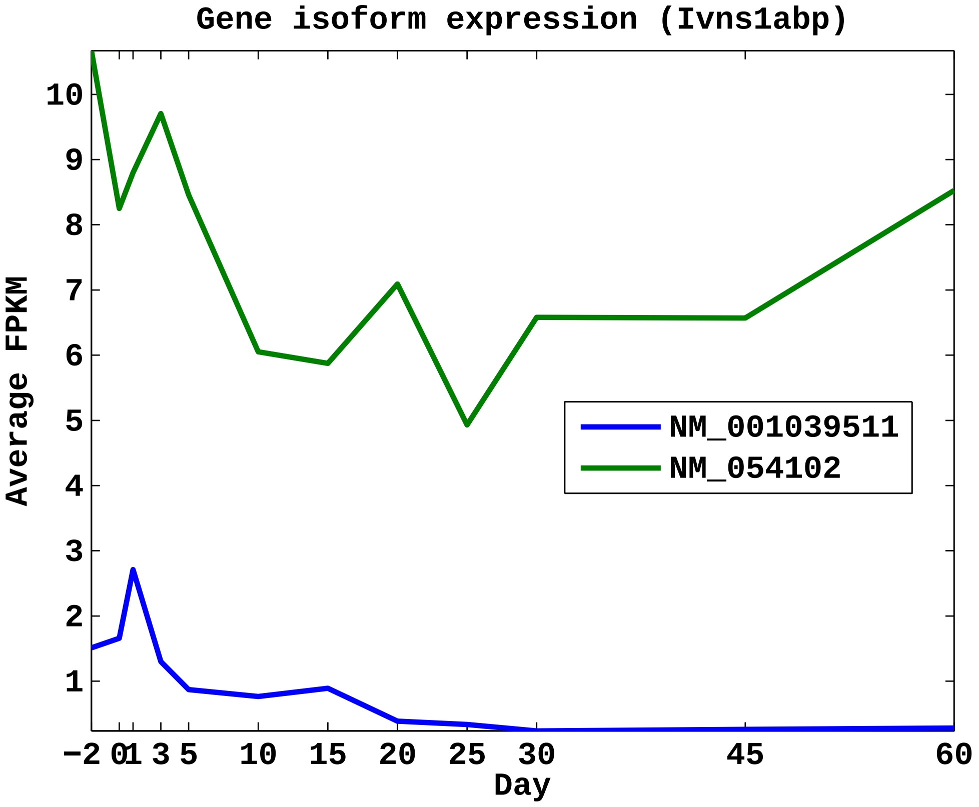
Gene isoform expression (Ivns1abp)
Average FPKM
Day
NM_001039511
NM_054102
10
9
8
7
6
5
4
3
2
1
−2
0
1
3
5
10
15
20
25
30
45
60

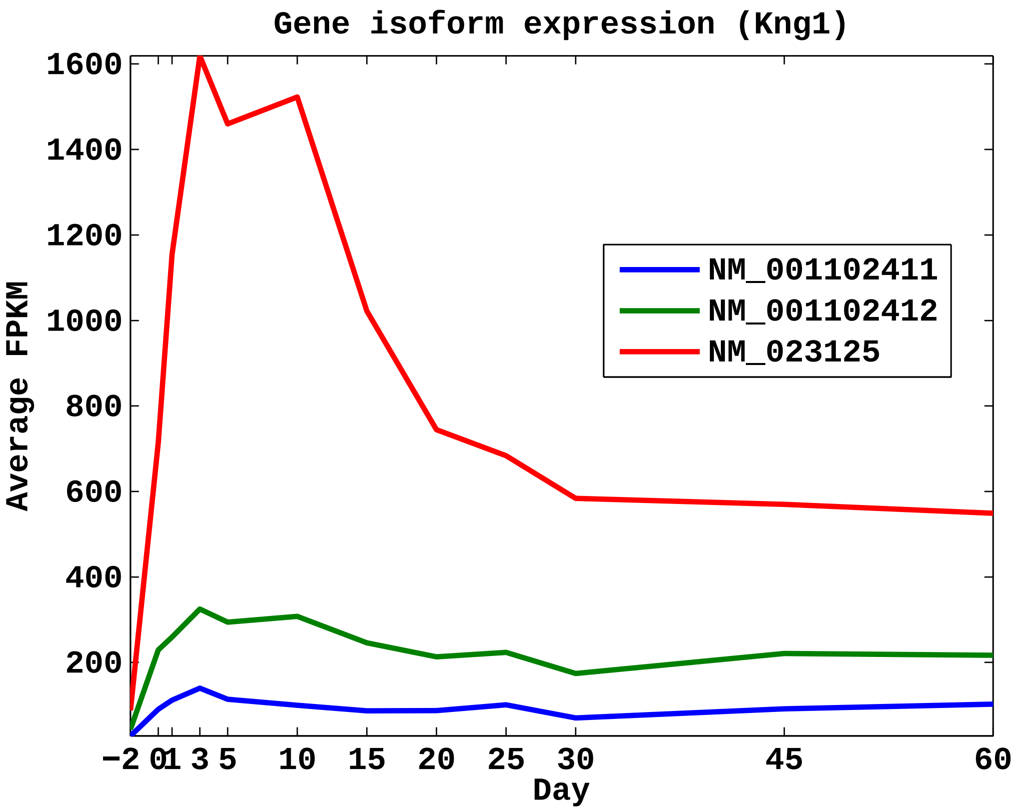
Gene isoform expression (Kng1)
Average FPKM
1600
1400
1200
1000
800
600
400
200
−2
0
1
3
5
10
15
20
25
30
45
60
Day
NM_001102411
NM_001102412
NM_023125

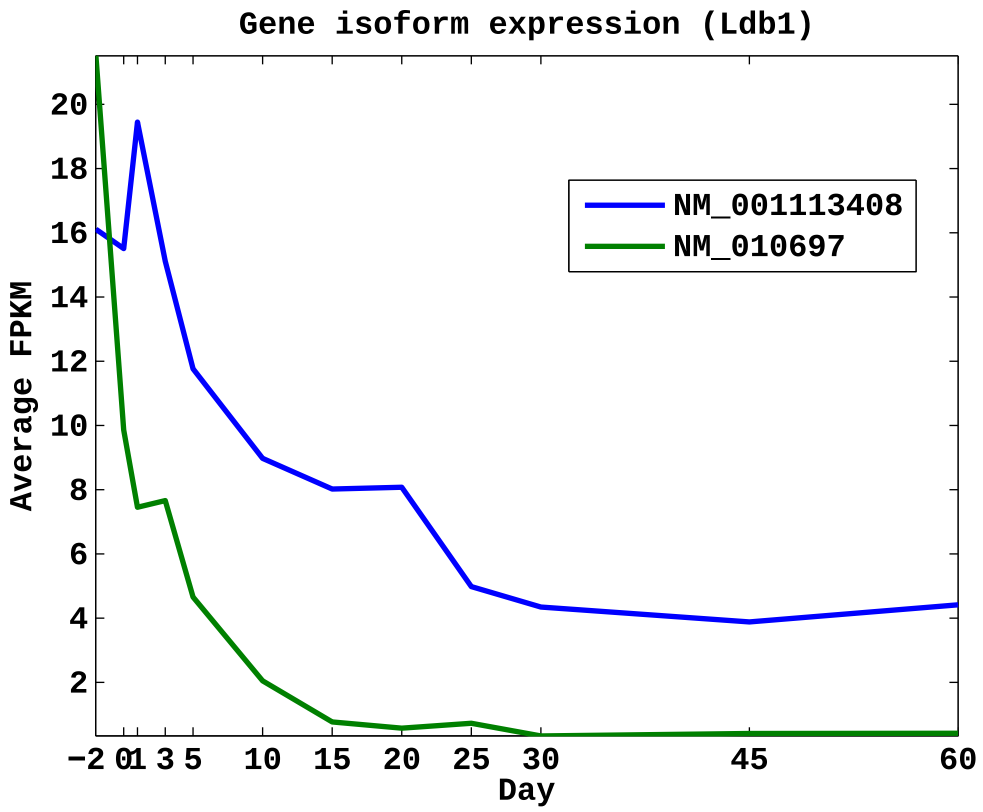

Gene isoform expression (Ldb1)
Average FPKM
20
18
16
14
12
10
8
6
4
2
−2 0 1 3 5 10 15 20 25 30 45 60
Day
NM_001113408
NM_010697

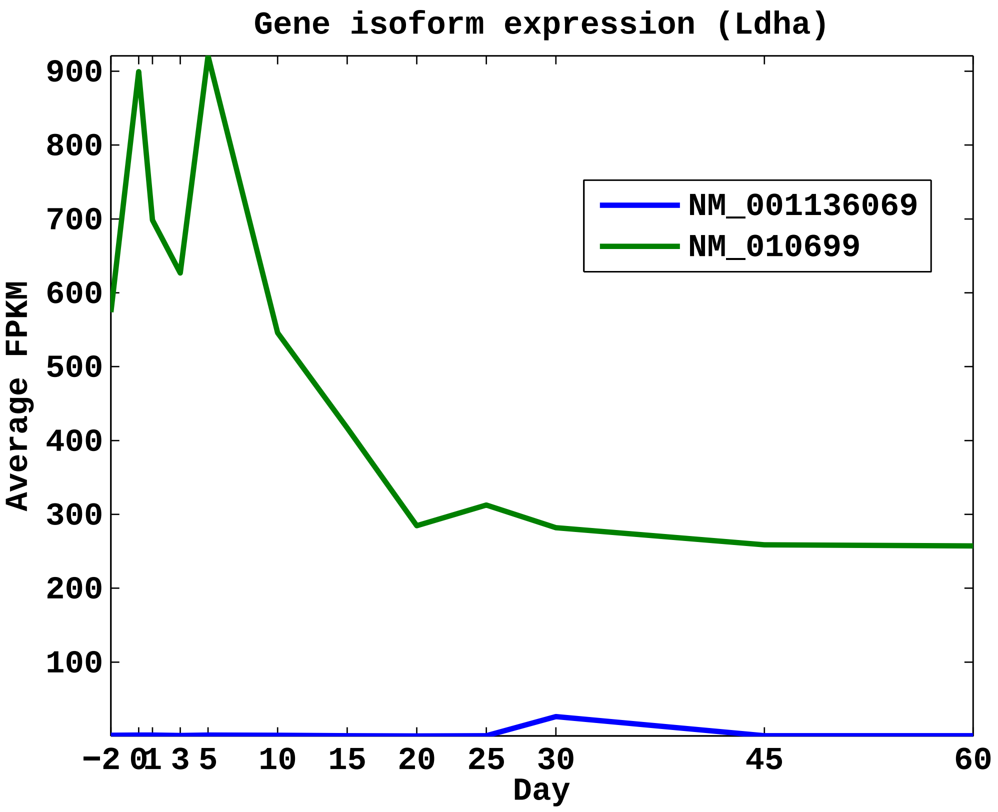

Gene isoform expression (Ldha)
Average FPKM
900
800
700
600
500
400
300
200
100
−2
0
1
3
5
10
15
20
25
30
45
60
Day
NM_001136069
NM_010699

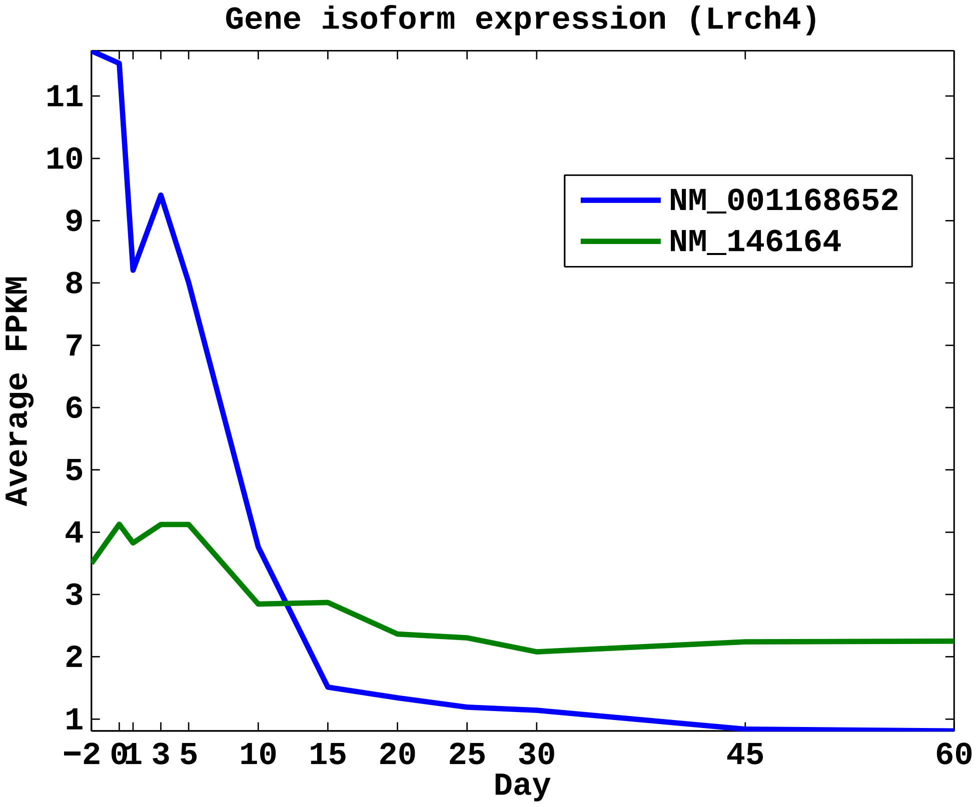

Gene isoform expression (Lrch4)
NM_001168652
NM_146164
Average FPKM
Day
11
10
9
8
7
6
5
4
3
2
1
−2
0
1
3
5
10
15
20
25
30
45
60

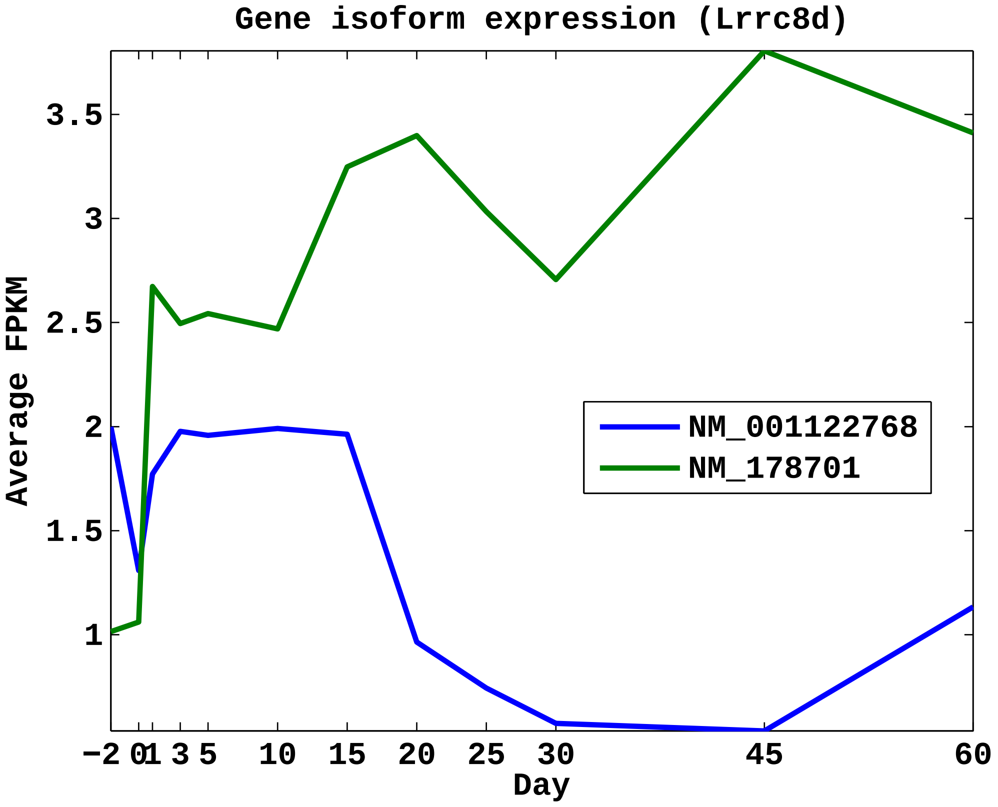

Gene isoform expression (Lrrc8d)
Average FPKM
3.5
3
2.5
2
1.5
1
−2 0 1 3 5 10 15 20 25 30 45 60
Day
NM_001122768
NM_178701

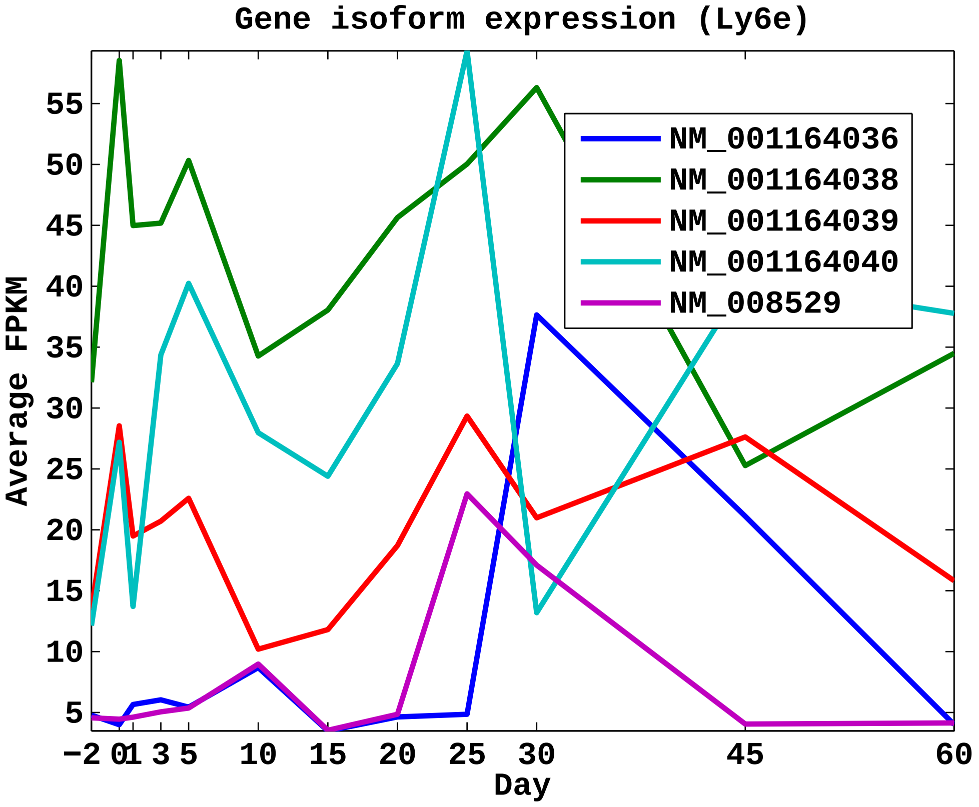
Gene isoform expression (Ly6e)
Average FPKM
55
50
45
40
35
30
25
20
15
10
5
−2
0
1
3
5
10
15
20
25
30
45
60
Day
NM_001164036
NM_001164038
NM_001164039
NM_001164040
NM_008529

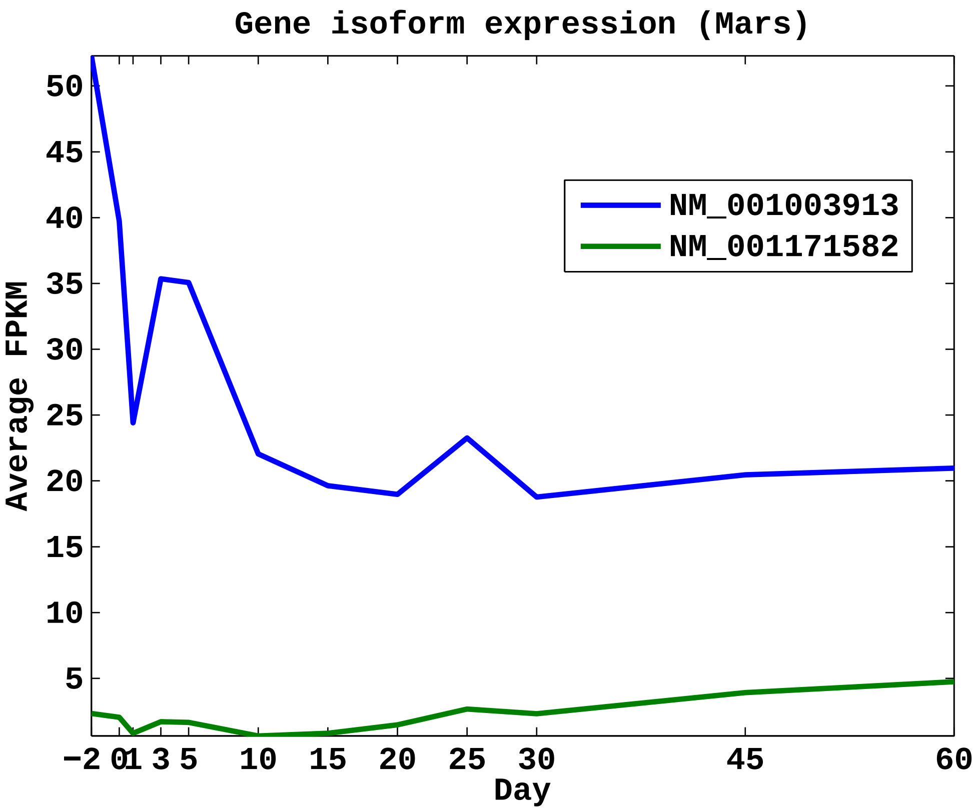

Gene isoform expression (Mars)
NM_001003913
NM_001171582
Average FPKM
50
45
40
35
30
25
20
15
10
5
−2 0 1 3 5 10 15 20 25 30 45 60
Day

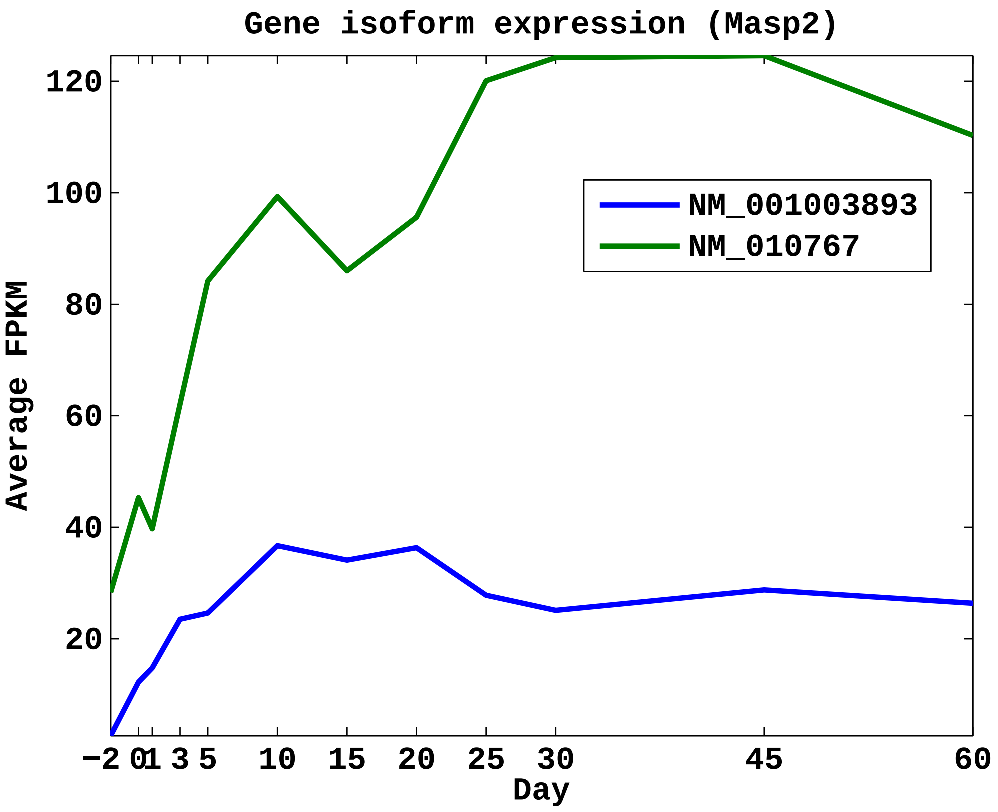

Gene isoform expression (Masp2)
NM_001003893
NM_010767
Average FPKM
120
100
80
60
40
20
−2 0 1 3 5 10 15 20 25 30 45 60
Day

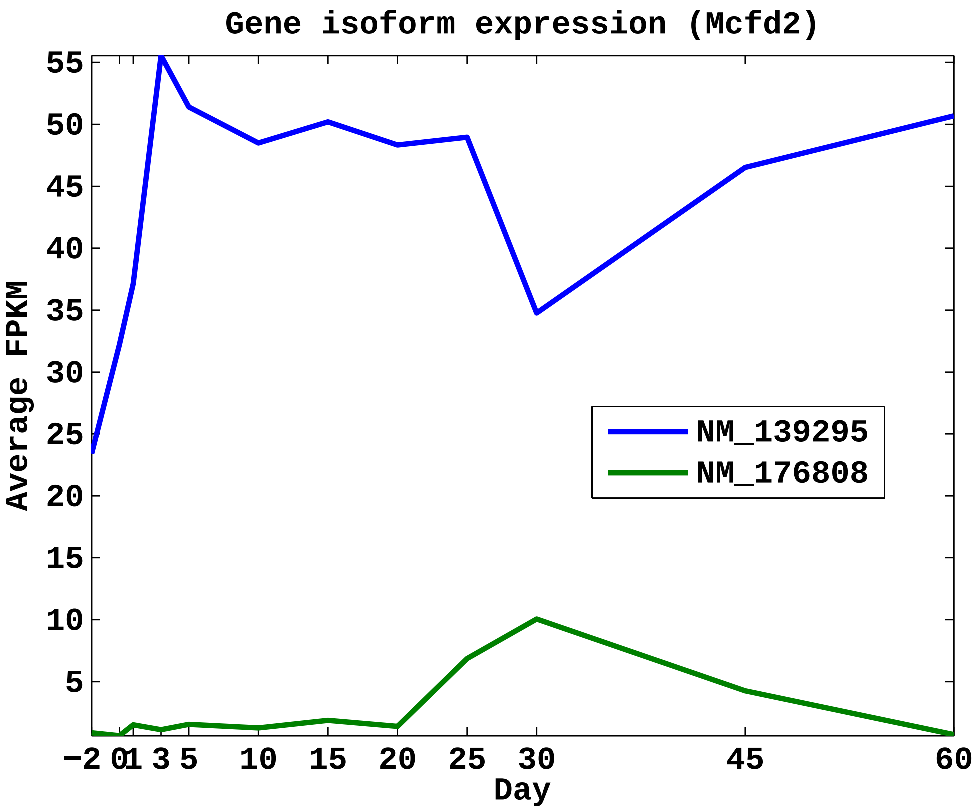

Gene isoform expression (Mcfd2)
Average FPKM
55
50
45
40
35
30
25
20
15
10
5
−2
0
1
3
5
10
15
20
25
30
45
60
Day
NM_139295
NM_176808

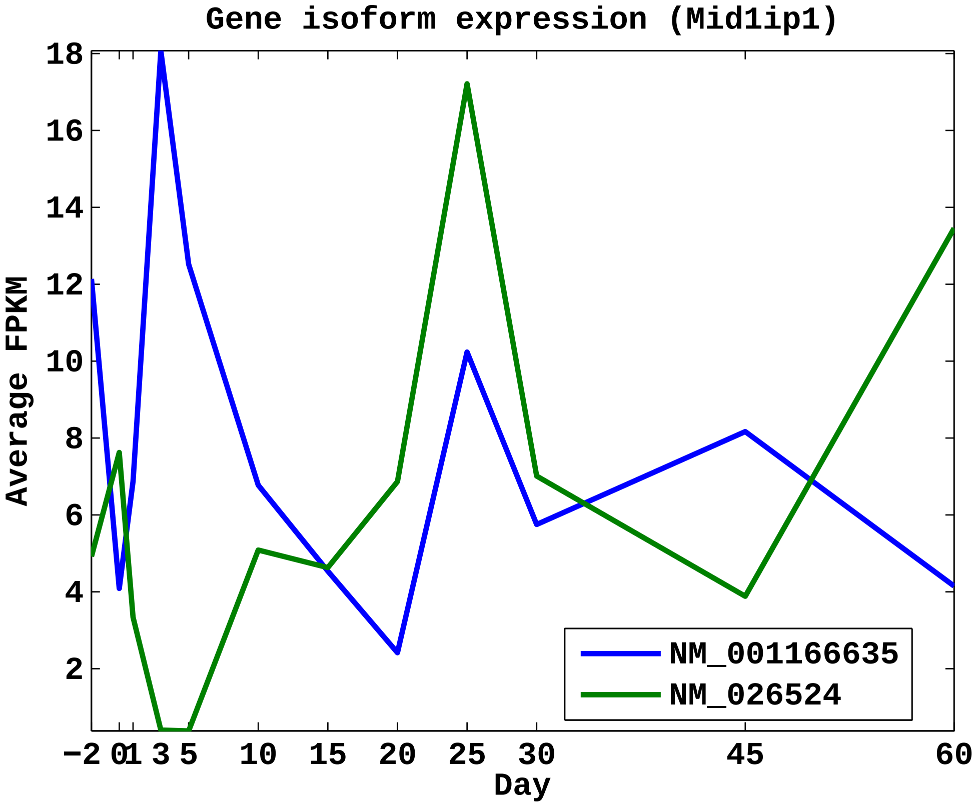
Gene isoform expression (Mid1ip1)
Average FPKM
18
16
14
12
10
8
6
4
2
−2
0
1
3
5
10
15
20
25
30
45
60
Day
NM_001166635
NM_026524

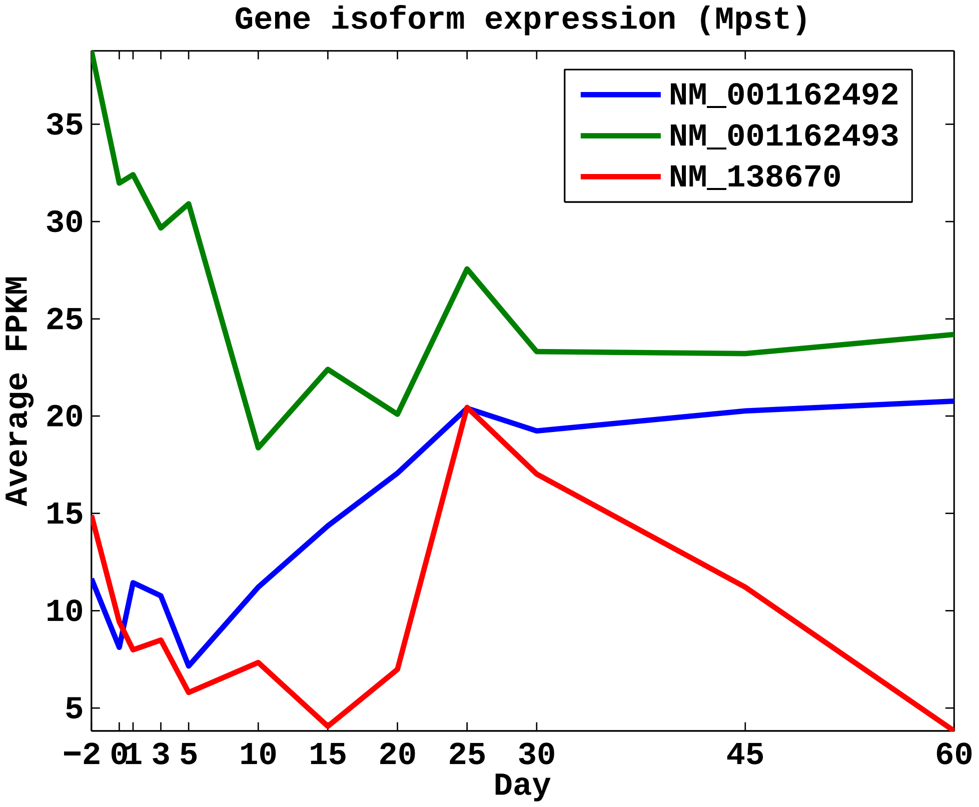
Gene isoform expression (Mpst)
NM_001162492
NM_001162493
NM_138670
35
30
25
20
15
10
5
Average FPKM
−2 0 1 3 5 10 15 20 25 30 45 60
Day

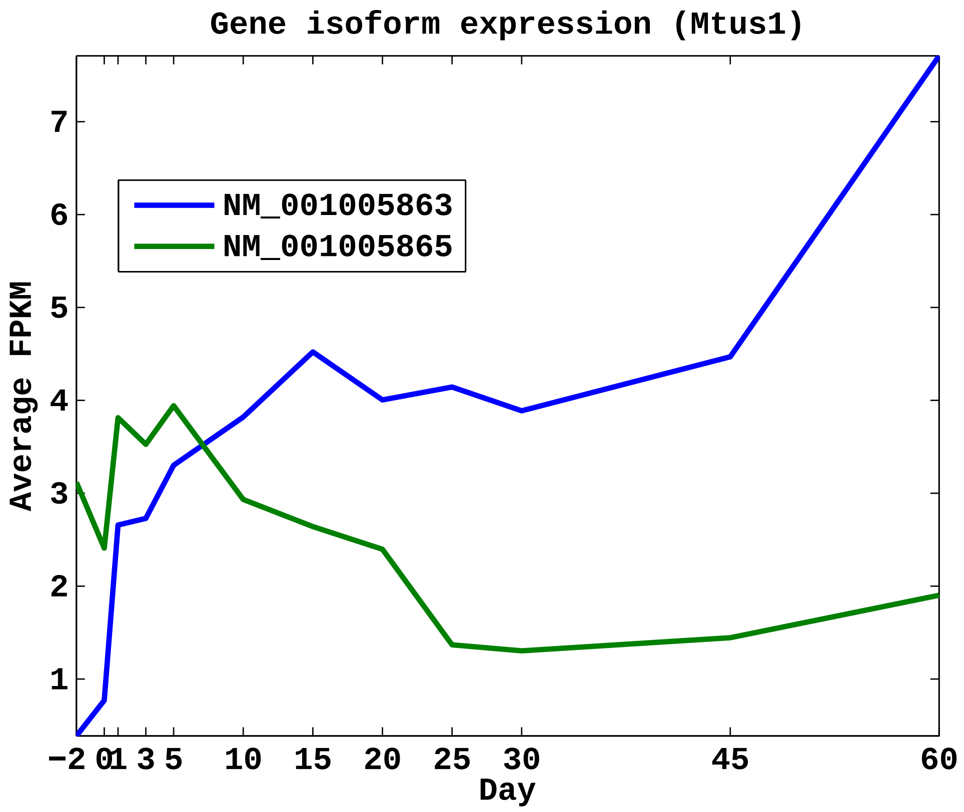
Gene isoform expression (Mtus1)
NM_001005863
NM_001005865
Average FPKM
Day
−2 0 1 3 5 10 15 20 25 30 45 60
1 2 3 4 5 6 7

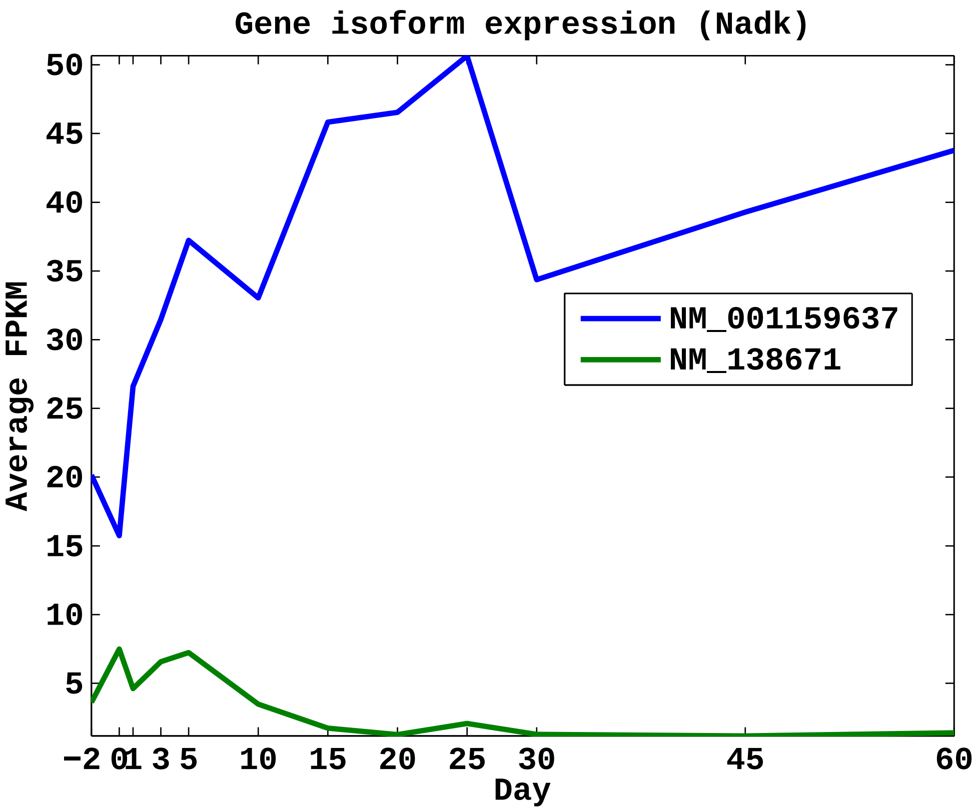

Gene isoform expression (Nadk)
Average FPKM
50
45
40
35
30
25
20
15
10
5
−2
0
1
3
5
10
15
20
25
30
45
60
Day
NM_001159637
NM_138671

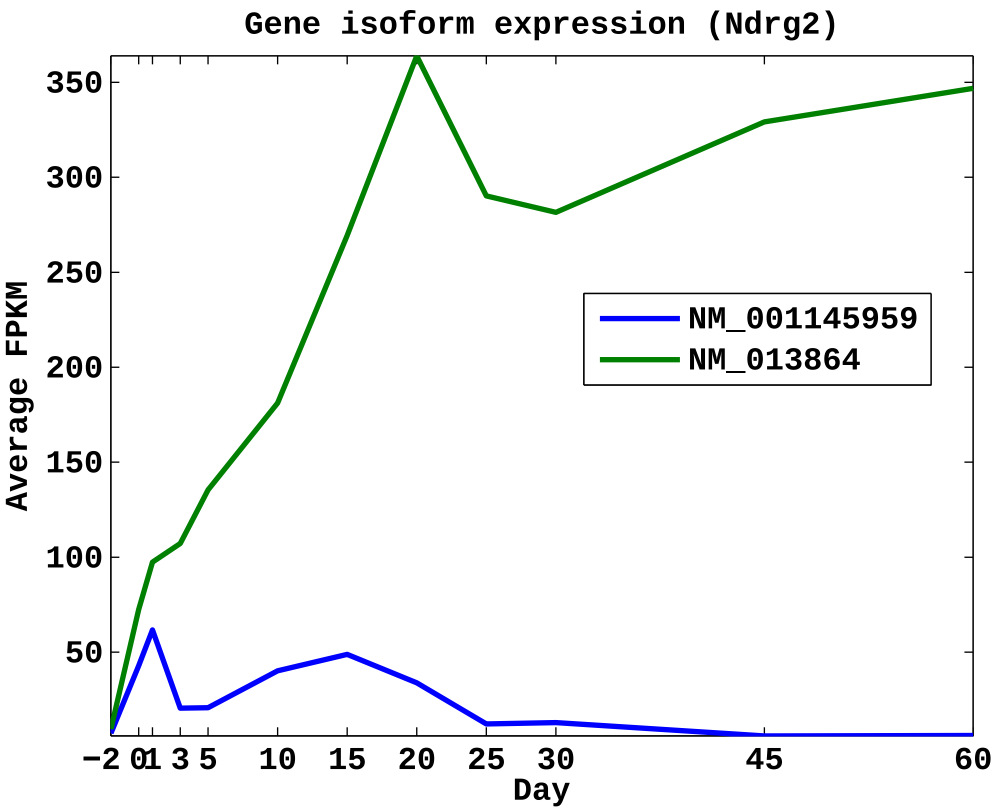

Gene isoform expression (Ndrg2)
Average FPKM
350
300
250
200
150
100
50
−2 0 1 3 5 10 15 20 25 30 45 60
Day
NM_001145959
NM_013864

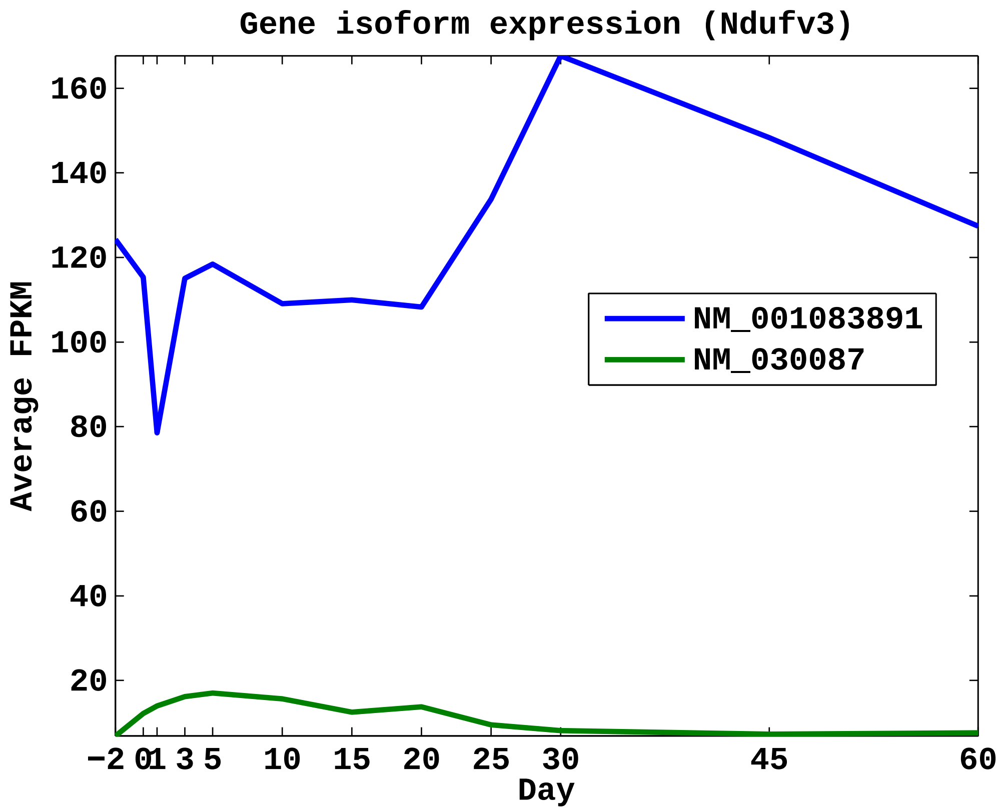

Gene isoform expression (Ndufv3)
Average FPKM
160
140
120
100
80
60
40
20
−2
0
1
3
5
10
15
20
25
30
45
60
Day
NM_001083891
NM_030087

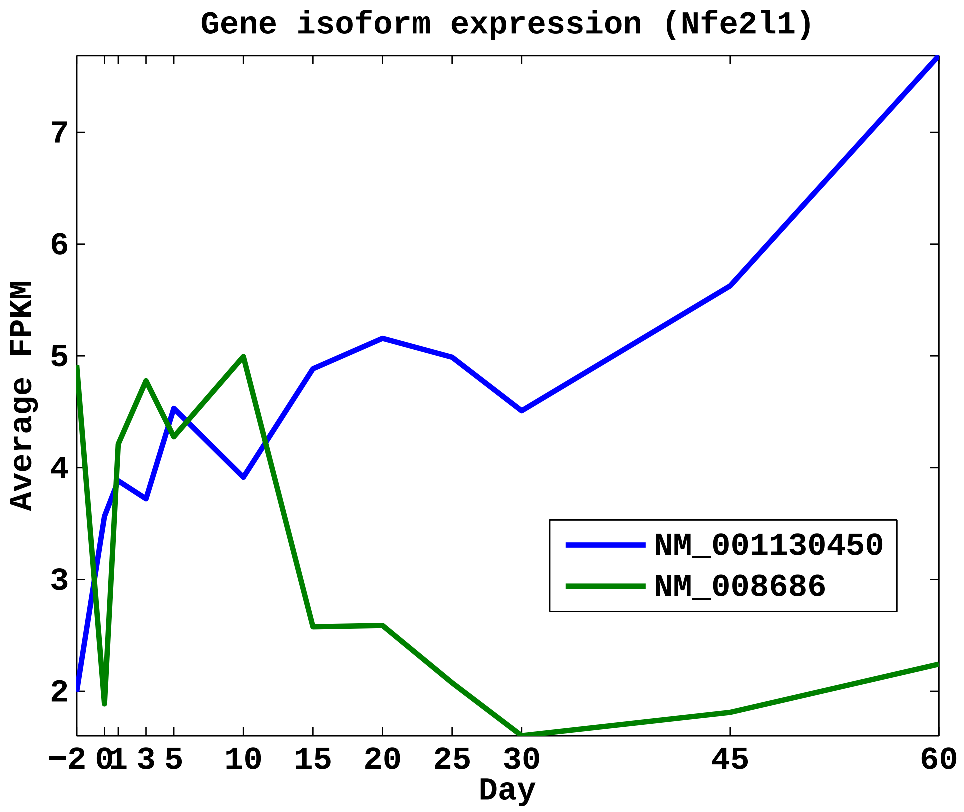
Gene isoform expression (Nfe2l1)
Average FPKM
7
6
5
4
3
2
−2 0 1 3 5 10 15 20 25 30 45 60
Day
NM_001130450
NM_008686

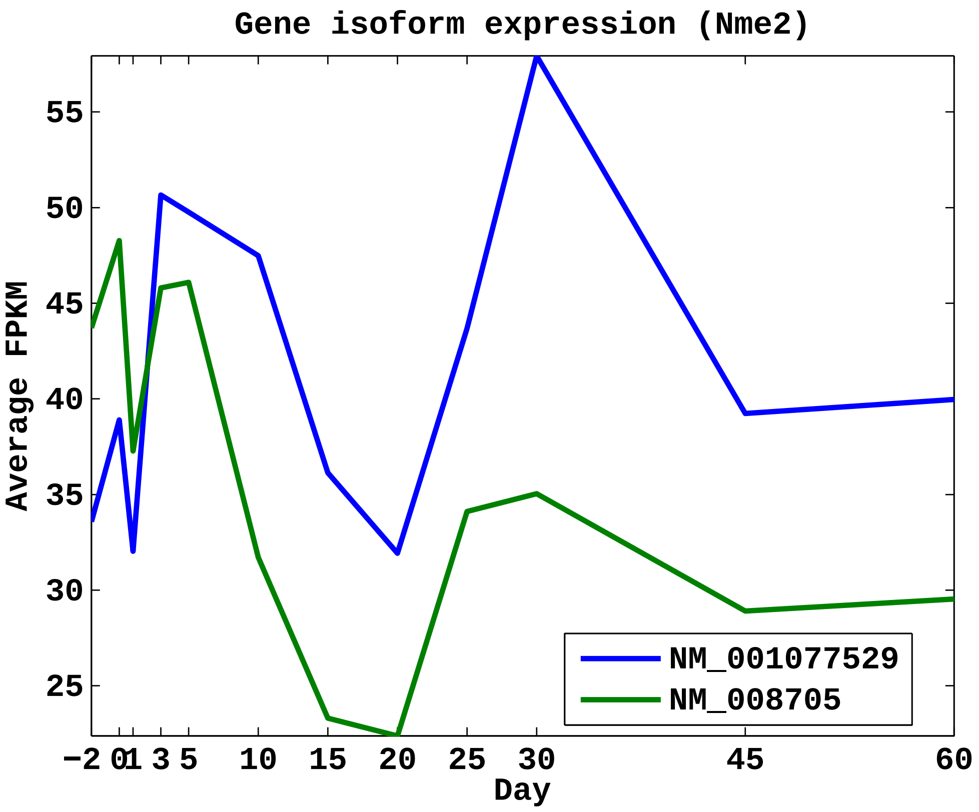

Gene isoform expression (Nme2)
Average FPKM
55
50
45
40
35
30
25
-2
0
1
3
5
10
15
20
25
30
45
60
Day
NM_001077529
NM_008705

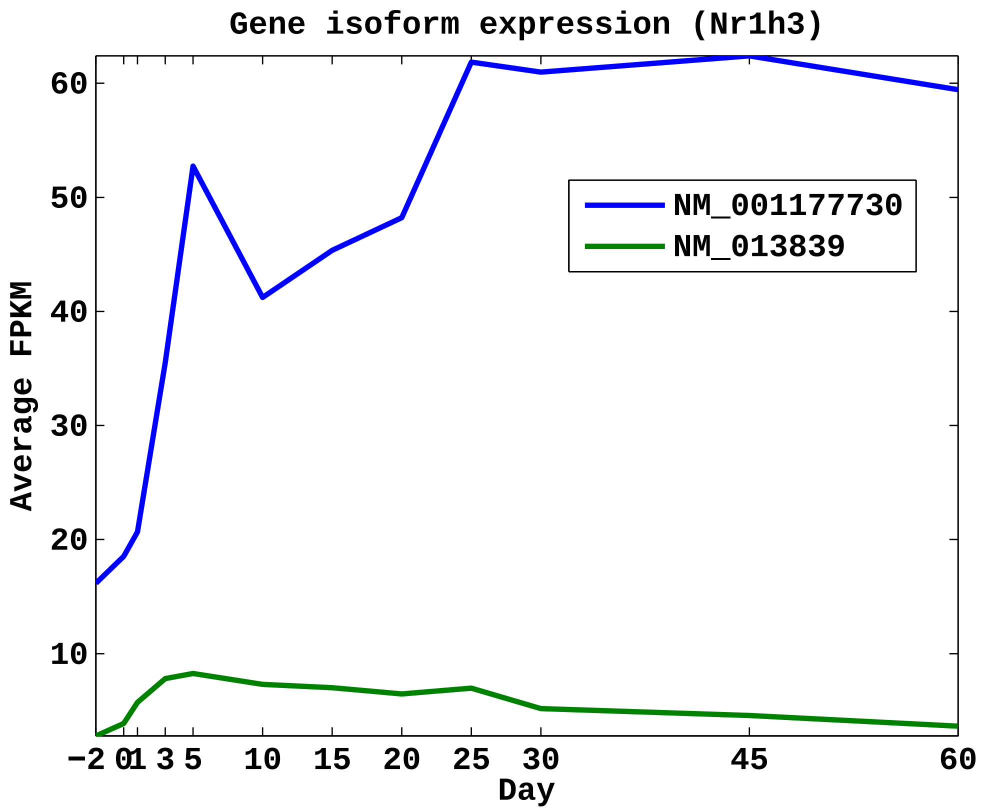

Gene isoform expression (Nr1h3)
Average FPKM
60
50
40
30
20
10
NM_001177730
NM_013839
−2 0 1 3 5 10 15 20 25 30 45 60
Day

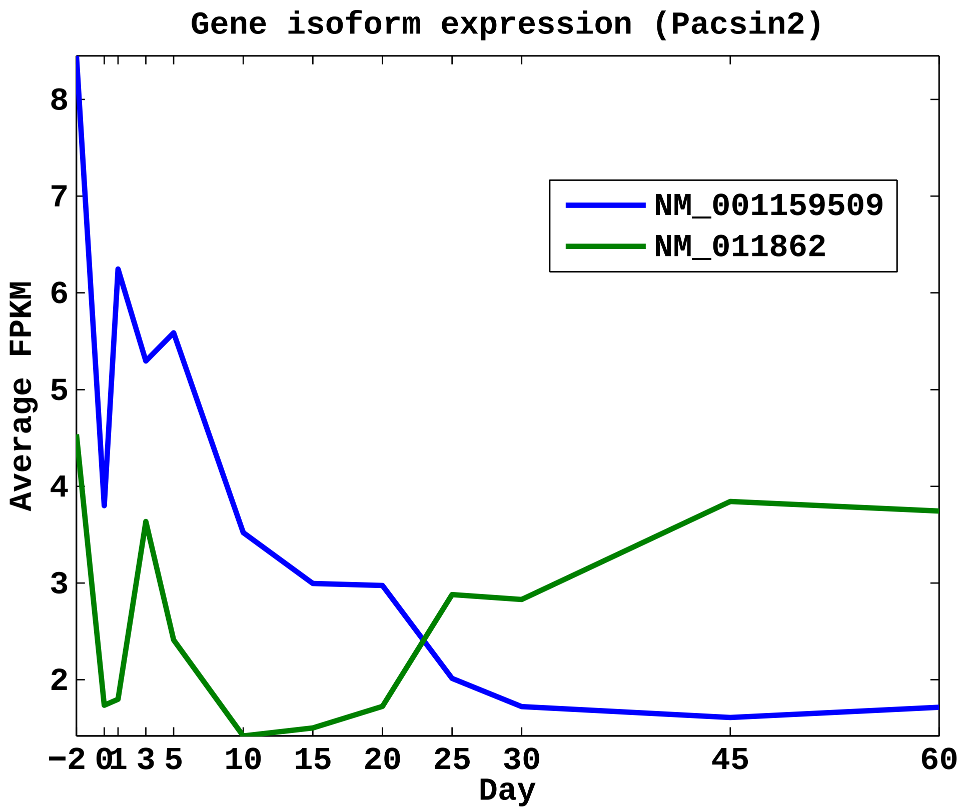
Gene isoform expression (Pacsin2)
NM_001159509
NM_011862
Average FPKM
8
7
6
5
4
3
2
−2
0
1
3
5
10
15
20
25
30
45
60
Day

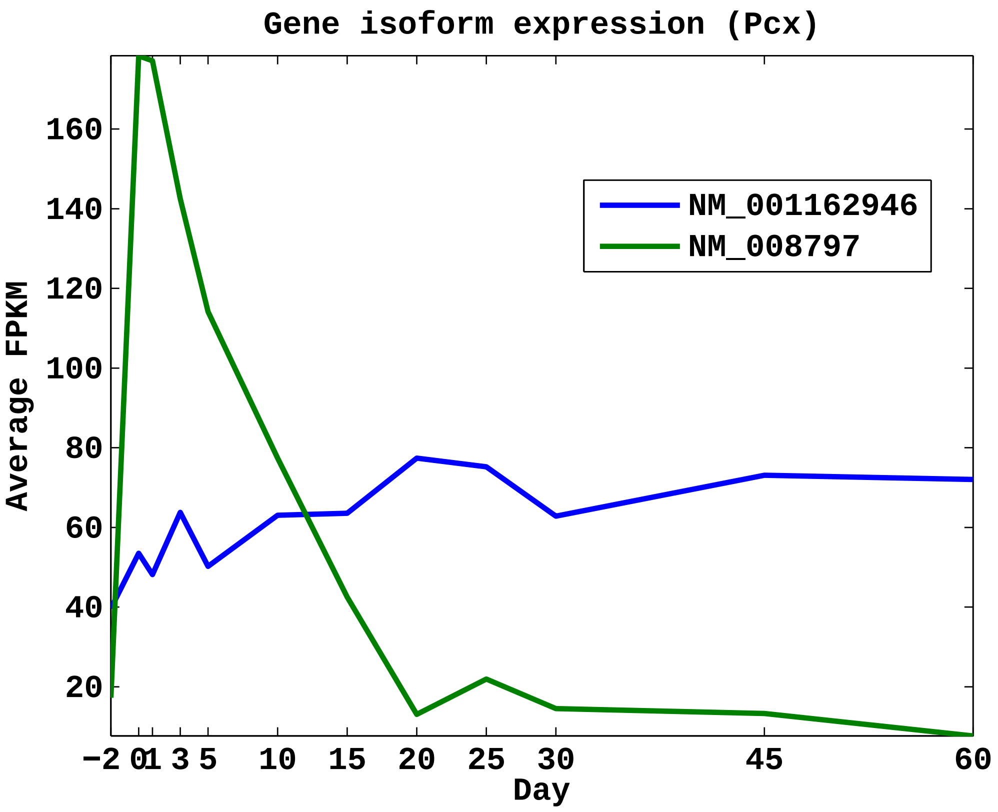
Gene isoform expression (Pcx)
NM_001162946
NM_008797
Average FPKM
160
140
120
100
80
60
40
20
−2 0 1 3 5 10 15 20 25 30 45 60
Day

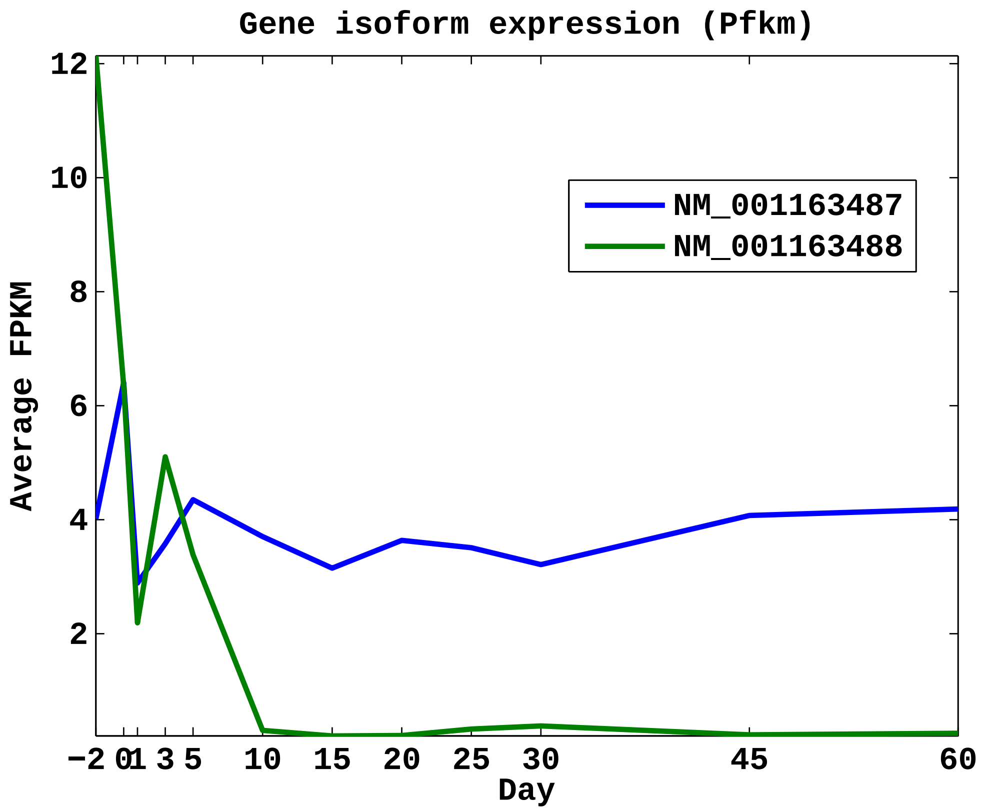

Gene isoform expression (Pfkm)
Average FPKM
Day
NM_001163487
NM_001163488
12
10
8
6
4
2
−2 0 1 3 5 10 15 20 25 30 45 60

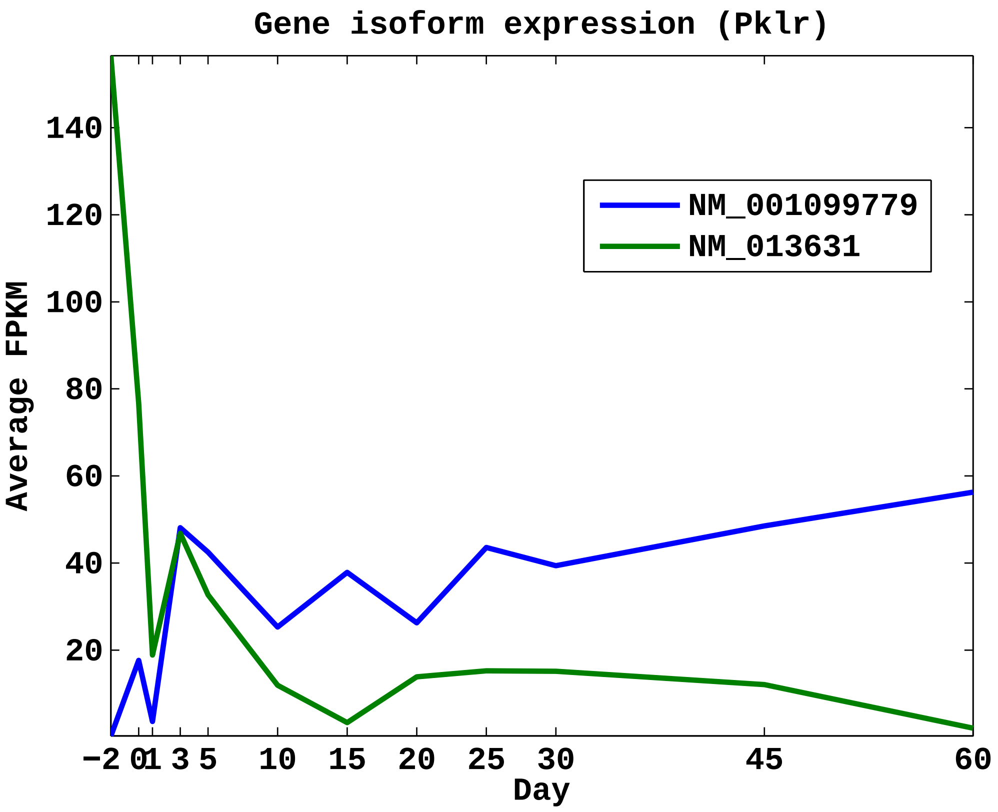

Gene isoform expression (Pklr)
NM_001099779
NM_013631
Average FPKM
140
120
100
80
60
40
20
−2
0
1
3
5
10
15
20
25
30
45
60
Day

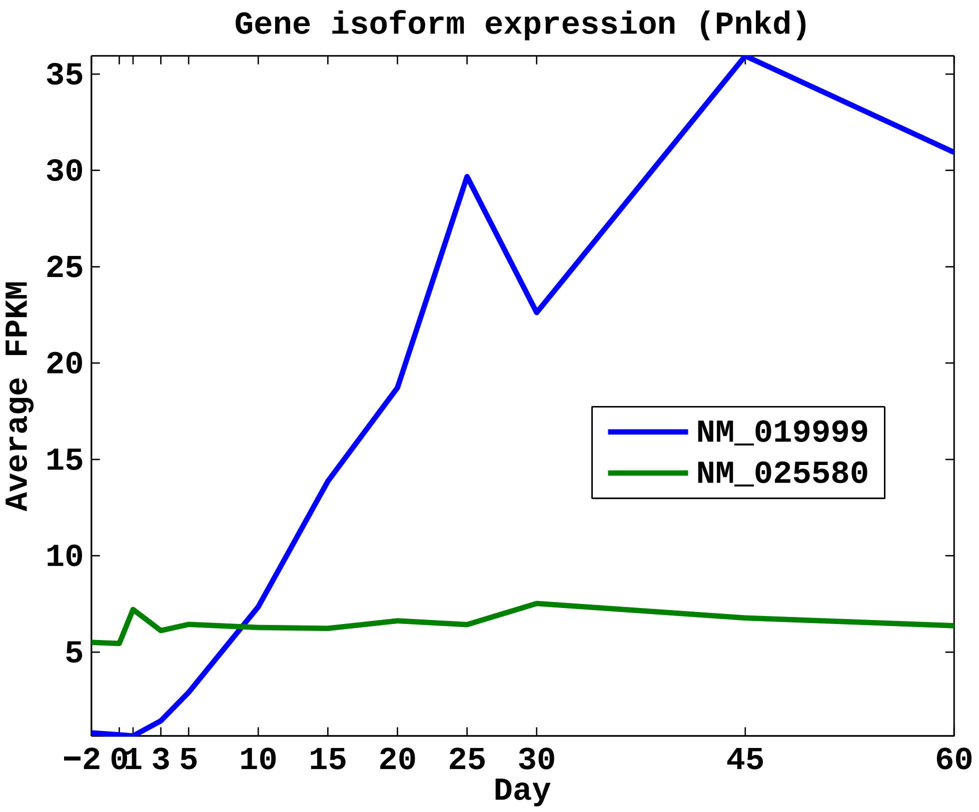
Gene isoform expression (Pnkd)
Average FPKM
35
30
25
20
15
10
5
−2
0
1
3
5
10
15
20
25
30
45
60
Day
NM_019999
NM_025580

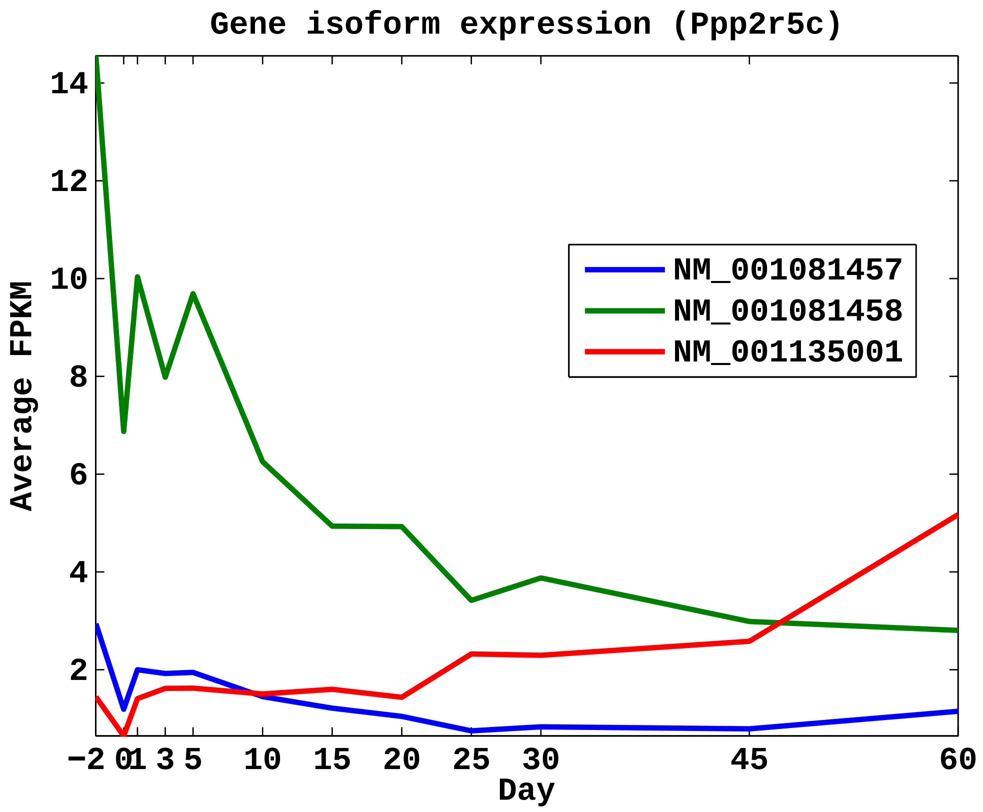

Gene isoform expression (Ppp2r5c)
Average FPKM
Day
14
12
10
8
6
4
2
−2 0 1 3 5 10 15 20 25 30 45 60
NM_001081457
NM_001081458
NM_001135001

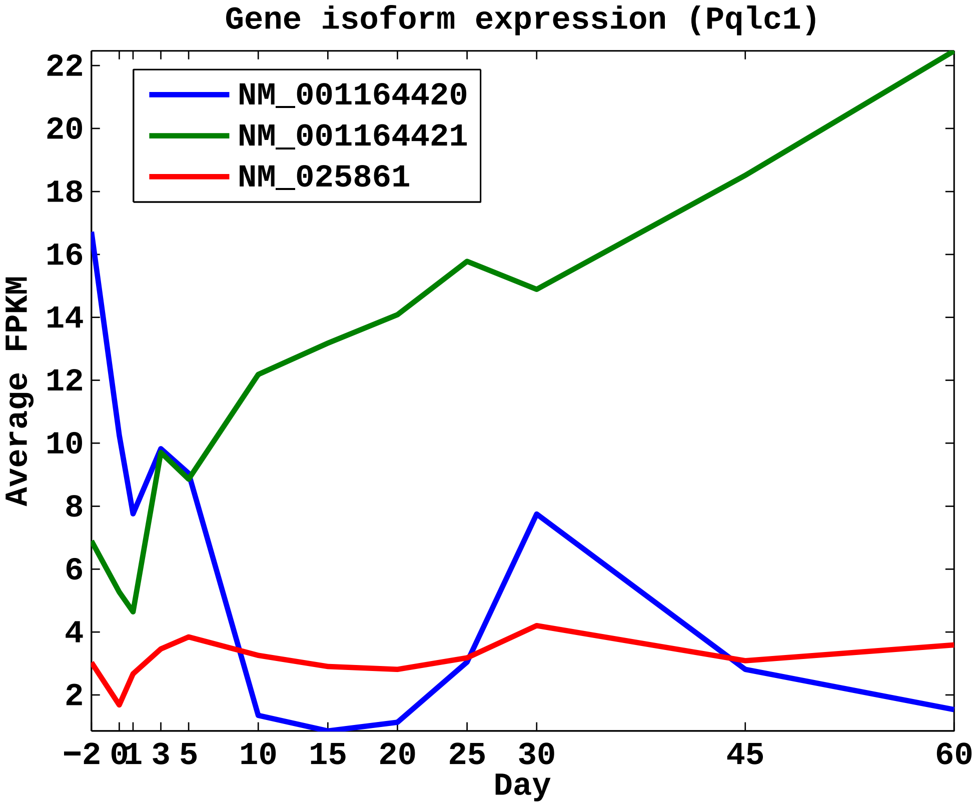

Gene isoform expression (Pqlc1)
NM_001164420
NM_001164421
NM_025861
Average FPKM
22
20
18
16
14
12
10
8
6
4
2
−2 0 1 3 5 10 15 20 25 30 45 60
Day

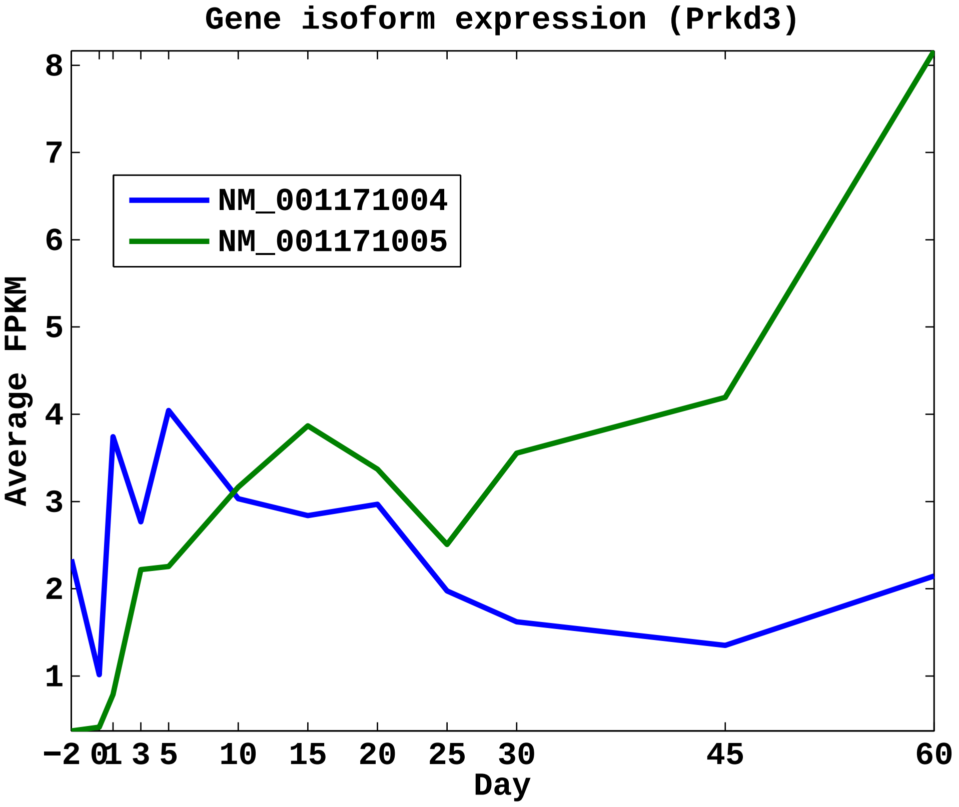
Gene isoform expression (Prkd3)
NM_001171004
NM_001171005
Average FPKM
Day
1
2
3
4
5
6
7
8
−2
0
1
3
5
10
15
20
25
30
45
60

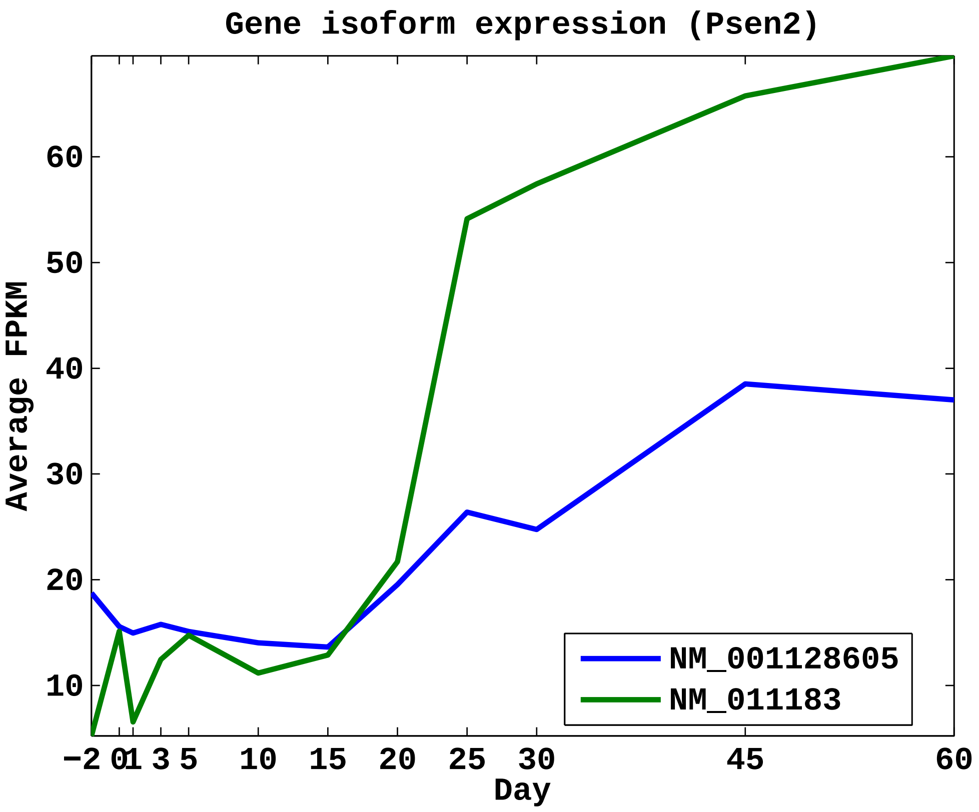
Gene isoform expression (Psen2)
Average FPKM
60
50
40
30
20
10
−2 0 1 3 5 10 15 20 25 30 45 60
Day
NM_001128605
NM_011183

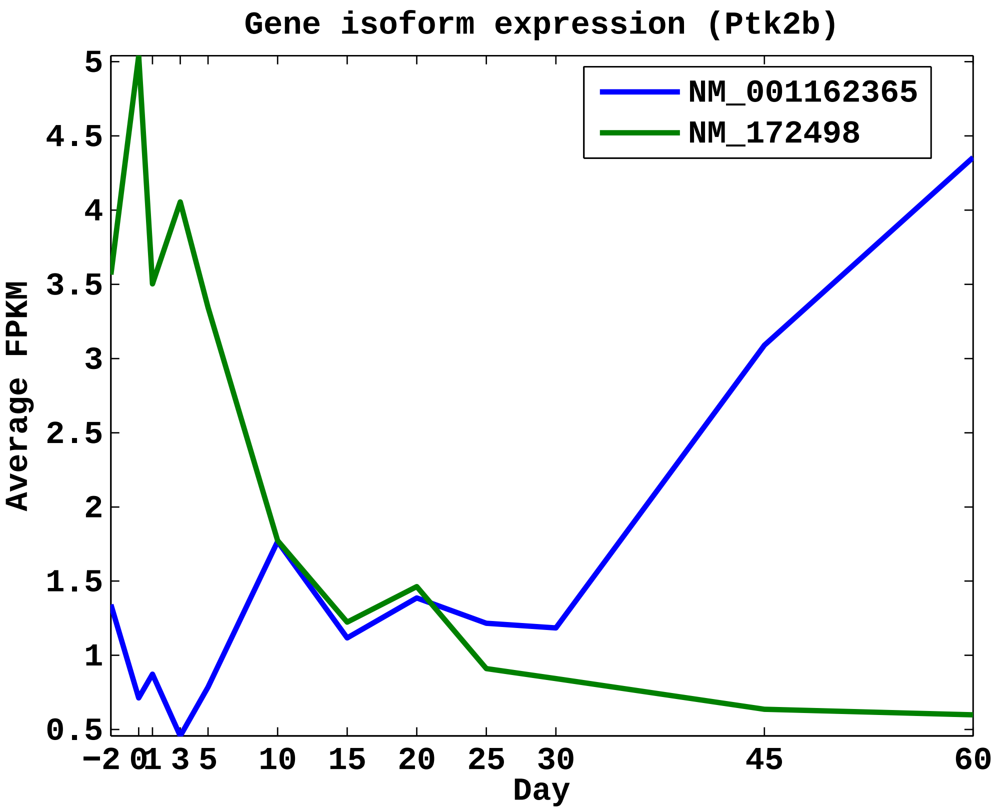

Gene isoform expression (Ptk2b)
NM_001162365
NM_172498
Average FPKM
5
4.5
4
3.5
3
2.5
2
1.5
1
0.5
−2 0 1 3 5 10 15 20 25 30 45 60
Day

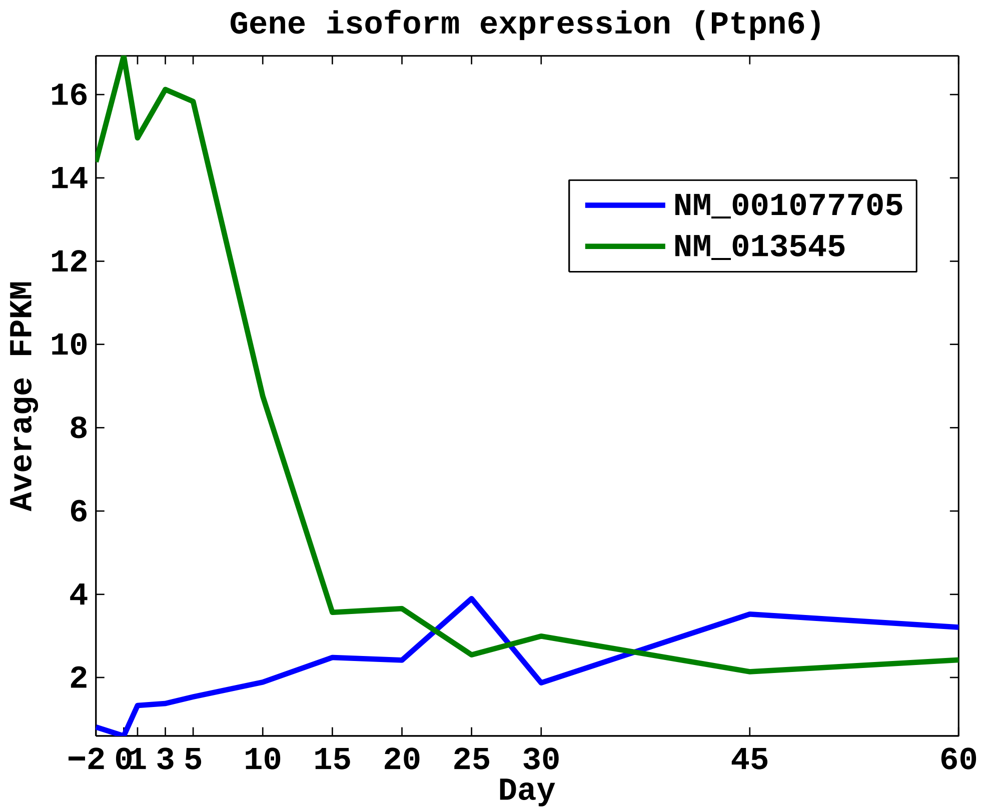

Gene isoform expression (Ptpn6)
Average FPKM
16
14
12
10
8
6
4
2
−2
0
1
3
5
10
15
20
25
30
45
60
Day
NM_001077705
NM_013545

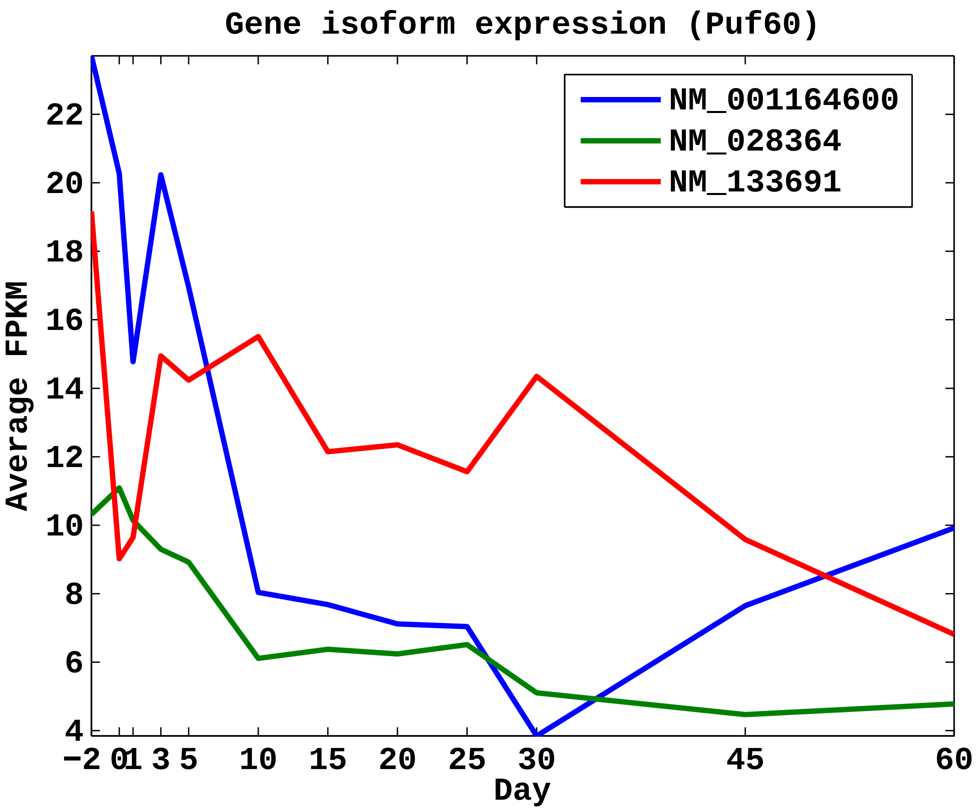
Gene isoform expression (Puf60)
NM_001164600
NM_028364
NM_133691
Average FPKM
22
20
18
16
14
12
10
8
6
4
−2 0 1 3 5 10 15 20 25 30 45 60
Day

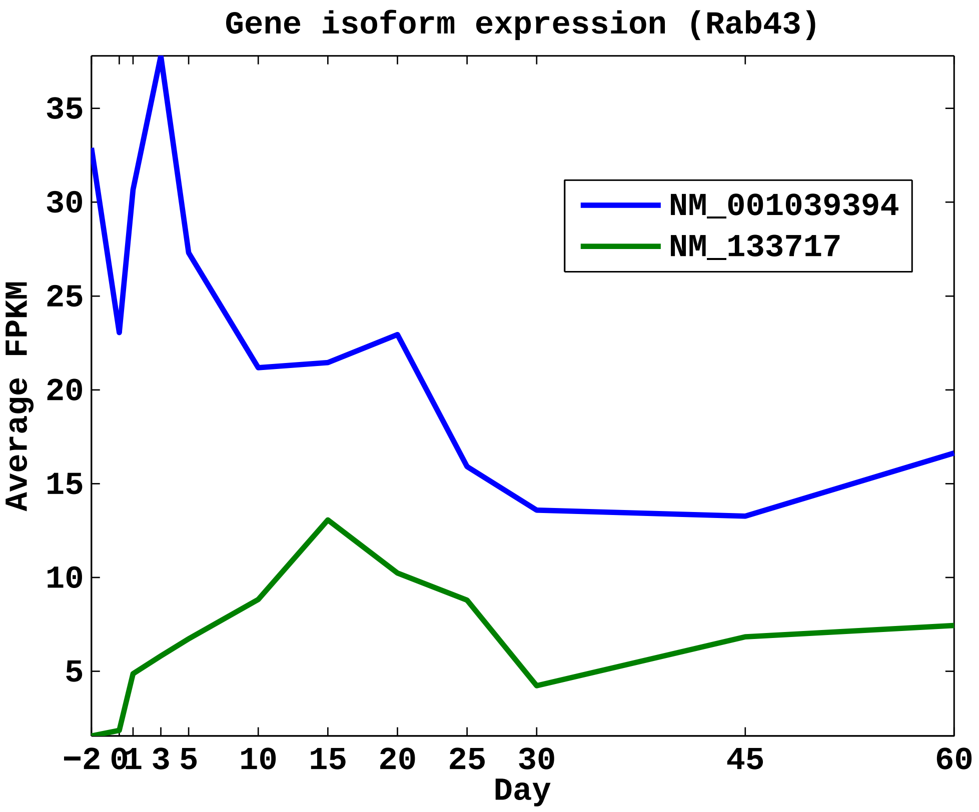
Gene isoform expression (Rab43)
NM_001039394
NM_133717
Average FPKM
5
10
15
20
25
30
35
−2
0
1
3
5
10
15
20
25
30
45
60
Day

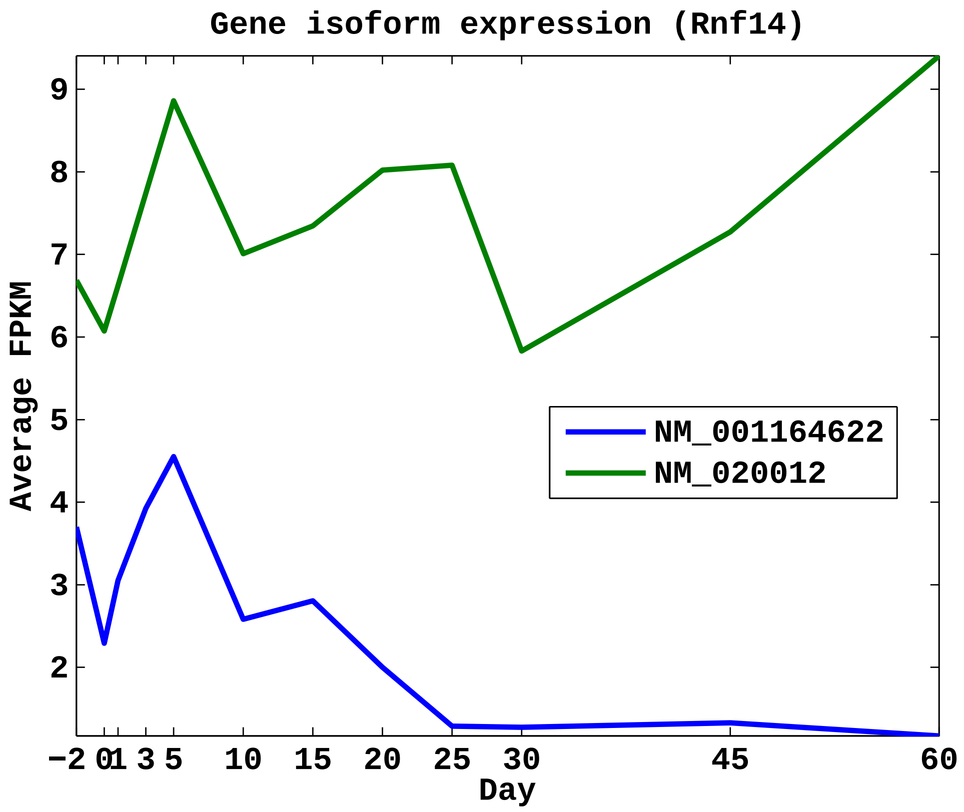
Gene isoform expression (Rnf14)
Average FPKM
9
8
7
6
5
4
3
2
−2
0
1
3
5
10
15
20
25
30
45
60
Day
NM_001164622
NM_020012

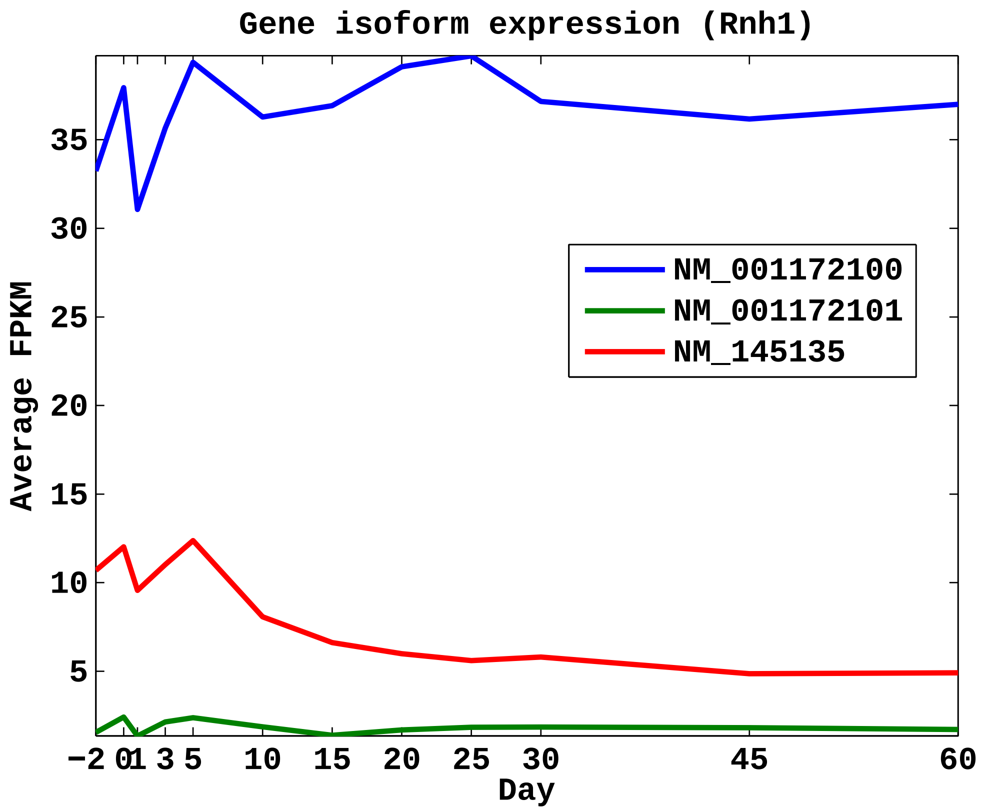
Gene isoform expression (Rnh1)
Average FPKM
35
30
25
20
15
10
5
−2 0 1 3 5 10 15 20 25 30 45 60
Day
NM_001172100
NM_001172101
NM_145135

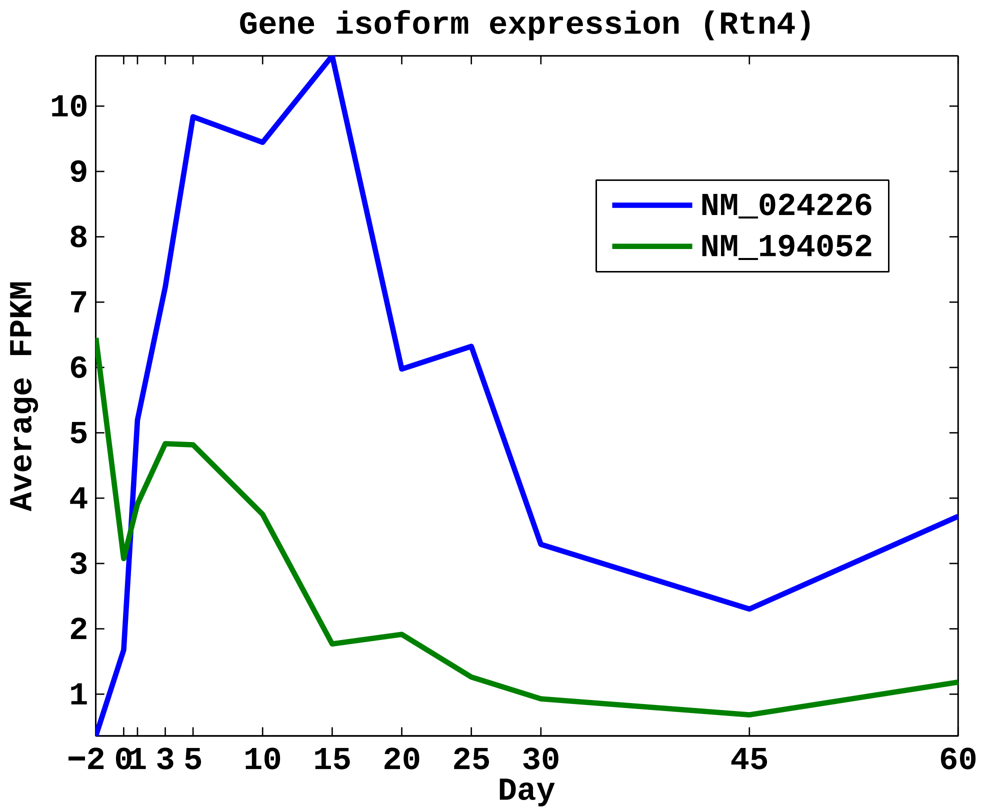

Gene isoform expression (Rtn4)
Average FPKM
10
9
8
7
6
5
4
3
2
1
−2
0
1
3
5
10
15
20
25
30
45
60
Day
NM_024226
NM_194052

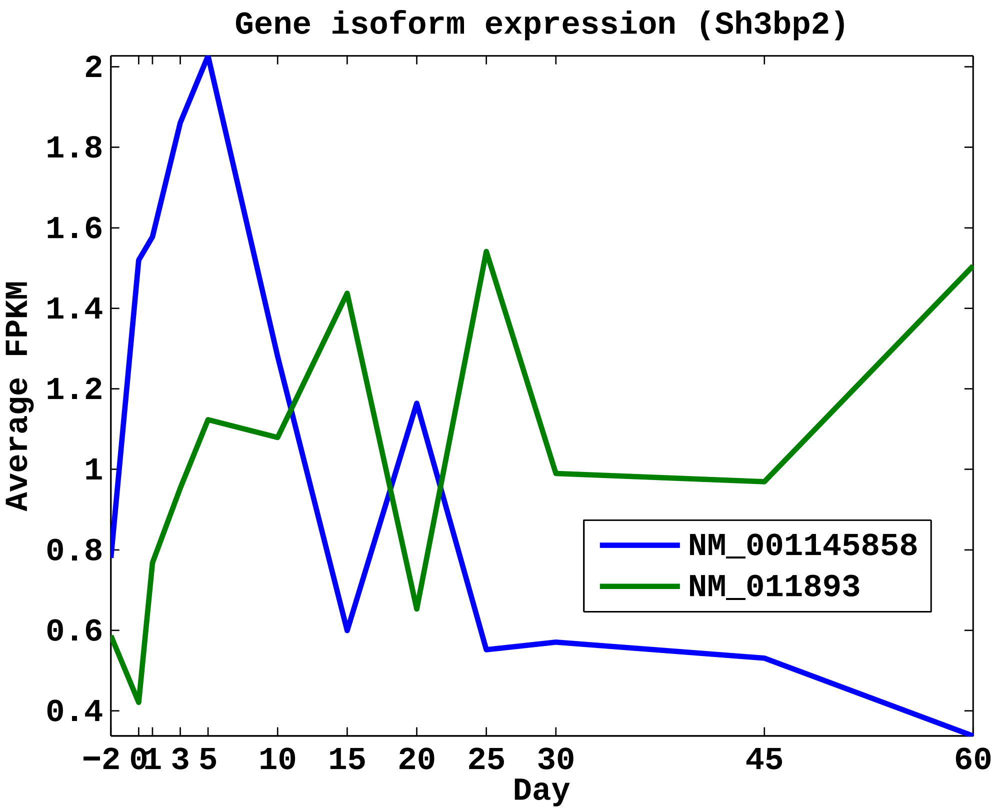

Gene isoform expression (Sh3bp2)
Average FPKM
2
1.8
1.6
1.4
1.2
1
0.8
0.6
0.4
−2
0
1
3
5
10
15
20
25
30
45
60
Day
NM_001145858
NM_011893

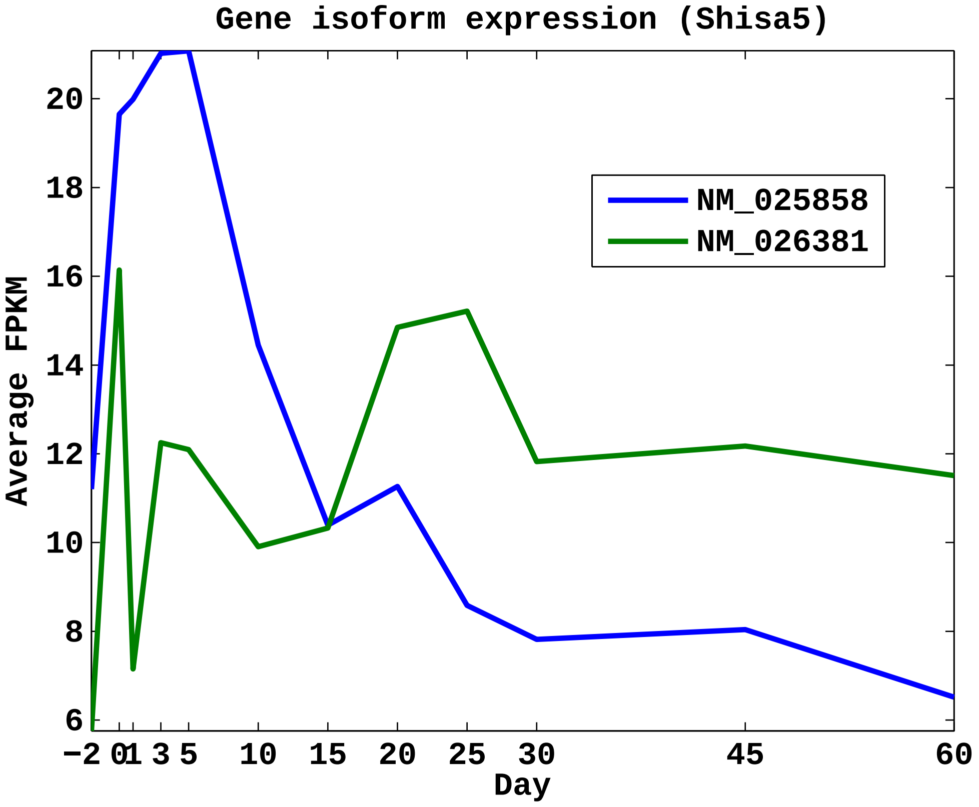
Gene isoform expression (Shisa5)
Average FPKM
Day
NM_025858
NM_026381
20
18
16
14
12
10
8
6
−2 0 1 3 5 10 15 20 25 30 45 60

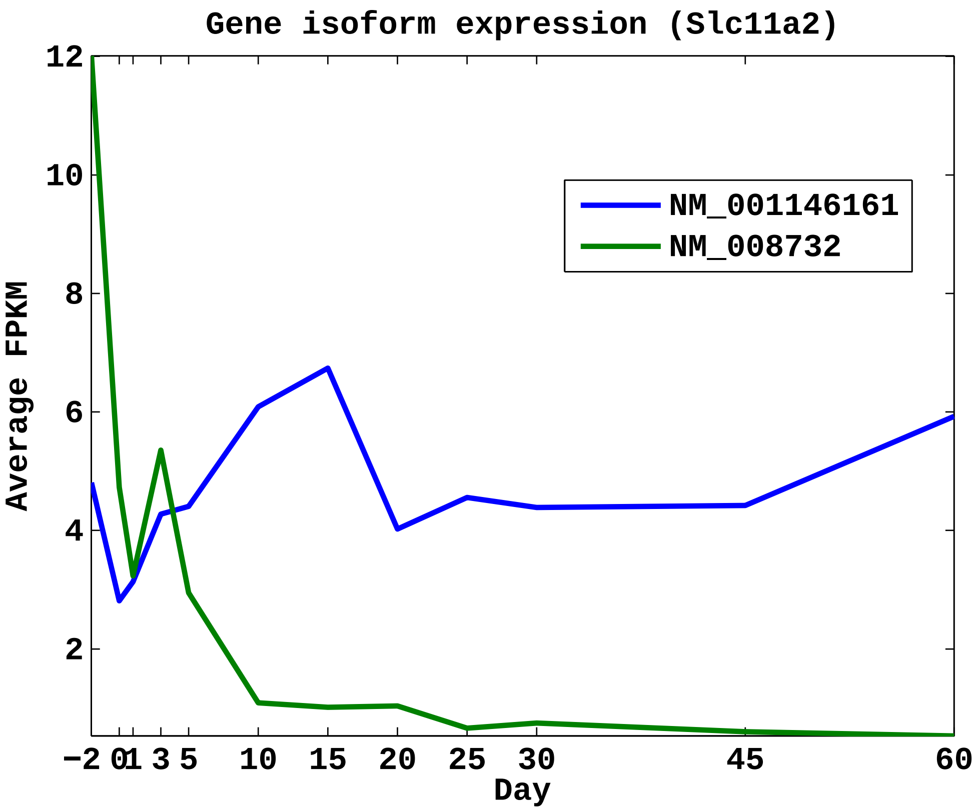

Gene isoform expression (Slc11a2)
Average FPKM
Day
NM_001146161
NM_008732
12
10
8
6
4
2
−2 0 1 3 5 10 15 20 25 30 45 60

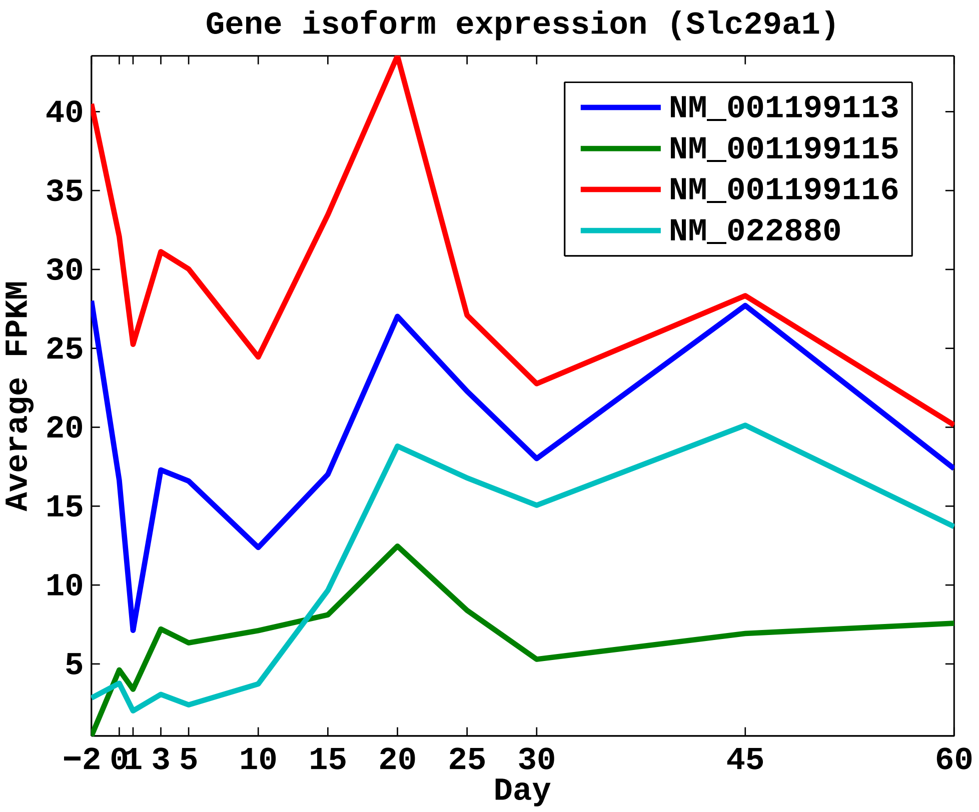
Gene isoform expression (Slc29a1)
NM_001199113
NM_001199115
NM_001199116
NM_022880
Average FPKM
40
35
30
25
20
15
10
5
−2
0
1
3
5
10
15
20
25
30
45
60
Day

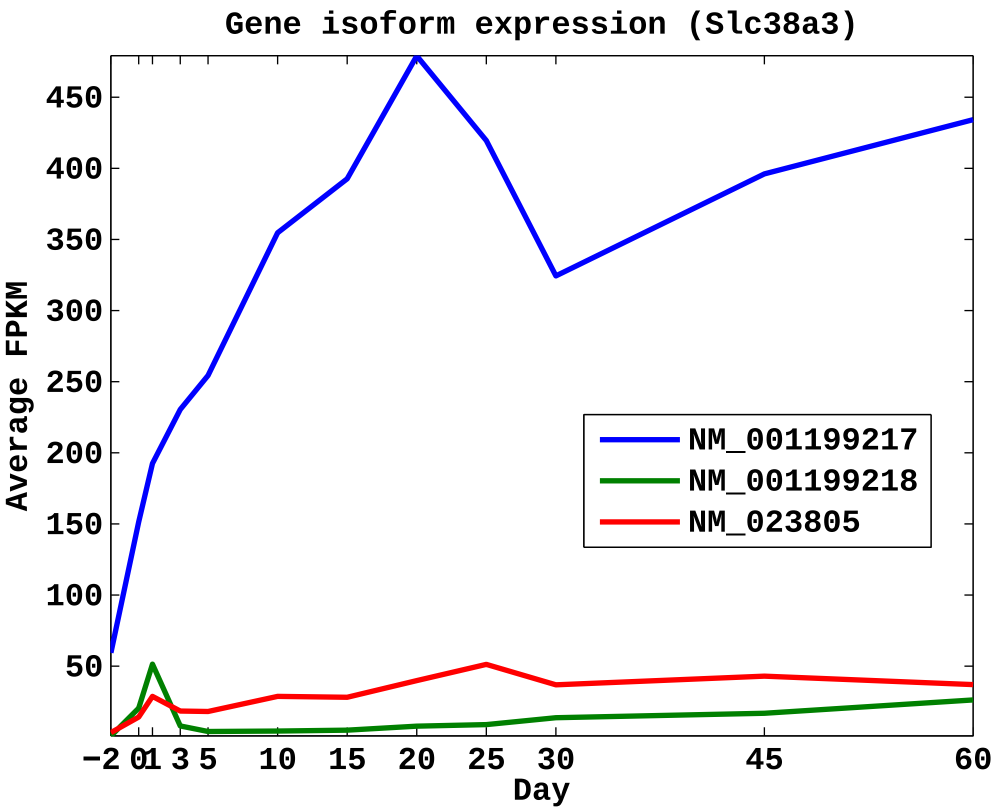
Gene isoform expression (Slc38a3)
Average FPKM
450
400
350
300
250
200
150
100
50
−2 0 1 3 5 10 15 20 25 30 45 60
Day
NM_001199217
NM_001199218
NM_023805

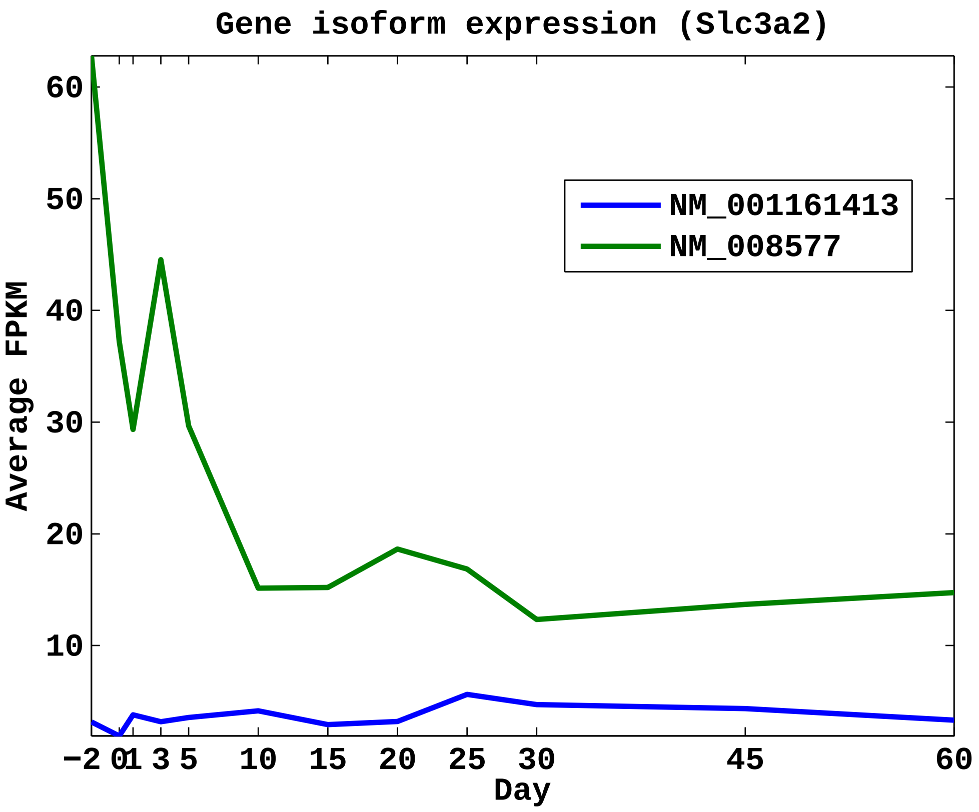

Gene isoform expression (Slc3a2)
Average FPKM
60
50
40
30
20
10
NM_001161413
NM_008577
−2
0
1
3
5
10
15
20
25
30
45
60
Day

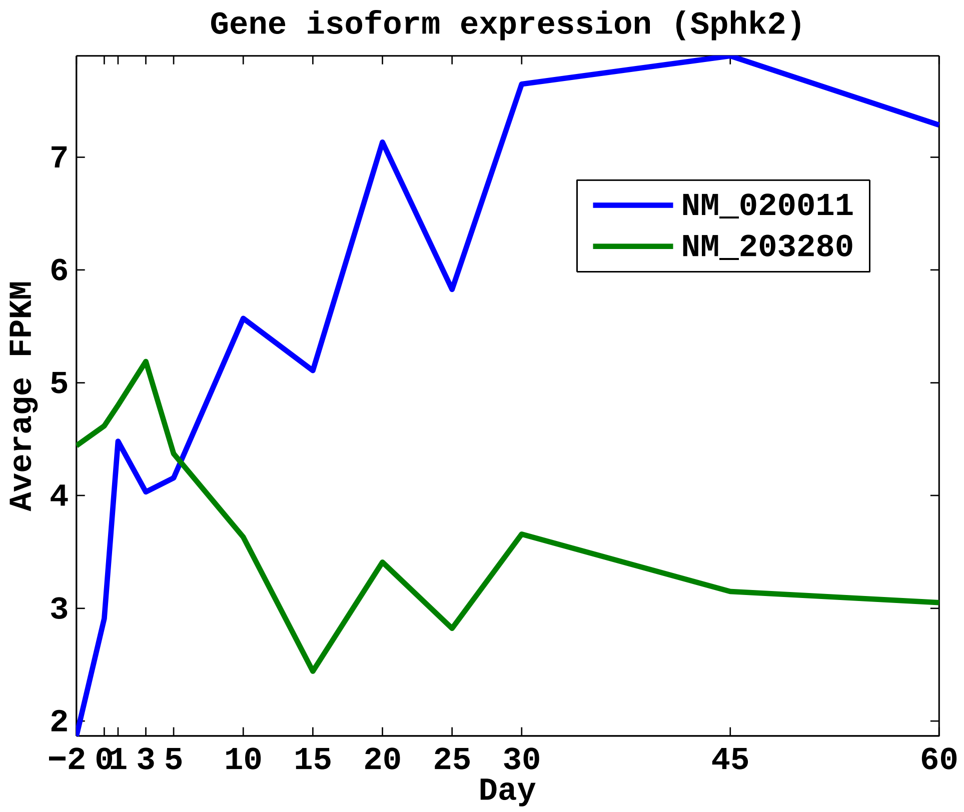

Gene isoform expression (Sphk2)
Average FPKM
Day
NM_020011
NM_203280
2
3
4
5
6
7
−2
0
1
3
5
10
15
20
25
30
45
60

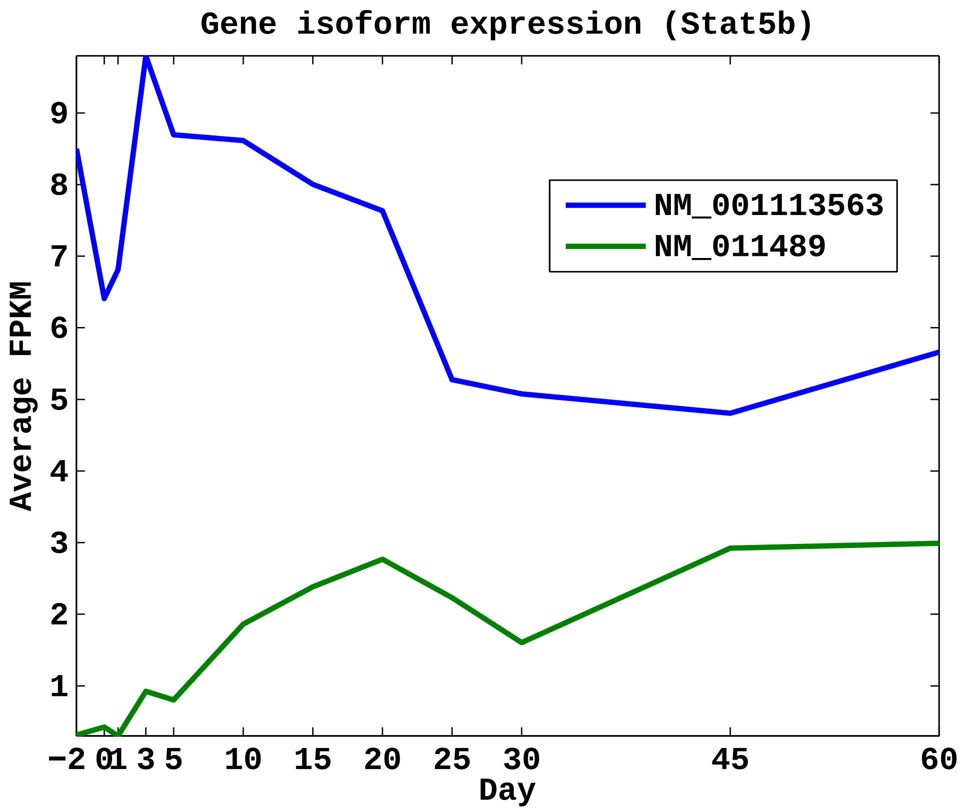
Gene isoform expression (Stat5b)
NM_001113563
NM_011489
Average FPKM
Day
9
8
7
6
5
4
3
2
1
−2
0
1
3
5
10
15
20
25
30
45
60

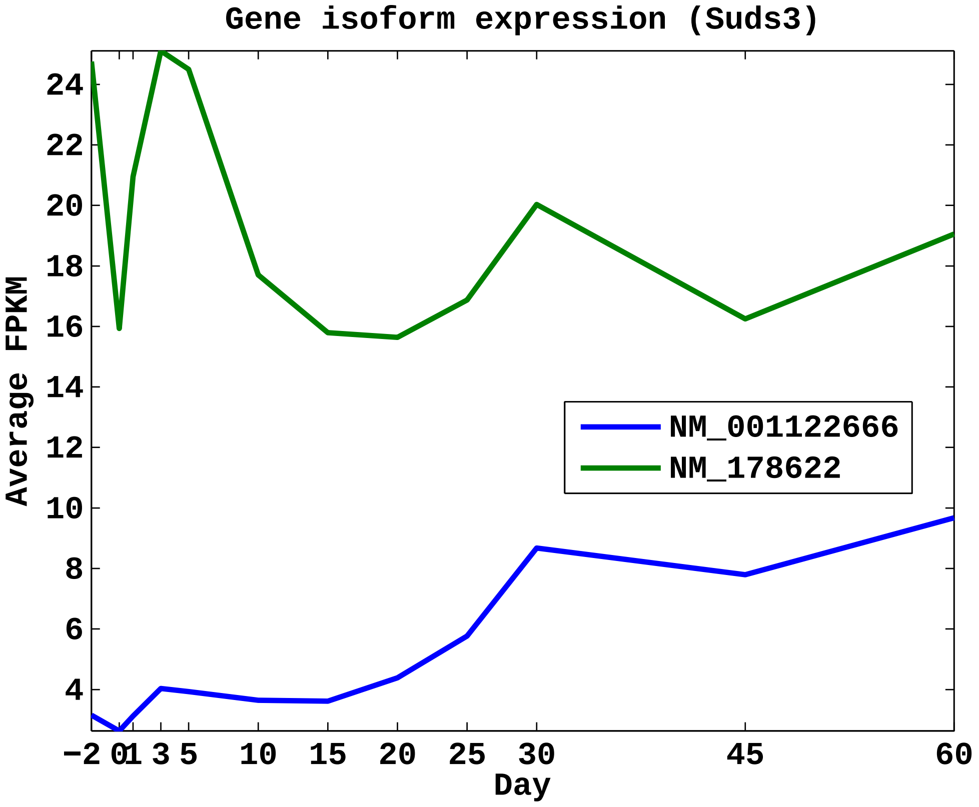
Gene isoform expression (Suds3)
Average FPKM
24
22
20
18
16
14
12
10
8
6
4
−2
0
1
3
5
10
15
20
25
30
45
60
Day
NM_001122666
NM_178622

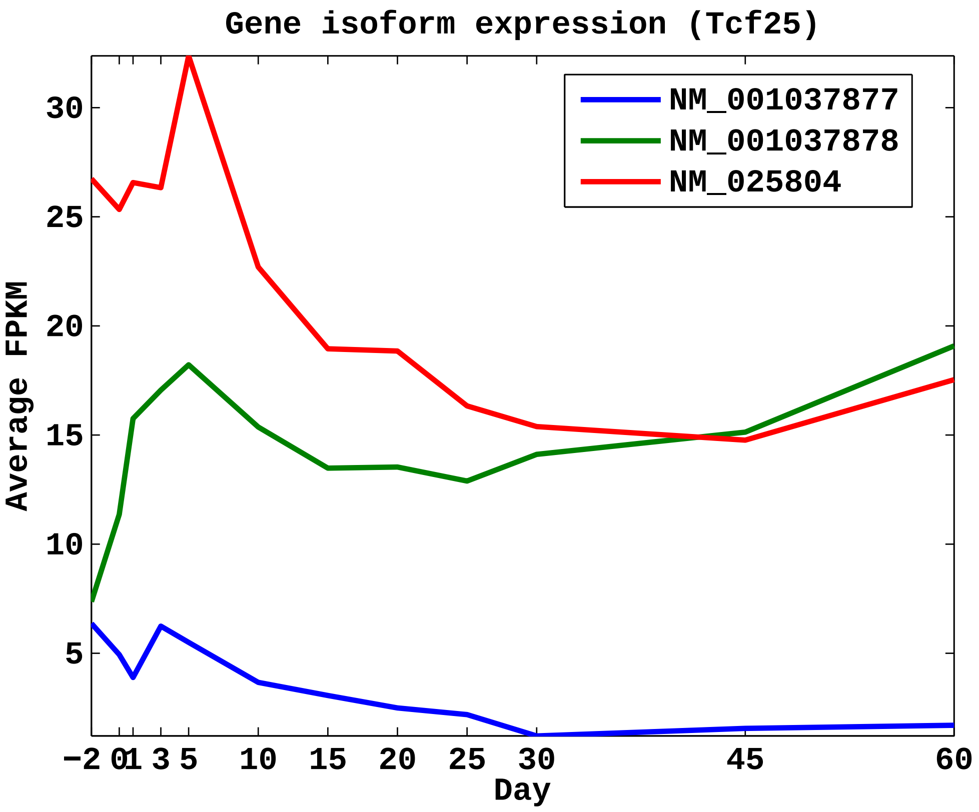
Gene isoform expression (Tcf25)
NM_001037877
NM_001037878
NM_025804
Average FPKM
30
25
20
15
10
5
−2
0
1
3
5
10
15
20
25
30
45
60
Day

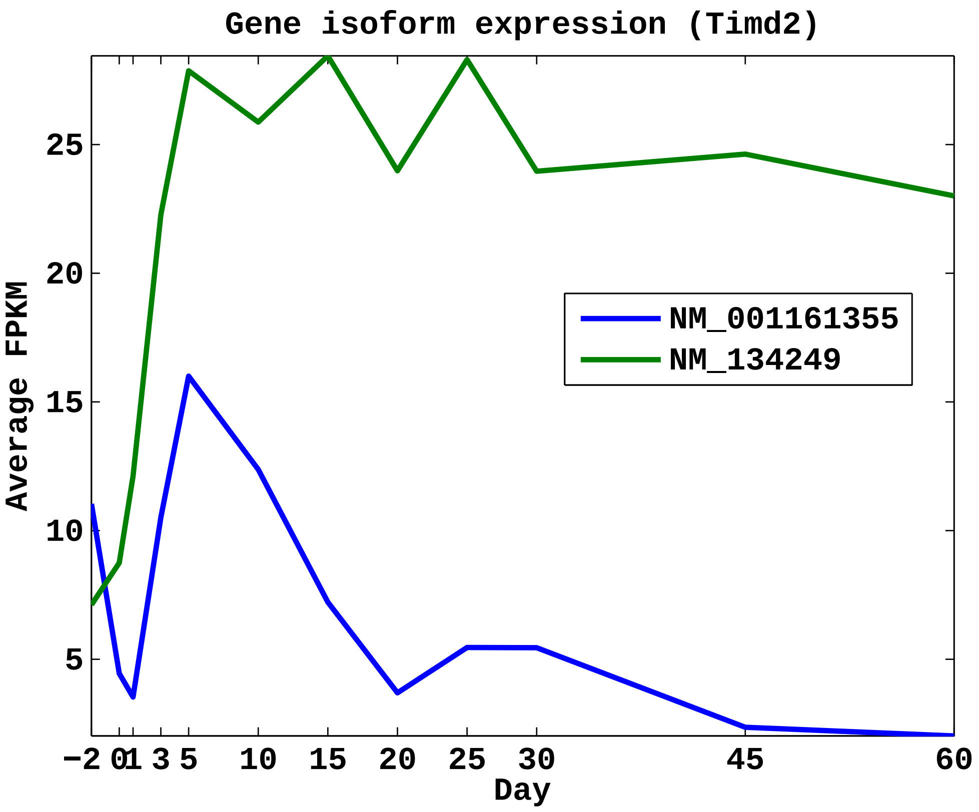

Gene isoform expression (Timd2)
Average FPKM
25
20
15
10
5
−2
0
1
3
5
10
15
20
25
30
45
60
Day
NM_001161355
NM_134249

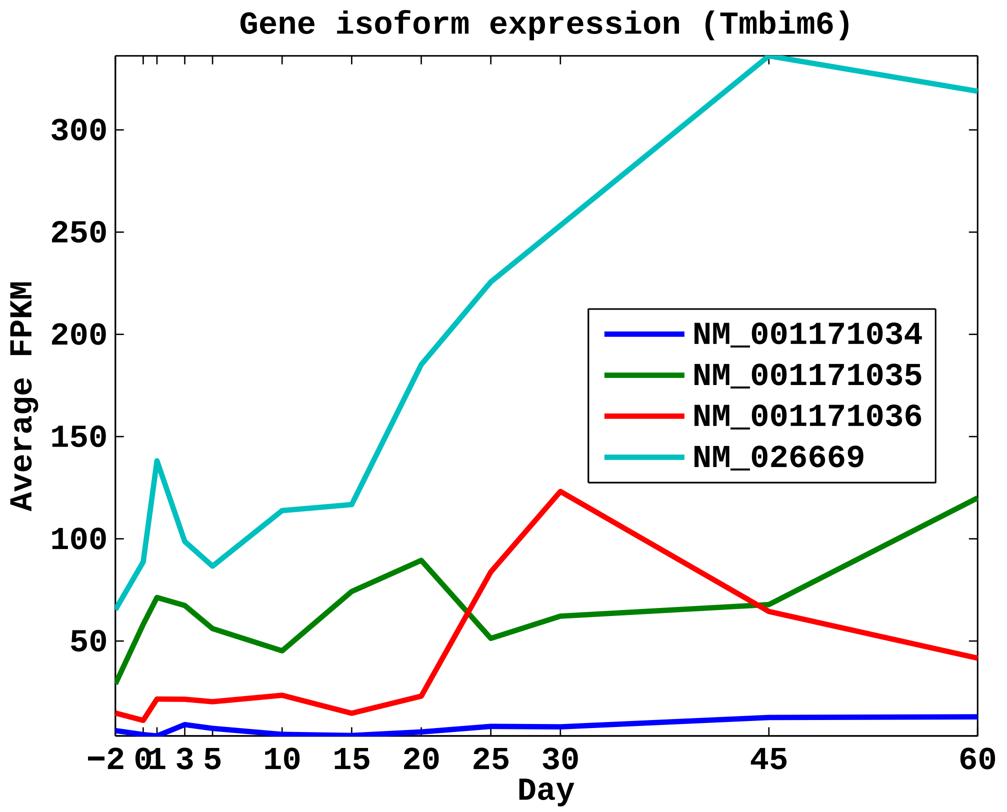

Gene isoform expression (Tmbim6)
Average FPKM
300
250
200
150
100
50
−2 0 1 3 5 10 15 20 25 30 45 60
Day
NM_001171034
NM_001171035
NM_001171036
NM_026669

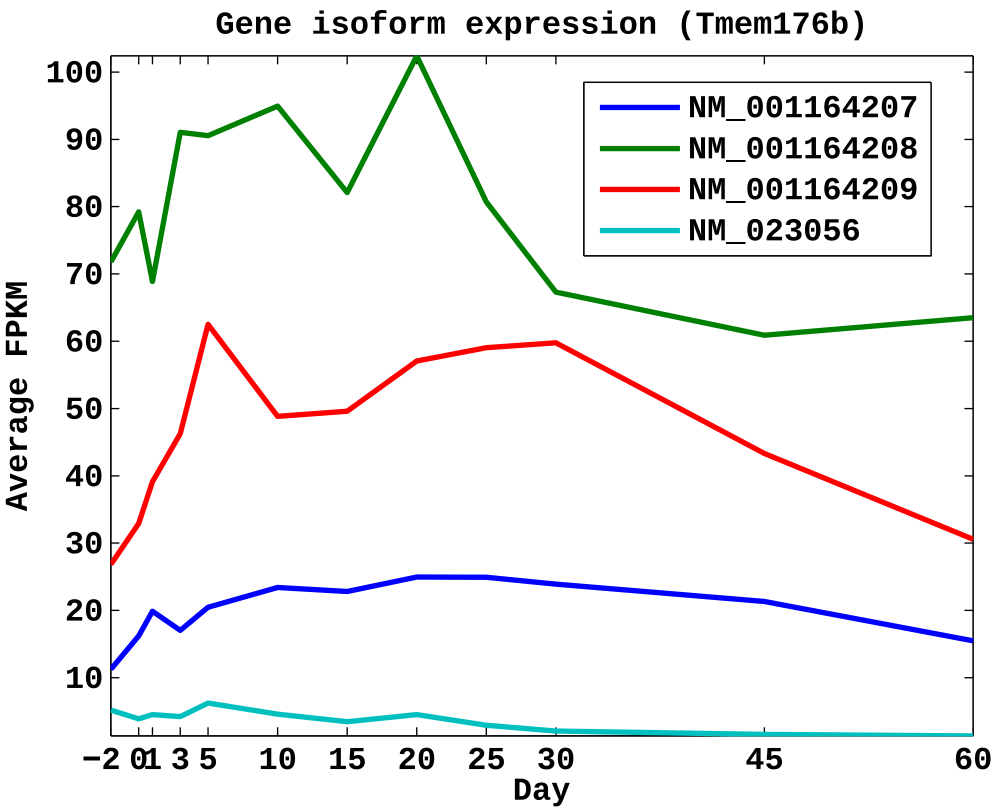

Gene isoform expression (Tmem176b)
Average FPKM
Day
NM_001164207
NM_001164208
NM_001164209
NM_023056
100
90
80
70
60
50
40
30
20
10
−2
0
1
3
5
10
15
20
25
30
45
60

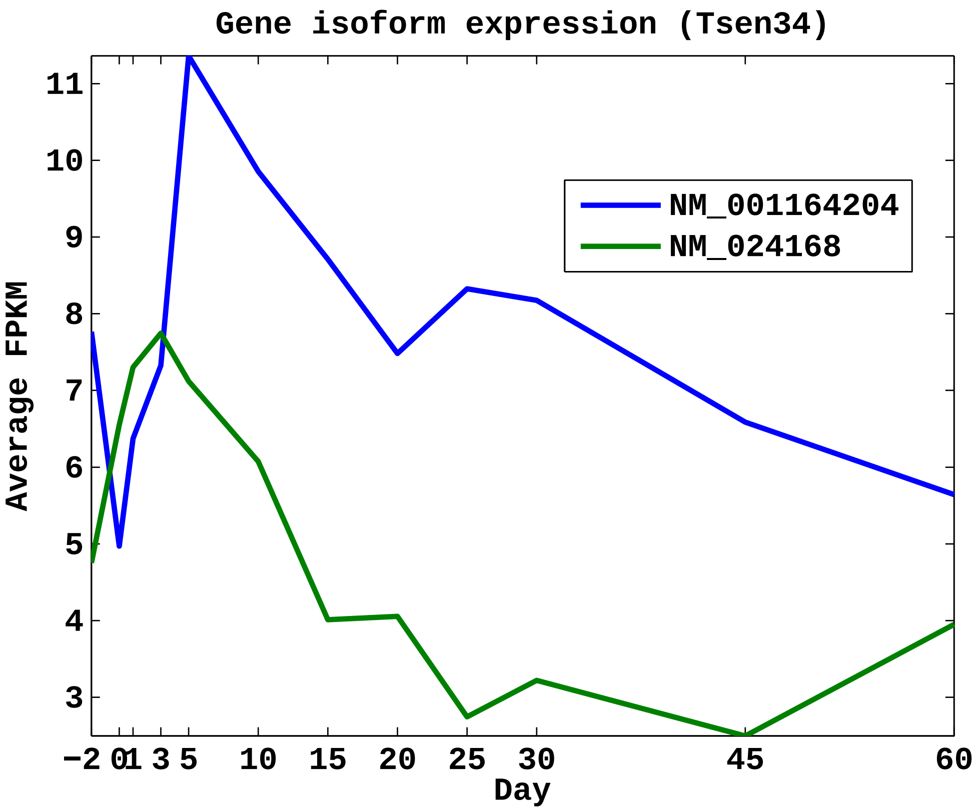
Gene isoform expression (Tsen34)
NM_001164204
NM_024168
Average FPKM
11
10
9
8
7
6
5
4
3
−2
0
1
3
5
10
15
20
25
30
45
60
Day

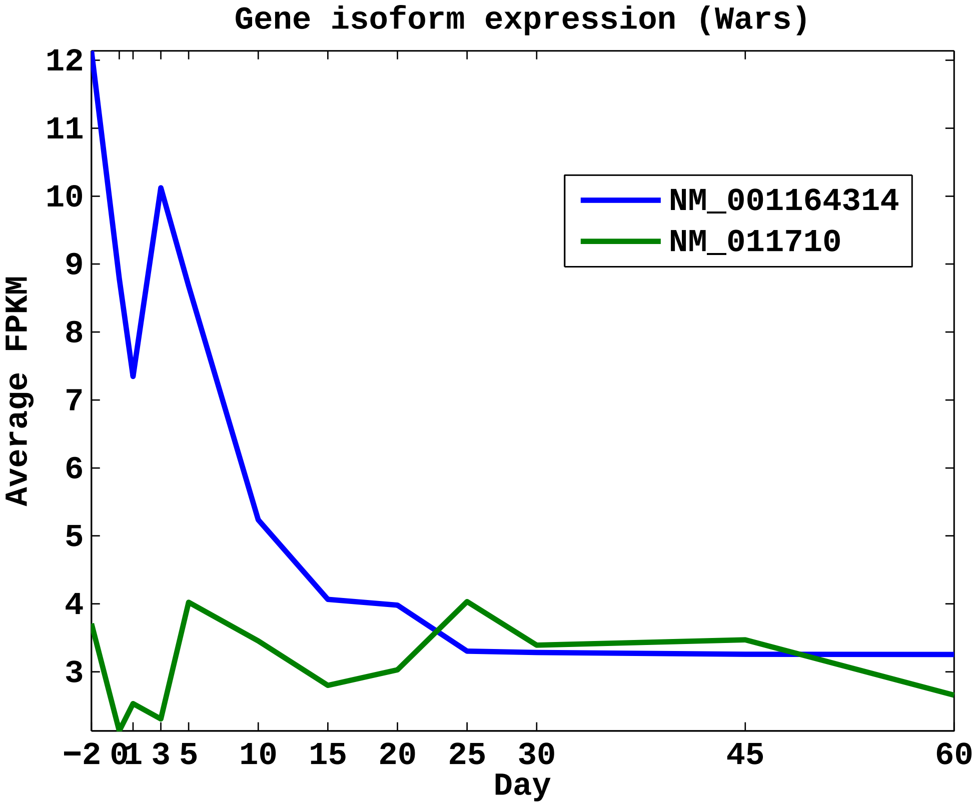

Gene isoform expression (Wars)
Average FPKM
12
11
10
9
8
7
6
5
4
3
−2
0
1
3
5
10
15
20
25
30
45
60
Day
NM_001164314
NM_011710

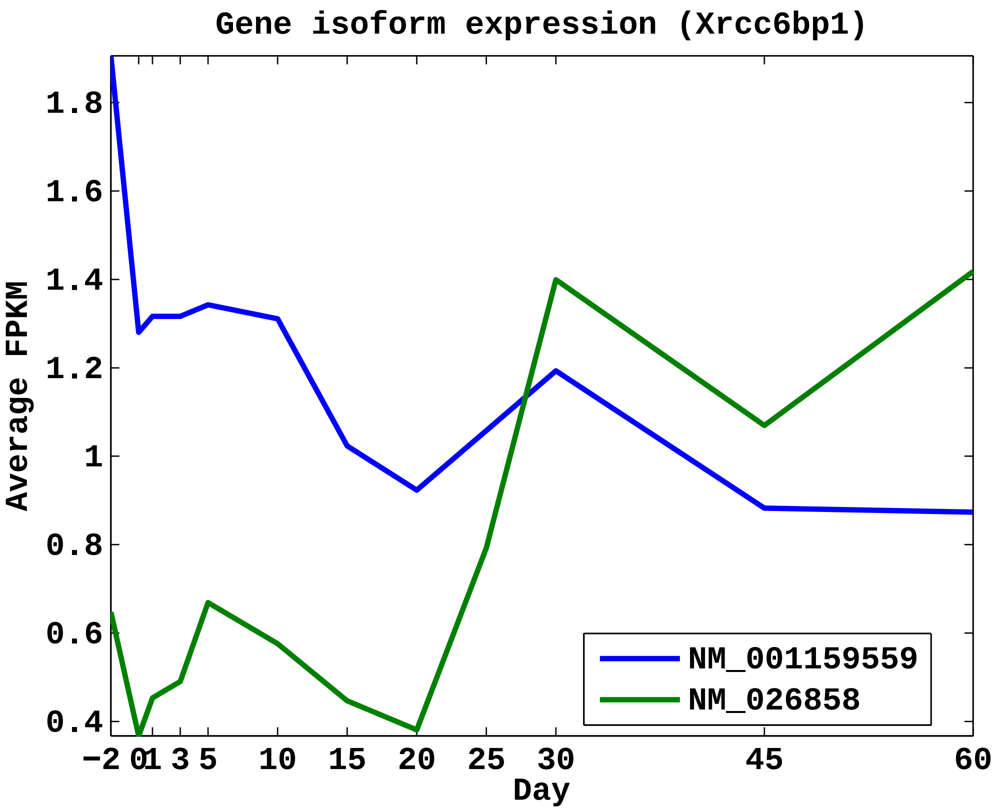
Gene isoform expression (Xrcc6bp1)
Average FPKM
1.8
1.6
1.4
1.2
1
0.8
0.6
0.4
−2 0 1 3 5 10 15 20 25 30 45 60
Day
NM_001159559
NM_026858
