# Supplementary material for: Deciphering the Developmental Dynamics of the Mouse Liver Transcriptome
Source: PLoS One. 2015 Oct 23;10(10):e0141220. doi: 10.1371/journal.pone.0141220 (PMC4619800; doi:10.1371/journal.pone.0141220)

Supplemental Methods

Gene Expression

- Gene expression is measured in FPKM (fragments per kilobase of exon per million fragments mapped)

$$\text{FPKM} = \frac{\text{number of reads in region}}{\frac{\text{total reads}}{1000000} \times \frac{\text{region length}}{1000}}$$

(Mortazavi et al. Nature Methods,2008)

- The population distribution of FPKM data can be described by a Poisson probability distribution

- Presence of Extra-Poisson variation
  - Effects standard error
  - Effects p-values

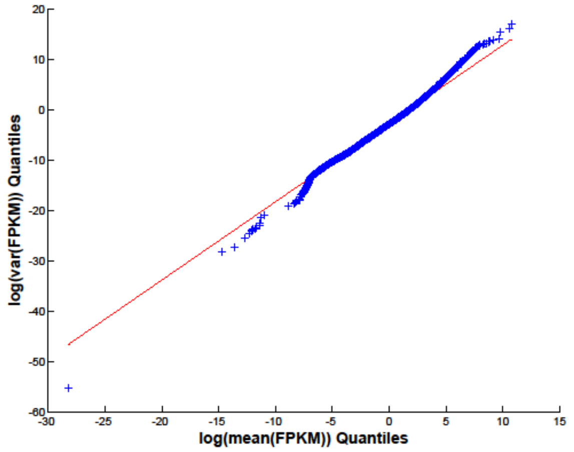

Measuring Significance of Gene Expression

- Fit a Poisson log-linear model that permits extra-Poisson variation

$$\mu\{Y_i\} = \mu_i$$

$$Var\{Y_i\} = \psi\mu_i$$

$$\log(\mu_i) = \beta$$

Dispersion parameter

- The dispersion parameter accounts for extra-Poisson variation
- If the variance is like Poisson variance, then $\psi=1$
- Maximum likelihood estimates of β
  - Provides significance estimates of parameters
- Adjust for multiple hypothesis testing
  - Benjamini–Hochberg procedure

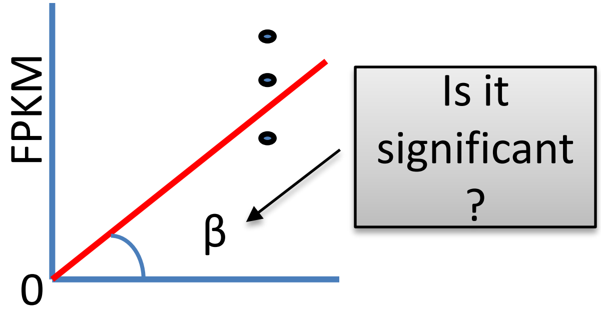

Quasi-Likelihood Approach

- The standard errors of the maximum quasi-likelihood estimates are $\sqrt{\hat{\Psi}}$ times the standard errors of the maximum likelihood estimates.
- Considers both the size and variation in expression in determining significance.
- Tries to answers the question: how different is the observed expression from the background (no expression).

Select genes expressed significantly (FDR ≤ 0.05) in at least one of the 12 ages

9945 genes with significant expression in at least one age

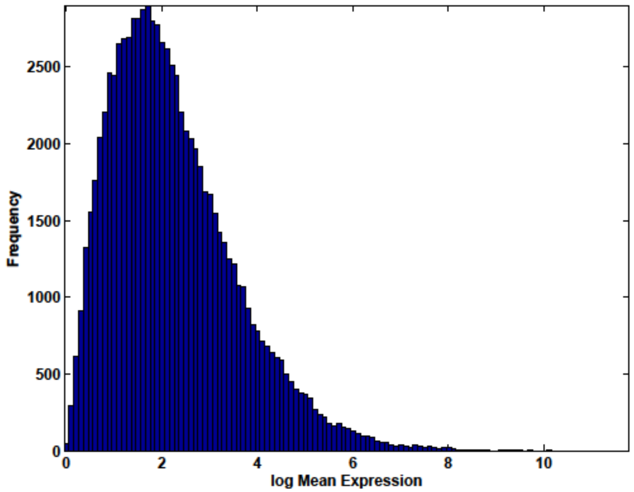

Measuring Significant Differential Expression

- Is the gene expressed differently in the different ages?
- Fit a Poisson-log-linear regression model
- Use the Drop-in-Deviance F-Test (Accounts for extra-Poisson variation)

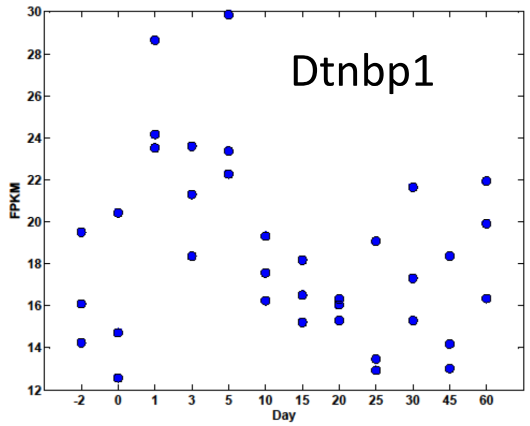

$$\log(\mu_i) = \beta_0 + \beta_i AGE_i \quad \text{Full model}$$

$$\log(\mu_i) = \beta_0 \quad \text{Reduced model}$$

$$\text{F} - \text{stat} = \frac{\text{Drop in deviance / Drop in d.f.}}{\hat{\psi}}$$

$$\text{Dispersion parameter} = \frac{\text{Deviance}}{\text{Degrees of freedom}}$$

- Adjust for multiple hypothesis testing
  - Benjamini–Hochberg procedure

How Many Factors to Select

- Use subjective judgment by observing the data.
- Examine the Scree plot of the eigenvalues from principal component analysis.
- Perform a Parallel Analysis to determine the number of factors to retain.
- Retained 3 factors based on these guides.

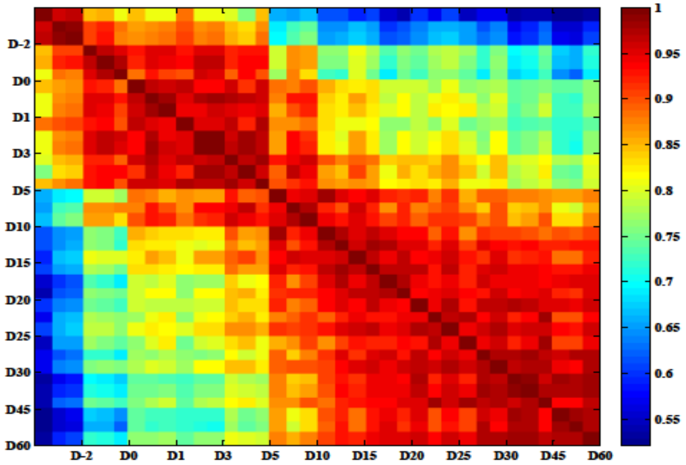

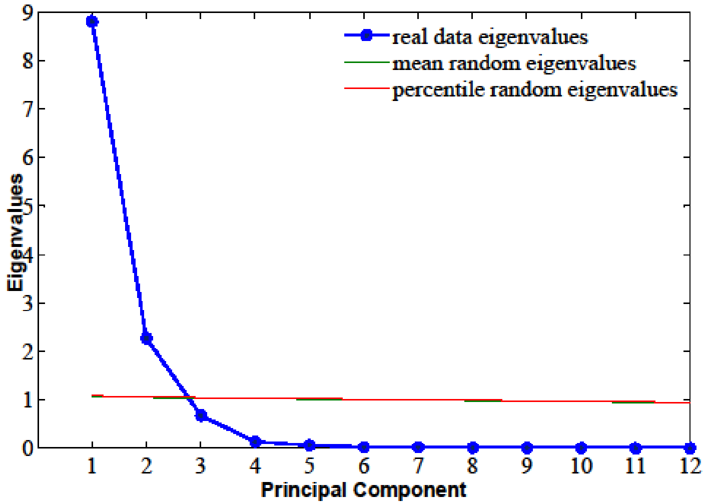

Supplement: S1 Methods — (PDF) [file pone.0141220.s006.pdf]
